# Computational Modeling of Genetic Contributions to Excitability and Neural Coding in Layer V Pyramidal Cells: Applications to Schizophrenia Pathology

**DOI:** 10.3389/fncom.2019.00066

**Published:** 2019-09-26

**Authors:** Tuomo Mäki-Marttunen, Anna Devor, William A. Phillips, Anders M. Dale, Ole A. Andreassen, Gaute T. Einevoll

**Affiliations:** ^1^Simula Research Laboratory, Oslo, Norway; ^2^Department of Neurosciences, University of California San Diego, La Jolla, CA, United States; ^3^Department of Radiology, University of California San Diego, La Jolla, CA, United States; ^4^Martinos Center for Biomedical Imaging, Harvard Medical School, Massachusetts General Hospital, Charlestown, MA, United States; ^5^Psychology, Faculty of Natural Sciences, University of Stirling, Stirling, United Kingdom; ^6^NORMENT, Division of Mental Health and Addiction, Oslo University Hospital and Institute of Clinical Medicine, University of Oslo, Oslo, Norway; ^7^Faculty of Science and Technology, Norwegian University of Life Sciences, Ås, Norway; ^8^Department of Physics, University of Oslo, Oslo, Norway

**Keywords:** voltage-gated ion channel gene, schizophrenia genetics, cortical excitability, biophysical modeling, functional genetics, neuronal code, prepulse inhibition, spatiotemporal integration

## Abstract

Pyramidal cells in layer V of the neocortex are one of the most widely studied neuron types in the mammalian brain. Due to their role as integrators of feedforward and cortical feedback inputs, they are well-positioned to contribute to the symptoms and pathology in mental disorders—such as schizophrenia—that are characterized by a mismatch between the internal perception and external inputs. In this modeling study, we analyze the input/output properties of layer V pyramidal cells and their sensitivity to modeled genetic variants in schizophrenia-associated genes. We show that the excitability of layer V pyramidal cells and the way they integrate inputs in space and time are altered by many types of variants in ion-channel and Ca^2+^ transporter-encoding genes that have been identified as risk genes by recent genome-wide association studies. We also show that the variability in the output patterns of spiking and Ca^2+^ transients in layer V pyramidal cells is altered by these model variants. Importantly, we show that many of the predicted effects are robust to noise and qualitatively similar across different computational models of layer V pyramidal cells. Our modeling framework reveals several aspects of single-neuron excitability that can be linked to known schizophrenia-related phenotypes and existing hypotheses on disease mechanisms. In particular, our models predict that single-cell steady-state firing rate is positively correlated with the coding capacity of the neuron and negatively correlated with the amplitude of a prepulse-mediated adaptation and sensitivity to coincidence of stimuli in the apical dendrite and the perisomatic region of a layer V pyramidal cell. These results help to uncover the voltage-gated ion-channel and Ca^2+^ transporter-associated genetic underpinnings of schizophrenia phenotypes and biomarkers.

## 1. Introduction

Pyramidal cells constitute the majority of neurons in the mammalian cerebral cortex and play an important role in cognitive processes (Elston, 2003; Spruston, 2008). Layer V pyramidal cells (L5PCs) extend their apical dendrites throughout the cortical thickness of the neocortex and integrate information from local and distant sources (Binzegger et al., 2004; Larkum, 2013). Alterations in the L5PC excitability and its ability to process context- and sensory drive-dependent inputs have been proposed as a cause for hallucinations and other impairments of sensory perceptions related to mental disease (Larkum, 2013; Phillips and Silverstein, 2013; Phillips et al., 2016). In line with this hypothesis, genetic variants in voltage-gated ion channel-encoding genes and their altered expression have been associated with the risk of mental disorders (Green et al., 2010; Smolin et al., 2012). In this work, we use computational models of L5PCs to systematically study the impact of small-effect variants on L5PC excitability and phenotypes associated with schizophrenia (SCZ).

SCZ is a highly heritable mental disorder (heritability estimates range from 0.6–0.8; Ripke et al., 2013) with an estimated 0.3–0.7% prevalence in the world population (Saha et al., 2005). Recent genome-wide association studies (GWASs) have identified a large number of genetic loci involved in the risk of the mental disease (Ripke et al., 2014). These loci implicate hundreds of susceptibility genes, many of which encode voltage-gated ion channels and Ca^2+^ transporters (Ripke et al., 2014). In this work, we use our recently developed computational framework (Mäki-Marttunen et al., 2016) for studying how the integration of information in L5PCs may be distorted in SCZ.

In our previous work (Mäki-Marttunen et al., 2016), we showed that variants of SCZ-associated genes affect the excitability of the model neuron of Hay et al. (2011) and its responses to dendritic inputs. In particular, we showed that many variants alter the way the neuron responds to a second stimulus with a close resemblance to the deficits in prepulse inhibition (PPI; Mäki-Marttunen et al., 2016). PPI of the startle response is a widely applied behavioral test, where an auditory pre-stimulus inhibits the startle response to a second (stronger) stimulus when presented 30–300 ms in advance (Turetsky et al., 2007). Deficits in PPI are associated with SCZ (Turetsky et al., 2007) and to a lesser degree with bipolar disorder (Gogos et al., 2009). Although statistical genetics and GWASs have helped to make associations between gene variants and disease phenotypes, the mechanisms of PPI deficits and other circuit dysfunctions related to SCZ are incompletely understood at the cellular level.

In this work, we aim at bridging the gap of knowledge between SCZ genetics and disease phenotypes by using biophysically detailed models to uncover the influence of SCZ-associated genes on integration of information in L5PCs. L5PC population displays a wide diversity of morphological and electrophysiological behaviors (Chagnac-Amitai et al., 1990), which has been overlooked in most modeling studies. To capture this variability, we use two separate models for thick-tufted L5PCs (Hay et al., 2011; Almog and Korngreen, 2014) with partly overlapping ion-channel mechanisms and modes of input-output relationships. Furthermore, we generate alternative models that capture a continuum of firing properties between those attained by the models of Hay et al. (2011) and Almog and Korngreen (2014). We use these models to explore the sensitivity of different input-output relationships to subtle variation in ion-channel kinetics or Ca^2+^ dynamics. We show that most of the effects of SCZ-associated variants reported in Mäki-Marttunen et al. (2016) are robust across different types of layer V pyramidal neurons. Further, to generalize the results to *in vivo*-like conditions, we show that the effects of these model variants on single-L5PC excitability and integration of inputs persist when the model neuron is stimulated with noisy inputs. We also show that the model variants alter the way L5PCs code the input information both in terms of output action potentials and intracellular [Ca^2+^], which could contribute to both altered activity in the downstream neurons and synaptic long-term potentiation. Although our models of common variants alone cause small alterations to the neuron behavior, we show that, when combined, they can radically affect the neuronal responses. Taken together, our results show a wide diversity in how SCZ-associated voltage-gated ion channel-encoding genes affect input-output relationships in L5PCs, and our framework helps to predict how these relationships are correlated with each other.

## 2. Materials and Methods

### 2.1. The L5PC Models and Underlying Ion Channels

We employ two neuron models, the “Hay model” (Hay et al., 2011) and the “Almog model” (Almog and Korngreen, 2014). Both models are multi-compartmental Hodgkin-Huxley type of models with reconstructed morphologies from layer V thick-tufted pyramidal neurons. The Hay model includes the following trans-membrane currents: Fast inactivating Na^+^ current (*I*_*Nat*_), persistent Na^+^ current (*I*_*Nap*_), non-specific cation current (*I*_*h*_), muscarinic K^+^ current (*I*_*m*_), slow inactivating K^+^ current (*I*_*Kp*_), fast inactivating K^+^ current (*I*_*Kt*_), fast non-inactivating K^+^ current (*I*_*Kv*3.1_), high-voltage-activated (HVA) Ca^2+^ current (*I*_*CaHVA*_), low-voltage-activated (LVA) Ca^2+^ current (*I*_*CaLVA*_), small-conductance Ca^2+^-activated K^+^ current (*I*_*SK*_), and finally, the passive leak current (*I*_*leak*_). The Almog model includes a slightly different set of trans-membrane currents: Fast inactivating Na^+^ current (*I*_*Nat*_), non-specific cation current (*I*_*h*_), slow inactivating K^+^ current (*I*_*Kp*_), fast inactivating K^+^ current (*I*_*Kt*_), HVA Ca^2+^ current (*I*_*CaHVA*_), medium-voltage-activated Ca^2+^ current (*I*_*CaMVA*_), small-conductance Ca^2+^-activated K^+^ current (*I*_*SK*_), large-conductance voltage and Ca^2+^-gated K^+^ current (*I*_*BK*_), and finally, the passive leak current (*I*_*leak*_).

It is not perfectly clear which particular ion channel subunits underlie each of these currents. mRNAs of ion channel-encoding genes *KCNA2, KCND2, KCND3, CACNA1A, CACNA1B, CACNA1C, CACNA1D, CACNA1E, CACNA1G, CACNA1H, CACNA1I, HCN1*, and *HCN2* were observed in L5PCs in a study of postnatal rat neocortices at various stages of development (Christophe et al., 2005). Of these, *CACNA1A, CACNA1B, CACNA1C*, and *CACNA1D* encode the alpha subunit of HVA Ca^2+^ channels and thus contribute to *I*_*CaHVA*_, and *CACNA1G, CACNA1H, CACNA1I* contribute to *I*_*CaLVA*_ and possibly *I*_*CaMVA*_^1^, while the genes *KCNA2, KCND2*, and *KCND3* encode K^+^ channel subunits that might contribute to the slow *I*_*Kp*_ current, and the genes *HCN1* and *HCN2* encode subunits for a non-specific ion channel (contributing to *I*_*h*_). Expression of *KCNC1* was observed in certain subpopulations of L5PCs (Akemann et al., 2004), and expression of *KCNB1* and *KCNB2* was observed in layer V or VI pyramidal neurons (Guan et al., 2007). Channels encoded by *KCNB1* and *KCNB2* subunits likely contribute to the *I*_*Kp*_ current due to their slow activation kinetics, while *KCNC1*-based channels form the *I*_*Kv*3.1_ current included in the Hay model. Expression of muscarinic potassium channel-encoding genes *KCNQ2* and *KCNQ3* in L5PCs was reported in Battefeld et al. (2014), and these genes are known to contribute to the *I*_*m*_ current.

Expression of Na^+^ channel subunit-encoding genes *SCN2A* and *SCN8A* was observed in pyramidal cells of all layers of human epileptic tissue (Tian et al., 2014). In another study, expression of genes *SCN1A, SCN2A, SCN3A*, and *SCN6A* in L5PCs was observed (Whitaker et al., 2000), while expression of *SCN1A* was not observed in Tian et al. (2014). Of the genes encoding Na^+^ channel α subunits, *SCN1A, SCN2A*, and *SCN3A*, alongside with *SCN9A*, are tetrodotoxin-sensitive (Catterall et al., 2005) and thus form both the transient (*I*_*Nat*_) and persistent (*I*_*Nap*_) Na^+^ currents. Whether these genes contribute to the current *I*_*Nat*_ or *I*_*Nap*_ may depend on the modulatory subunits that they are associated with (Ma et al., 1997).

Expression of *KCNN1* and *KCNN2* has been observed in L5PCs, while expression of the third gene affecting the *I*_*SK*_ current, namely *KCNN3*, is weak throughout the neocortex (although more prominent in pyramidal neurons in hippocampal region CA1) (Ballesteros-Merino et al., 2014). The α subunits of BK channels, encoded by *KCNMA1* and giving rise to the *I*_*BK*_ current in Almog model, were found to be expressed in L5PCs in Benhassine and Berger (2005).

Most of the above genes encode the α subunit of the underlying ion channel. Many ion channels incorporate also modulatory subunits, the presence of which may change the kinetics or voltage-dependence of the ion channel. As an example, the β2 subunit of Ca^2+^ channels, encoded by *CACNB2*, associates with L-type Ca^2+^ channels (where the α pore is encoded by *CACNA1S, CACNA1C, CACNA1D*, or *CACNA1F*).

In addition to describing the dynamics of these trans-membrane currents, the Hay and Almog models also describe the dynamics of the intracellular Ca^2+^ concentration, [Ca^2+^]_*i*_. According to the models, [Ca^2+^]_*i*_ is increased by the currents conducted by Ca^2+^ channels and otherwise decays toward a resting-state level of [Ca^2+^]_*i*_. The extrusion of Ca^2+^ is contributed by many intracellular molecules, but two types of proteins possess a key role, namely, sarco/endoplasmic reticulum Ca^2+^ ATPase (SERCA) and plasma membrane Ca^2+^ ATPase (PMCA). The SERCA proteins pump cytosolic Ca^2+^ into sarcoplasmic or endoplasmic reticulum, which later releases the excessive Ca^2+^ into the cytosol to strengthen the Ca^2+^ transients caused by opening of the voltage-gated Ca^2+^ channels. SERCA pumps are encoded by three genes, namely, *ATP2A1, ATP2A2*, and *ATP2A3*, of which *ATP2A2* is widely expressed in the brain (Baba-Aissa et al., 1998). PMCA proteins pump intracellular Ca^2+^ into the extracellular medium, and are encoded by genes *ATP2B1, ATP2B2, ATP2B3*, and *ATP2B4*, all of which are widely expressed in the brain (Stahl et al., 1992, 1994).

The distribution of the listed channels across the dendritic tree vary in these two models. In the Hay model, the basal dendrites only contain the leak and *I*_*h*_ current, and the initial axon segments only contain the leak current. The soma, by contrast, expresses all current types except for *I*_*m*_, and the apical dendrite expresses all currents except for *I*_*Nap*_, *I*_*Kp*_, and *I*_*Kt*_. The channel conductances are constants across the type of segment (somatic, apical, basal, axonal) except for the Ca^2+^ currents and the *I*_*h*_ current. The *I*_*CaHVA*_ and *I*_*CaLVA*_ currents are piece-wise constant such that the conductances is 10-fold or 100-fold, respectively, in the “hot area” of Ca^2+^ channels, located 685–885 μm from the soma in the apical dendrite. The *I*_*h*_ conductance increases exponentially by distance in the apical dendrite toward the distal end, but it is constant in soma and basal dendrites. In the Almog model, all active currents are location-dependent, such that the maximal conductances of *I*_*Nat*_, *I*_*CaHVA*_, *I*_*CaMVA*_, *I*_*SK*_, and *I*_*BK*_ decrease linearly from soma toward a predefined distance along the apical dendrite and then level off to constant. By contrast, the maximal conductances of *I*_*Kp*_ and *I*_*Kt*_ decrease exponentially from the soma toward the end of the apical dendrite, while that of the *I*_*h*_ current increases as a sigmoid function toward the end of the apical dendrite. All maximal conductances are non-zero constants along the basal dendrites.

Importantly, the two models are also different in terms of the firing behavior. One of the most obvious differences is that the Hay-model neuron fires tonically in response to somatic DC, while the Almog model expresses a rhythmically bursting (“chattering”) firing pattern, where each burst consists of 3–5 spikes. Both types of behavior are typical in the thick-tufted L5PC population, although the chattering firing pattern is less frequently observed (Chagnac-Amitai et al., 1990; Mason and Larkman, 1990; Yang et al., 1996). Both models are capable of describing back-propagating action potential-induced Ca^2+^ spike firing (BAC firing), where a coincidence or near-coincidence of a back-propagating action potential and apical stimulation generates a dendritic Ca^2+^ spike that propagates to soma to induce further spiking. In L5PCs, BAC firing is an important property that is hypothesized to underlie the special role of the cells in coupling feed-forward and feedback information (Larkum, 2013).

The morphologies accompanying the two models also differ from one another. The Almog model morphology is larger than that of the Hay model, both in terms of total dendritic length (13.5 vs. 12.7 mm) and total area (0.059 vs. 0.031 mm^2^), but is described by a smaller number of neurite sections (153 vs. 196).

#### 2.1.1. Altered Hay and Almog Models

**Figure 1:**
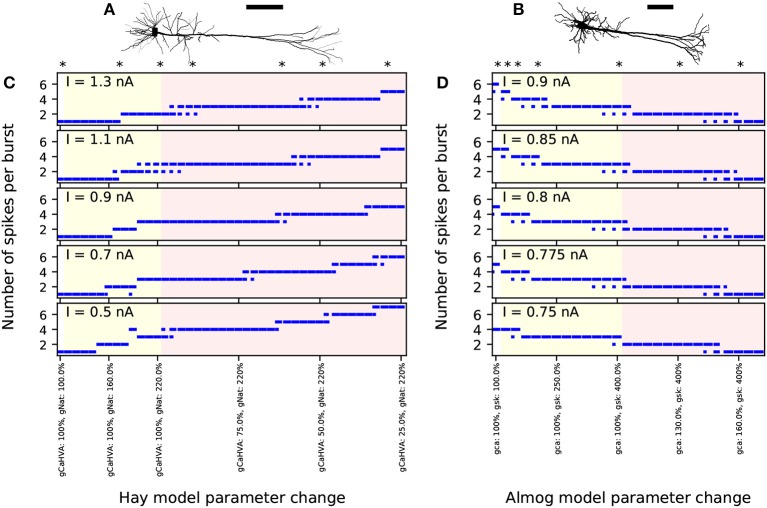
Number of spikes per burst changes when few ion-conductance parameters of the Hay and Almog model are changed. **(A,B)** Morphologies of the Hay model **(A)** and Almog model **(B)** neurons. Scale bar 200 μm; the x-axes point toward superficial cortical layers. **(C,D)** Numbers of spikes in bursts in Hay model **(C)** and Almog model **(D)** and their alterations. The x-axis shows the different altered models. The first entries (white background) correspond to the unaltered Hay or Almog model, while the twelve following entries (yellow background) correspond to altered Hay models where the maximal conductance of *I*_*Nat*_ in the apical dendrite is increased by 10 to 120% **(C)** or Almog models where the maximal conductance of *I*_*SK*_ is increased by 25 to 300% **(D)**. In **(C)**, the rightmost 30 entries (red background) correspond to altered Hay models where the maximal conductance of *I*_*Nat*_ in the apical dendrite is increased by 120% and the maximal conductance of *I*_*CaHVA*_ in the soma is decreased by 2.5 to 75%. In **(D)**, the rightmost 14 entries correspond to altered Almog models where the maximal conductance of *I*_*SK*_ increased by 300% while *I*_*CaHVA*_ and *I*_*CaMVA*_ are increased by 5 to 70%. The models marked with asterisk were chosen for more detailed analysis in the rest of the present work, see Table 1 for the parameter changes corresponding to these models. The y-axis shows the number of spikes per burst in response to a somatic DC. If there were bursts of different number of spikes during the considered 3.5 s period, the length of the horizontal bar reflects the fraction of bursts with the corresponding number of spikes (first two bursts not included due to possible spike-frequency adaptation).

While the Hay-model neuron produces tonic spiking in response to somatic DC, the Almog-model neuron produces rhythmic bursting. To generalize findings from these two models with such different spiking dynamics, we generate intermediate models by changing certain maximal channel conductances in the two models. We present *altered Hay models*, which produce bursting response to a somatic DC, and *altered Almog models*, which produce a smaller number of spikes per burst than the unaltered Almog model or even tonic spiking. We call these models “Hay-A_*i*_” and “Almog-A_*i*_”models, where the index *i* = 1, …, 6 refers to the type and magnitude of the parameter change (see Table 1). Figure 1 shows how the number of spikes per burst, as a response to a somatic DC, is changed in the models by changing two conductance parameters: the maximal conductance of *I*_*Nat*_ in the apical dendrite and the maximal conductance of *I*_*CaHVA*_ in the soma (Hay model), or the maximal conductance of *I*_*SK*_ across the neuron and the maximal conductance of *I*_*CaHVA*_ and *I*_*CaMVA*_ across the neuron (Almog model).

**Table 1:**
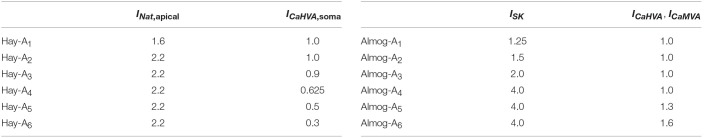
Parameter alteration in the alternative models.

*In Hay models, the parameters controlling the maximal conductance of I_Nat_ in the apical dendrite and that of I_CaHVA_ in the soma are changed, other parameters remain fixed as given in Table 3 of Hay et al. (2011). In Almog models, the parameters controlling the maximal conductances of I_SK_, I_CaHVA_, and I_CaMVA_ are changed, and others remain fixed as given for “Cell 5” in Table 1 of Almog and Korngreen (2014). The table entries show the factor by which the corresponding maximum-conductance parameters are changed (1.0 = “no change”)*.

### 2.2. *In vivo*-Like Synaptic Inputs

We perform some of our simulations in the presence of dense synaptic background firing as expected *in vivo*. We do this following the methods of Hay and Segev (2015): we distribute 10,000 glutamatergic (AMPA and NMDA) and 2,500 GABAergic synapses across the apical and basal dendritic trees. The maximal synaptic conductances are 0.4 nS for glutamatergic and 1.0 nS for GABAergic synapses, and the reversal potentials are 0 and –80 mV, respectively. The AMPA and GABA conductances have dual exponential shapes with rise times 0.3 ms (AMPA) and 1 ms (GABA) and decay times 3 ms (AMPA) and 20 ms (GABA). The NMDA conductance has likewise a dual exponential shape with rise time 2 ms and decay time 65 ms, but it is filtered by a sigmoid function that describes the dependence of the conductance on Mg^2+^ concentration and membrane potential to take into account the Mg^2+^ block in the NMDA receptors (Jahr and Stevens, 1990). The activation times of the synapses follow a Poisson process with rate 0.72 Hz (glutamatergic) or 7.0 Hz (GABAergic). The synapses are short-term depressing with recovery time *D* = 800 ms and single-release consumption rate *U*_*se*_ = 0.6 (glutamatergic) or *U*_*se*_ = 0.25 (GABAergic).

### 2.3. Genes Included in the Study

We study the same set of genes as in our previous work (Mäki-Marttunen et al., 2016). Several of these genes were found to contain single-nucleotide polymorphisms (SNPs) associated with a high risk of SCZ (p-value smaller than 3·10^−8^ in the data of Ripke et al., 2014), namely, *CACNA1C, CACNB2, CACNA1I, ATP2A2*, and *HCN1*. Using a more relaxed threshold (*p*-value smaller than 3·10^−5^), we extended this set by the genes *CACNA1D, SCN1A, KCNB1, KCNMA1* and *ATP2B2*. Compared to our previous study (Mäki-Marttunen et al., 2016), we left out the gene *SCN9A* and *KCNN3* due their low expression in L5PCs, but included *KCNMA1*, which encodes the α subunit for the BK channel. The corresponding current, *I*_*BK*_ was described in the Almog model but not in the Hay model. We also omitted the gene *KCNS3* that was included in Mäki-Marttunen et al. (2016) due to its marginal effects: it encodes a modulatory subunit of the Kv2 channels, which themselves showed small contribution to the firing behavior according to the model of Mäki-Marttunen et al. (2016).

It should be noted that we used the SNPs reported in Ripke et al. (2014) only to identify the above SCZ-related genes, and due to lack of functional genomics data, we could not include the actual physiological effects of the SCZ-related SNPs in our simulation study. Instead, we searched the literature for functional genomic studies reporting the effects of any genetic variants of these genes—in the case of voltage-gated ion channel-encoding genes, these effects typically included altered voltage-dependence and kinetics. Table A1 in Supplementary Material lists all such studies that we found in the literature where the effects of a variant of one of the above genes was measured in a way that could be directly implemented as a parameter change in one or more of our models. Not all of these genes have been shown to be expressed in thick-tufted L5PCs in specific (see above), however, they all show expression in the cortex (Mäki-Marttunen et al., 2018a).

### 2.4. Scaling of Variants

As SCZ is a polygenic disorder, it is likely that the risk of obtaining the disorder is not caused by any of the SCZ-related common sequence variants alone, due to the small effect size for each. However, when sufficiently many of them are present, the combined effect could alter the electrophysiological properties. Although rare variants exist and contribute to the risk of SCZ by ~1–2% (Singh et al., 2017), there are not sequence variants that would be either necessary or sufficient for the emergence of the disease. To take this into account, we used the downscaling approach of Mäki-Marttunen et al. (2016) to model the effects of common SCZ-associated variants in L5PCs. In short, we simulated the neuron model with the parameter changes representing the variants as reported in Table A1 (Supplementary Material). If the variant dramatically altered the neural response, the parameters were brought closer to the control values so that there were no large difference between the firing behaviors of the control neuron and the mutant with the downscaled variant. The downscaling criteria were set so that no variant should alone dramatically alter the threshold amplitudes for chosen stimuli to initiate an action potential nor radically change the steady-state firing behavior of the neuron.

The variants of Table A1 (Supplementary Material) were scaled down to make sure the following four conditions were met:

- The threshold amplitude *A*_1_ for a somatic square-current pulse of 5 ms duration should not change by more than 15%,
- The threshold amplitude *A*_2_ for a distal alpha-shaped synaptic conductance (time constant 5 ms) should not change by more than 15%,
- The threshold amplitudes for a combined stimulus of somatic square-current injection (amplitude *A*_3*a*_) and distal synaptic conductance (time constant 5 ms, max. amplitude *A*_3*b*_, applied 2.5 ms after the somatic pulse) should not change by more than 15%, and
- The integrated difference between the f-I curves of the considered neuron and the control neuron should not be more than 10% of the integral of the control neuron f-I curve.

In case one or more of the conditions I–IV were violated, the change of the model parameters related to the variant were scaled down, all parameters in proportion, to a threshold fraction *c* < 1 of the original effect (the smallest fraction with which the violation was observed). There are no data on how large parameter changes can be expected from common variants, and indeed, the chosen threshold values (±10% and ±15%) were hand-picked as representative values for mild deviations from control behavior. For this reason, we consider variants with different scalings where the threshold effect factor *c* is multiplied with another factor ϵ < 1. In this work, we consider the scaling factor values $\epsilon =\frac{1}{2}$ and $\frac{1}{4}$, and we also display the effects of the corresponding opposite variants $\epsilon =-\frac{1}{2}$ and $-\frac{1}{4}$. For the variants that did not violate the conditions I–IV, we sought for the threshold effect up to twice the original effect (*c* < 2), and if the variant obeyed conditions I–IV for all values *c* < 2, we considered the original variant the $\epsilon =\frac{1}{2}$ variant and applied other scalings with respect to that. The scaling coefficients for the Hay and Almog models are given in Table A2 (Supplementary Material), and Table S2 complements these data with the scaling coefficients for the altered Hay and Almog models. For more details on the variant scaling, see our earlier work (Mäki-Marttunen et al., 2016; Mäki-Marttunen et al., 2017).

Table S1 shows the amplitudes *A*_1_, *A*_2_, *A*_3*a*_, and *A*_3*b*_ for each of the applied models. In the altered Hay models, the BAC firing properties according to which (among others) the unaltered Hay model was fitted in Hay et al. (2011) were radically changed. As a somatic square pulse current could induce a single spike in the unaltered Hay model, suprathreshold somatic currents always induced a burst of two or more spikes in the altered Hay models. These bursts were mediated by the more prominent activation of dendritic Na^+^ and Ca^2+^ channels than in the unaltered Hay model, which is shown by the fact that the dendritic membrane potentials are highly elevated immediately after the first spike unlike in the unaltered Hay model, as shown in Table S1. Such behavior has been reported previously in certain subpopulations of L5PCs (Amitai et al., 1993), although not in the data to which the Hay model and Almog model were fitted. Therefore, in the analysis below we are careful about the results obtained using altered Hay models, especially Hay-A_2_–A_6_, while we consider results from the unaltered Hay model as well as the unaltered and altered Almog models more established.

### 2.5. Simulation Software

We used the NEURON software with Python interface for simulating the model neurons (Hines and Carnevale, 1997). Our simulation scripts are publicly available in ModelDB, entry 249463 (http://modeldb.yale.edu/249463).

## 3. Results

### 3.1. Model Variants Influence Steady-State Firing in All L5PC Models

**Figure 2:**
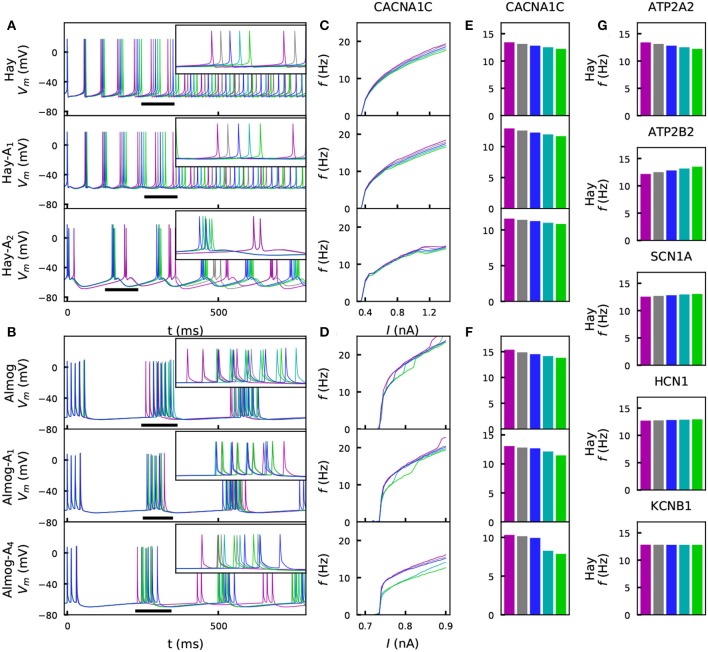
Steady-state firing behavior is affected by the variants. **(A)** Membrane potential time series of the original Hay model (top panel), and of the altered Hay models Hay-A_1_ (middle panel) and Hay-A_2_ (bottom panel). Blue curves show the control neuron behavior, as other colors show the effects of a *CACNA1C* variant (purple: $\epsilon =\frac{1}{2}$, gray: $\epsilon =\frac{1}{4}$, cyan: $\epsilon =-\frac{1}{4}$, green: $\epsilon =-\frac{1}{2}$). Insets show a zoomed-in view on the time marked with horizontal black bars. **(B)** Membrane potential time series of the original Almog model (top panel), and of the altered Almog models Almog-A_1_ (middle panel) and Almog-A_4_ (bottom panel). Blue curves show the control neuron behavior, as other colors show the effects of the same *CACNA1C* variant as in panel **(A)**. **(C, D)**: f-I curves of the *CACNA1C* model variant of panel **(A)** according to the unaltered and altered Hay **(C)** or Almog **(D)** models. Blue curves show the control neuron f-I curve, and other colors show the different scalings of the variant (see **A,B**). **(E,F)** Averages of the f-I curves of unaltered and altered Hay **(E)** and Almog **(F)** models across the somatic current amplitudes [integrated across amplitudes 0.3–1.4 nA **(C)** or 0.65–0.9 nA in **(D)** and divided by 1.1 nA **(C)** or 0.25 nA **(D)**]. **(G)** Averages of the f-I curves of the Hay model with different variants—see the top panel of **(E)** for the corresponding data from the *CACNA1C* variant.

We investigated the steady-state behavior of the different model neurons when a direct current (DC) was applied to the soma. Figures 2A–D shows the time courses and f-I curves (firing frequency as a function of the DC amplitude) for one variant as predicted by different models. Figures 2E,F shows the summary statistics of these data, and Figure 2G shows corresponding Hay-model data for a wider set of representative variants. These variants show notable differences from the corresponding control neuron data [Figure 2E (top panel) and Figure 2G]. However, the differences between the variant and control neuron f-I curves are limited by the explicit constraint posed by the scaling condition IV (see section 2). Although this constraint was only applied to the positively scaled variants ($\epsilon =\frac{1}{2}$ and $\epsilon =\frac{1}{4}$), their opposite variants ($\epsilon =-\frac{1}{2}$ and $\epsilon =-\frac{1}{4}$) mostly show well-constrained f-I curves as well (i.e., their f-I curves are not much further from the control data than those of the positively scaled variants). This indicates that the systems behave in a relatively linear manner with respect to model parameter alterations of this magnitude: for a more rigorous analysis on this (see Figure S1).

Figures 2E,F shows that for the effects of the chosen *CACNA1C* variant were qualitatively same across the models. Figure S2 shows that the same applies to the rest of the six variants of Figure 2G. We confirm this result in Figure S3, where the averages of the f-I curves for all variants of Table A2 (Supplementary Material) are plotted, and in Table S3, which shows the correlations of these quantities (for $\epsilon =\frac{1}{2}$ variants) between the models. Figure S3C illustrates how these correlations are determined. The differences between the predictions of different models are caused by several factors. Firstly, the relationships between the half-activation voltages and slopes of the activation and inactivation curves were different between the Hay and Almog models. Therefore, a variant that simultaneously changes many of these parameters may have qualitatively different effects in the Hay models compared to those in the Almog models, see a discussion on this topic in Mäki-Marttunen et al. (2017). Secondly, in a bursting neuron model, the f-I curve shows steep increases when the number of spikes per burst increases by one, while the non-bursting spiking models have smoother f-I curves– thus, the firing rate of a lower-gain variant may for certain values of the input-current amplitude exceed that of the control neuron (see, e.g., the negatively scaled *SCN1A* variants for the Almog-model neuron in Figure S2B).

We confirmed our results also using the Hay model with spontaneously activated excitatory and inhibitory synapses (as in Hay and Segev, 2015). Figure S4A shows the membrane potential traces of control and a *CACNA1C* variant and Figure S4B shows f-I curves corresponding to the variants of Figure 2 when this *in vivo*-like noisy input was applied to the Hay-model neuron. Figure S4C shows the collection of data from all variants (corresponding to the data of Figure S3) in the noisy Hay-model neuron. The variant effects on noisy f-I responses (Figures S4B,C) were qualitatively similar to those in the absence of noise (Figures S2A, S3A). We also analyzed the effects of decreased NMDA and GABA receptor conductances on the Hay-model neuron firing under spontaneously activated synapses (Figure S4D). Importantly, the effects of the model variants of voltage-gated ion-channel and Ca^2+^ transporter-encoding genes were different from the impacts of altered synaptic inputs: while many model variants affected the L5PC gain (Figure S4B), alterations in synaptic conductances made the neuron fire with approximately the same increment or decline in firing rate across the tested perisomatic current amplitudes (Figure S4D). Decreasing the NMDA and GABA receptor conductances simultaneously did not appear to change the firing behavior of the L5PC, but kept the steady-state firing close to that observed in the control case (Figure S4D, right panels). Taken together, these results suggest that the steady-state firing of L5PCs is robustly (in a consistent manner across many neuron models, and also in presence of noise) affected by variants of SCZ-associated voltage-gated ion channel and Ca^2+^-transporter encoding genes, and that these effects are dissimilar to those caused by synaptic scaling.

### 3.2. Model Variants Modify the Interaction of the Perisomatic Region and Apical Dendrite

Cortical neurons *in vivo* express a bimodal activity pattern that oscillates between a regime of high (“up” state) and low (“down” state) spiking activity in a slow (<1 Hz) frequency (Steriade et al., 1993). These states are prominent during anesthesia and sleep (Destexhe et al., 2007), but occur also in unanesthetized animals during quiet wakefulness (Petersen et al., 2003; Luczak et al., 2007). Most studies on up and down states apply electrophysiological measurements, but they have also been observed in Ca^2+^ imaging studies (see, e.g., Kerr et al., 2005; Grienberger et al., 2012). The origins and detailed characteristics of the up and down states are disputed, but it is known that L5PCs play an important role in the generation or maintenance of these states (Neske, 2016). The increased excitability in the up vs. down state could be contributed by at least three mechanisms: (1) intrinsic excitability (modulation of ion channels), (2) coordination of synaptic inputs, and (3) ephaptic coupling (Wilson, 2008; Anastassiou et al., 2011). In Hay et al. (2011), a simplistic model of the up state, where the proximal apical dendrite was depolarized with a long sub-threshold square-pulse stimulus, was presented, while the resting state of the neuron was considered the down state. This simplistic model of the up state could represent the effects of any of the above three mechanisms. In our previous study (Mäki-Marttunen et al., 2016), we showed by following this simplistic description of up and down states that SNP-like model variants of SCZ-associated genes affected the properties of integration of somatic and apical stimuli in both regimes. Here, we extended these results by considering a wider set of L5PC models and stimulation protocols, as well as a more *in-vivo*-compatible representation of the up and down states.

Following Hay et al. (2011) and Mäki-Marttunen et al. (2016), we implemented the simplistic up-state model such that the apical dendrite (at a distance of 200 μm from the soma) was stimulated with a 600-ms square pulse current (200 ms in Hay et al., 2011; Mäki-Marttunen et al., 2016) whose amplitude was 85% (95.5% in Hay et al., 2011) of the threshold current for inducing a somatic spike. In the middle of this stimulus, a 5-ms square pulse current was injected at the soma with an amplitude 40% (37.9% in Hay et al., 2011) of the threshold amplitude for eliciting a spike. Preceding or following the onset of this short pulse, an EPSP-like current was injected at the apical dendrite at a distance of 600 or 850 μm (700 μm in Hay et al., 2011) from the soma. The rise and decay time constants of this EPSP-like current were 0.5 and 5 ms, respectively, and its maximal amplitude was 50 or 15% (44.0% in Hay et al., 2011) of that needed for eliciting a somatic action potential. In the down state, the long square pulse current was absent, and to compensate for this, the somatic stimulus was suprathreshold with an amplitude 135% of the threshold amplitude for eliciting a spike (the exact value not given in Hay et al., 2011, but in Mäki-Marttunen et al., 2016 similar responses were obtained with an amplitude 136.4% of the threshold amplitude). The relative values were used in order to transfer the protocol from one model (the Hay model) to the others (the Almog model and the altered Hay and Almog models): the absolute values of the amplitudes are given in Table S4.

**Figure 3:**
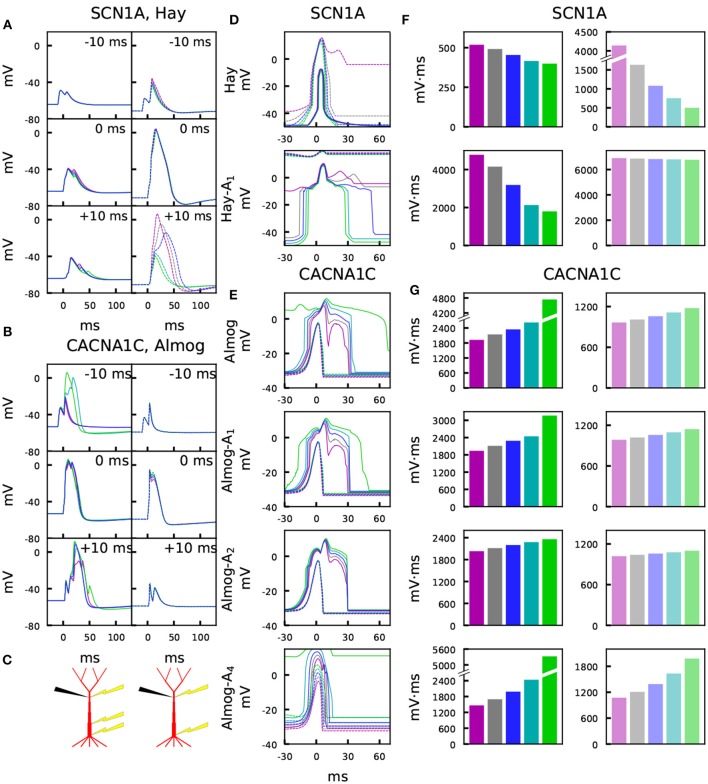
Variants affect the sensitivity to paired somatic and apical stimuli during up and down states. **(A,B)** Membrane potential time courses at the apical dendrite 700 μm from the soma in Hay **(A)** and Almog **(B)** models. Solid curves (left) represent the up-state responses, and the dashed curves (right) represent the down-state responses. The somatic 5-ms stimulation is activated at *t* = 0 ms, while the apical EPSP-like current is activated at –10 ms (top), 0 ms (middle), or +10 ms (bottom). Blue: control, other colors: different scalings of the *SCN1A* **(A)** or *CACNA1C* **(B)** variants of Figure 2. **(C)** Schematic illustration of the recording (black) and stimulation (yellow) protocols in the up-state (left) and down-state (right) paradigms. **(D,E)** Up- and down-state temporal windows representing the sensitivity of the Hay **(C)** and Almog **(D)** model neurons to a combination of somatic (onset at 0 ms) and apical (onset at the value of the x-axis) stimuli. Blue: control neuron, other colors: different scalings of the *SCN1A* **(C)** or *CACNA1C* **(D)** variants of Figure 2. The x-axis shows the inter-stimulus interval (ISI), and the y-axis shows the maximum (across time) membrane potential at the apical dendrite 700 μm from the soma. Solid curves represent the up-state responses, and the dashed curves represent the down-state responses. **(F,G)** Integrals of the up- (left panels, strong colors) and down-state (right panels, dim colors) temporal windows of panels **(C)** and **(D)** across the shown ISIs (–30 to 70 ms) for the *SCN1A* variant according to the Hay models **(F)** and for the *CACNA1C* variant according to the Almog models. In the calculation of the integrals, the baseline amplitude –50 mV **(F)** or –40 mV **(G)** was subtracted from the data.

Figure 3 illustrates the temporal sensitivities of the L5PC models to combined somatic and apical stimuli, both when the neuron was in an up or down state (up state modeled simplistically, see above). These temporal sensitivity windows were different for different neuron models. In both up- and down-state protocols, coincident stimuli tended to produce a larger dendritic voltage response than non-coincident stimuli (Figures 3A,B). Consistent with the fact that the bursting models had smaller Ca^2+^ channel conductivities (which caused smaller SK currents) and (in Hay model) larger Na^+^ channel conductivities at the apical dendrite, the bursting models (Figures 3D–E, Hay-A_1_, Almog) had a wider sensitivity window, i.e. they generated large responses even when the inputs were temporally more apart, than the non-bursting and mildly bursting models (Figures 3D–E, Hay, Almog-A_4_). In Figures 3F,G, we quantified the sensitivity of the L5PC to coincidence of apical and perisomatic inputs by integrals of the sensitivity windows of Figures 3D–E for one variant (*SCN1A* variant for Hay models, *CACNA1C* variant for Almog models), and the corresponding data for all variants of Figure 2 are shown in Figure S5. Figure 3 shows that the variants affected both the sensitivity of the coincidence detection as well as the amplitudes of the dendritic response to the combined stimuli. As expected, the variant effects were typically more constrained in the neuron models that expressed little or no bursting behavior (Hay model, top panel of Figure 3F; Almog-A_5_ model, bottom panel of Figure 3G) than in the other neuron models, where the temporal sensitivity windows were wider. The altered Hay models generally produced a burst of action potentials given the somatic stimulation alone (see Figure 1), which back-propagated to the dendrites and induced a Ca^2+^ spike. This is reflected as a lack of temporal window for the Hay-A_1_ model in the down-state protocol (bottom panel of Figure 3D, dashed) and thus modest effects of the variants (bottom panel of Figure 3F, dim): a proper temporal window was also absent for both up- and down-state protocols in Hay-A_2_ to Hay-A_6_ models (data not shown). Extensive simulations of all variants of Table A2 (Supplementary Material) showed that the effects of the variants on the integrals of the temporal windows of the up states (Figures 3F,G, Figure S5) were (moderately) negatively correlated with the effects on the steady-state firing frequency of Figure S3 (correlation coefficient was between –0.3 and –0.7 in the unaltered and altered Hay and Almog models when considering all variants, and between –0.62 and –0.91 when considering only variants of Ca^2+^ channel and Ca^2+^ transporter-encoding genes; Table 3A). Similar results were obtained for the down states, where the correlations were weaker in Hay models but stronger in Almog models compared to the data of Table 3A (not shown).

Furthermore, we also studied the second proposed up- vs. down-state mechanism in particular by considering a scenario where the L5PC received noisy synaptic inputs, and the distinction between the up and down states was determined by the rate of inputs to the synapses. The modification of the excitatory synaptic drive is a more likely explanation for the difference in excitability between the up and down states than that of the inhibitory drive (Wilson, 2008). Therefore, we modeled the down state as a state where the frequency of inputs to the excitatory synapses was decreased by 30% (*f* = 0.504 Hz) from the average estimated value *in vivo* (see Hay and Segev, 2015), while in the up state the frequency of inputs to the excitatory synapses was increased by 10% (*f* = 0.792 Hz). These frequencies of synaptic activation led to firing rates 0.06 and 4.9 Hz in the down and up states, respectively, when no other stimuli were applied. The up-state firing frequency is in line with the experimentally observed values, as a range from 1.1 to 8.2 Hz with a mean of 3.5 Hz was observed in Neske and Connors (2016). For the down state, no systematic estimates have been reported, but the firing frequency has been observed to be extremely low compared to the up-state firing frequency, yet non-zero (Sanchez-Vives and McCormick, 2000; Lőrincz et al., 2015).

**Figure 4:**
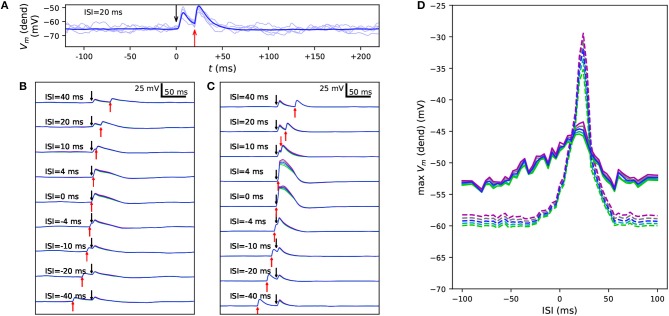
Variants alter the sensitivity to a combination of somatic and apical stimuli during *in vivo*-like noisy up and down states. **(A)** Membrane potential time series at the apical dendrite 700 μm from the soma, when the soma is stimulated at 0 ms and the apical dendrite at +20 ms. Thin, dim curves show the same simulation with different random number seeds, and the thick curve shows the mean of 125 repetitions. **(B,C)** The mean data of panel **(A)** reproduced with different ISIs during a noisy up **(B)** or down **(C)** state. Blue: control, other colors: downscaled versions of a *SCN1A* variant as in Figures 2, 3. **(D)** An overview of the up- (solid) and down-state (dashed) data of panels **(B)** and **(C)**, respectively, as a function of ISI. The y-axis shows the mean apical dendritic membrane potential during a 20-ms interval starting at the onset of the somatic stimulus (0 ms), averaged over 125 repetitions as in **(B)** and **(C)**.

Figure 4 confirms the results of Figure 3 for control neuron and an *SCN1A* variant using an *in vivo*-like background synaptic firing and the description of up and down states as states with increased or decreased (respectively) rates of random synaptic inputs. The properties of the somatic current and the apical EPSP-like current were the same as in the experiment of Figure 3, except for the amplitude of the apical EPSP-like current in the up state, which was set 25% of the threshold amplitude to constrain the response. These simulations were performed only for the Hay model, and to show consistent trends, 125 repetitions of each simulation with different random number seeds (affecting the timing of presynaptic spikes) were run. Unlike in the noiseless Hay model but consistent with the Almog-model predictions, these results from the noisy Hay model show a wider temporal window in the up-state protocol than in the down-state protocol (Figure 4D). In both noisy and noiseless cases, the Hay model shows higher amplitudes in down-state protocols where the presynaptic spiking was sparser (noisy, Figure 4D) or the proximal dendritic depolarization was missing (noiseless, Figures 3A,D) but where the somatic stimulus was greater than in the up-state protocol. By contrast, in the Almog model and all altered Almog models, the down-state amplitudes were always lower than the up-state amplitudes (Figures 3B,E), despite an even larger compensation of the somatic stimulus amplitude (in the Hay model, the amplitude is 2.8 times larger in the down-state than in the up-state protocol, and in the Almog models, 3.4 times larger). These observations reflect the differences in the Ca^2+^-activated K^+^ currents between the two models, as discussed in Mäki-Marttunen et al. (2017). Importantly, the effects of the *SCN1A* variant were qualitatively the same in the noisy case (Figure 4) as in the noiseless case (Figures 3A,D)

Taken together, these data suggest that SCZ-associated variants can cause robust alterations of the communication between perisomatic and apical dendritic regions during both up- and down states of the L5PCs. These alterations include both amplification of the dendritic response to coincident stimuli at apical dendrite and the perisomatic region as well as widening of the temporal window of the inter-stimulus interval in which a large dendritic response is induced.

### 3.3. Variants Affect the Adaptation by a Prepulse in an L5PC

**Figure 5:**
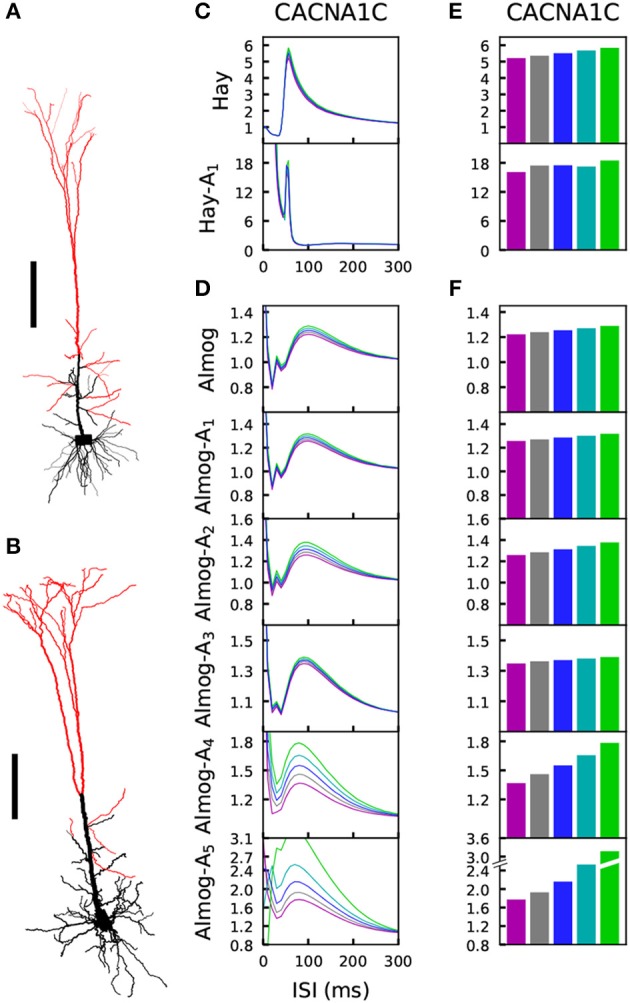
The L5PC adaptation by a prepulse is altered by variants of HVA Ca^2+^ channels. **(A,B)** Illustration of the morphologies of the two models. The model neurons of Hay **(A)** and Almog **(B)** were given stimulation through 3,000 conductance-based synapses that were uniformly distributed along the apical dendrites, except for the most proximal compartments whose distance from the soma were less than 300 **(A)** or 450 **(B)** μm. Scale bar 200 μm. **(C,D)** The synapses of the Hay **(C)** or Almog **(D)** model neuron were activated two times, first at the time instant *t* = 0 with an alpha-shaped (time constant 5 ms) conductance whose maximal amplitude was 10% higher than that needed for inducing a spike, and later after an ISI displayed on the x-axis. The y-axis shows the threshold conductance factor with which the neuron fired an additional action potential—thus, at the limit of large ISI the curves converge to 1, which represents the threshold conductance at rest. The blue curve represents the control neuron, while the other colors represent the data corresponding to the different downscaled versions of the *CACNA1C* variant of Figure 2. **(E,F)** The maximal threshold conductance factor from the curves of **(C)** and **(D)**, excluding the first 40 ms. Colors as in Figure 2.

The medium/slow afterhyperpolarization current in the L5PC, mostly mediated by the Ca^2+^-activated K^+^ currents in L5PCs (Poirazi et al., 2003; Stocker, 2004), contributes to the L5PC adaptation and is thus an important regulator of the neuron firing behavior within a time scale of tens to hundreds of milliseconds following the spike (or other event that leads to large cytosolic Ca^2+^ transients). This time scale matches exactly the time scale of the PPI of the startle, which is impaired in SCZ patients (Turetsky et al., 2009). L5PCs are not a fundamental part of the mammalian startle network, but the insights gained from the biophysically detailed L5PC models can be used to make hypotheses on the cellular mechanisms in neurons within the startle network, such as the giant pontine reticular formation neurons (cf. Mäki-Marttunen et al., 2018a). In a similar manner as in Mäki-Marttunen et al. (2016), we studied how the variants of voltage-gated ion-channels and Ca^2+^ transporters affect the L5PC adaptation. To do this, we distributed 3,000 excitatory conductance-based alpha synapses (τ = 5 ms, *E*_*rev*_ = 0 mV) across the apical dendrite of the model L5PC (Figures 5A,B). We used the bisection method to find the threshold conductance *g*_*th*_ for making the neuron fire in response to simultaneous activation of these synapses. We then activated the synapses at time *t* = 0 with a suprathreshold stimulus (amplitude 10% above *g*_*th*_) and again at time *t* = *t*_*ISI*_ with an amplitude *c* × *g*_*th*_. Depending on the interstimulus interval *t*_*ISI*_, the threshold factor *c* for eliciting an additional spike was smaller or larger than 1, reflecting a prepulse facilitatory or inhibitory effect, respectively. Figures 5C,D shows the threshold factors *c* as a function of the inter-stimulus interval ISI for different scalings of a *CACNA1C* variant according to the Hay model, Almog model, and altered Almog models. In Figures 5E,F, the maximal threshold factors *c* (across ISIs larger than 40 ms) are quantified for the *CACNA1C* variant of Figure 2, and Figure S6 shows the corresponding data for the rest of the variants of Figure 2.

Comparison of the variant effects on steady-state firing (Figure 2 and Figure S2) and prepulse-mediated adaptation (Figure 5 and Figure S6) indicates that the variants that increased the steady state firing decreased the adaptation. We confirmed this trend by simulating the effects of all variants of Table A2 (Supplementary Material) on these phenotypes. We found that the effects of the variants on maxima of the prepulse-inhibition curves of Figure 5 were (mostly) negatively correlated with the effects on the steady-state firing frequency of Figure S3 (correlation coefficient was –0.72 in the Hay model, 0.11 in Hay-A_1_ model, and between –0.63 and –0.91 in the unaltered and altered Almog models; Table 3B).

As reported in Mäki-Marttunen et al. (2016), the Hay-model neurons express a relatively strong adaptation current, and the variants have a modest but observable effect on the amplitude of the prepulse-mediated adaptation measured as the firing threshold of a stimulus following a suprathreshold stimuls (Figure 5E and Figure S6A). This prepulse-mediated adaptation is mostly contributed by the SK current (cf. Mäki-Marttunen et al., 2018a), which is revealed by the comparison of the amplitude and kinetics of different potassium currents in the soma and the stimulated dendrite (Figures S7A,B): only SK current has the required strength and long decay time needed to cause such an inhibition. Here, we found qualitatively similar results for the Almog model neurons and altered Almog model neurons (Figure 5F and Figure S6B). In the Almog-model neuron, the dendritic SK currents are relatively large and long-lasting (Figure S7C), while the somatic SK currents are small (Figure S7D). In the altered Almog models, the magnitudes of the dendritic as well as somatic SK currents are increased (Figures S7E–J). This may explain why both the magnitude of the prepulse-mediated adaptation and the effects of the Ca^2+^ channel variants therein are larger in the altered Almog models than in the original Almog model (Figures 5E,F).

Taken together, our results suggest that the magnitude of an SK current-mediated adaptation by a prepulse stimulus can be crucially altered by SCZ-associated gene variants in L5PCs and that these predicted effects are qualitatively similar in many different L5PC models.

### 3.4. Neural Coding Capacity in an L5PC May Be Altered by the Variants

The analyses above shed light on how the neuron responses to stimuli of different types are altered by the SCZ-associated variants. Here, we aim at deeper characterization on how the SCZ-associated genes control the neural response to different combinations of spatially distributed stimuli. We divided the apical dendrite into six regions (0–200, 200–400, 400–600, 600–800, 800–1,000, and > 1,000 μm from the soma) and considered the basal dendrites as the seventh region. 1,000 excitatory conductance-based alpha synapses were uniformly distributed to each region, and as above, the threshold conductance for inducing a spike in the Hay model was determined (Table 2A). We then considered all possible binary combinations of input patterns, where each region is either non-stimulated (all synaptic conductances were set to zero) or simultaneously stimulated (all synaptic conductances were set to half of the threshold conductance). We determined the neuron output for each of these input combinations by considering seven different discrete measures: whether the neuron spiked (number of spikes induced; here 0–3) and whether the Ca^2+^ concentration in the middle of each of the six apical regions remained below 0.00011 mM (0), was between 0.00011 and 0.00012 mM (1), or above 0.00012 mM (2). These threshold values were chosen as approximate classifiers between low, medium, and high activation of intracellular Ca^2+^ signaling; depending on the outcome, the synapses in the region could be depressed (medium [Ca^2+^]) or potentiated (high [Ca^2+^]) as a consequence of synaptic inputs (Artola and Singer, 1993). Determining detailed, physiologically realistic values for these thresholds is out of the scope of this paper—instead, we here aim to show that the SCZ-associated variants may affect the neural coding both in terms of immediate outcome (number of spikes) and long-term, dendritic Ca^2+^-dependent effects (number of [Ca^2+^] thresholds crossed).

**Table 2:** Properties of the Hay-model neuron given the inputs used in the neural coding experiments.

**Region**	**Threshold**						
**A**							
1: 0–200 μm	1.94e-05						
2: 200–400 μm	2.42e-05						
3: 400–600 μm	8.29e-05						
4: 600–800 μm	4.87e-05						
5: 800–1000 μm	6.85e-05						
6: > 1000 μm	0.000138						
7: basal	1.62e-05						
**Input location**	**N**_***spikes***_	**Ca1**	**Ca2**	**Ca3**	**Ca4**	**Ca5**	**Ca6**
**B**							
apic1	0.41	0.04	0.35	0.27	0.34	0.05	0.05
apic2	0.28	0.04	0.35	0.29	0.34	0.05	0.05
apic3	0.28	0.04	0.35	0.59	0.53	0.05	0.05
apic4	0.18	0.36	0.24	0.27	0.16	0.35	0.35
apic5	0.18	0.36	0.20	0.27	0.18	0.35	0.35
apic6	0.18	0.36	0.16	0.27	0.18	0.35	0.35
basal	0.43	0.01	0.20	0.14	0.32	0.00	0.00

***(A)** Threshold conductances with which 1,000 excitatory conductance-based alpha synapses (τ = 5 ms, E_rev_ = 0 mV) induced a spike in the Hay model neuron. The synapses were uniformly distributed across compartments in one of seven regions (1: 0–200 μm, 2: 200–400 μm, 3: 400–600 μm, 4: 600–800 μm, 5: 800–1,000 μm, 6: >1,000 μm from the soma along the apical dendrite, or 7: in the basal dendrites). **(B)** Correlations between the input and output patterns in the control Hay model neuron. The first column shows the correlation coefficient between the number of induced spikes and the presence of the input in the seven input regions, and the other six columns show the correlation coefficients between the [Ca^2+^] output in the six apical regions and the presence of the input in the seven input regions*.

**Figure 6:**
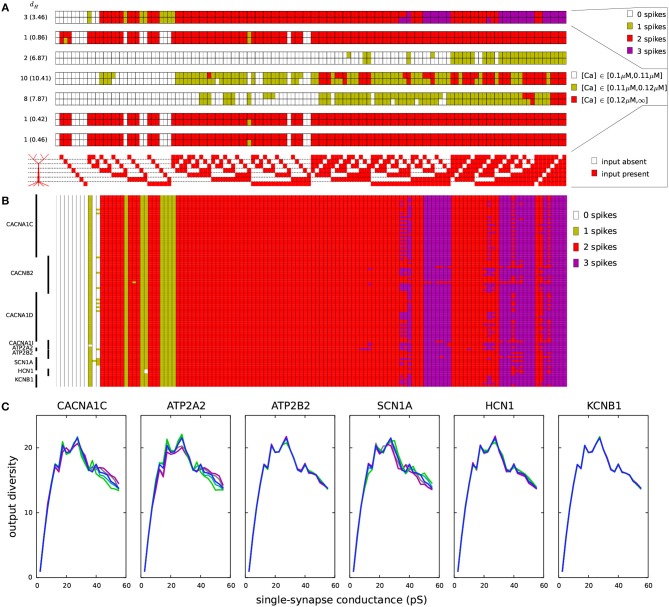
Variants affect the coding of information in L5PCs. One thousand synapses were distributed to each of the regions listed in Table 2A. Different input combinations (128 in total) where synapses in these seven regions were either silent or simultaneously activated were used as inputs. The output of the Hay-model neuron was determined based on the number of action potentials generated (0–3) and the level of intracellular Ca^2+^ concentration (low, medium, or large). **(A)** The maximal conductance of each synaptic input (when activated) was half of threshold value in the underlying dendritic region for inducing a spike (Table 2A). The seven panels show the numbers of spikes induced (top) and the Ca^2+^ response in the regions 1–6 (six panels below) for each of the 128 input combinations. Upper rows of these panels show the control L5PC data, and the lower rows show the data from the *CACNA1C* variant of Figure 2. On the left-hand side of each panel, the Hamming distance between these data are shown (the average Hamming distance across all variants is in the parentheses). The *CACNA1C* variant affected the output of the neuron in terms of both numbers of spikes (the variant caused more spikes to be induced) and the Ca^2+^ response (the variant reduced the [Ca^2+^]) for certain input combinations. Below the panels, the 128 input combinations corresponding to the shown outputs are illustrated—the combinations with fewest active inputs are on the left-hand side and those with the highest numbers of active inputs are on the right. **(B)** Compiled spike-number data from all variants of Table A2 (Supplementary Material). The L5PC is activated as in panel **(A)**. **(C)** Output diversities of the variants of Figure 2. The input combinations were the same as in panels **(A)** and **(B)**, but the maximal conductance of each synapse was fixed (shown on the x-axis). The y-axis shows the output diversity, i.e., number of unique output patterns among the 128 output patterns, for the given conductance.

We first analyzed the input-output relationships in the control Hay-model neuron. The upper row of each panel in Figure 6A shows the discretized response of the control Hay-model neuron in terms of these seven discrete measures for all 128 combinations of stimuli. As expected, all output measures were positively (mostly weakly) correlated with the presence of the corresponding input (Table 2B). The number of output spikes was most correlated (correlation coefficient 0.43 in the control neuron) with the presence of basal stimuli, but it was moderately correlated (correlation coefficient 0.41 in the control neuron) also with the presence of the input stimuli in the apical tuft. The Ca^2+^ output at each apical dendritic region was typically most correlated with the presence of the stimulus at the same region, except for the most proximal apical dendritic region (0–200 μm), which was most correlated with the inputs in three most distal regions (correlation coefficient 0.36), and the fourth apical dendritic region (600–800 μm), which was most correlated with the presence of inputs in the neighboring third region (400–600 μm; correlation coefficient 0.53). The strong correlation between the [Ca^2+^] in the region 600–800 μm from the soma and the presence of inputs in the region 400–600 μm from the soma could be explained by the “hot zone” of Ca^2+^ channels which spans these two regions.

We next analyzed the effects of the SCZ-associated variants on these input-output relationships. The bottom row of each panel in Figure 6A shows the discretized response of the $\epsilon =\frac{1}{2}$ *CACNA1C* variant of Figure 2, and the Hamming distances between the control and variant output patterns (i.e., the number of disagreeing data) are displayed at the left-hand side of each panel (average Hamming distance across all $\epsilon =\frac{1}{2}$ variants shown in parentheses). The input-output correlations were qualitatively the same for the control neuron (Table 2B) and for the *CACNA1C* variant neuron (data not shown). Figure 6B shows the number of spikes (as in the upmost panel of Figure 6A) across all $\epsilon =\frac{1}{2}$ variants. These data show that the variants had largest effect on integration of inputs in the basal dendrites and the second apical compartment (200–400 μm): Out of 32 input patterns where inputs were present in these regions, 11 caused substantial variation (i.e., more than five variants showed a differing output) in the output number of spikes. The corresponding numbers for other pairs of compartments were between 0 and 7. These numbers were, however, highly dependent on the differential scaling of the synaptic inputs at separate locations (see Table 2B): When we varied the scaling coefficient of the synaptic inputs from 0.1 to 1.0 (in Figures 6A,B, 0.5 was used), the relevance of different dendritic compartments varied as well (data not shown).

We next addressed the effects of the SCZ-associated variants on the *output diversity* of the Hay-model neuron. We defined the output diversity as the number of unique output patterns across the given input patterns. This is also a measure of the *coding capacity* of the neuron as it reflects the number of unique ways in which the neuron may respond to (and thus code) the inputs. As an example, although the control neuron in Figure 6A was given 128 different input patterns, it only produced 18 unique output patterns, as, e.g., the pattern where 2 spikes, high [Ca^2+^] in the 1st, 5th, and 6th apical compartments, a medium [Ca^2+^] in the 3rd compartment, and low [Ca^2+^] in the 2nd and 4th compartment were induced was obtained for 27 different input patterns. We calculated the output diversity for the variants of Figure 2 using absolute synaptic conductances ranging from 0.0025 to 0.5 nS—i.e., unlike in Figures 6A,B where synaptic conductance at each compartment was relative to the threshold conductance, here each compartment that received inputs had an equal synaptic conductance. This range of synaptic conductances spanned the discretized space of each output measure, meaning that for each output measure (number of spikes or [Ca^2+^] at a given compartment of the apical dendrite) there were combinations of inputs that produced all values of the measure for the control neuron. To smoothen the data, the output diversity was averaged over eleven repetitions, where a small depolarizing or hyperpolarizing somatic stimulus (whose amplitude ranged from –0.1 to 0.1 nA in steps of 0.02 nA) was applied.

Figure 6C shows the average output diversity as the function of the synaptic conductance. The curves show an expected shape, where the output diversity is close to zero for small input conductances (all combinations produce the same output, namely, no spikes and no significant elevations of [Ca^2+^]), increases for intermediate inputs, and decreases again for large input conductances (where many combinations produce a large number of spikes and large elevations of [Ca^2+^]). Nevertheless, the variants of Figure 2 alter the shape of this curve. The *CACNA1C* and *ATP2A2* variants ($\epsilon =\frac{1}{2}$ scaling) that increased the firing rate in Figure 2 also increased the output diversity for the large synaptic conductances in Figure 6C, and likewise, the *SCN1A* variant that decreased the firing in Figure 2 also decreased the output diversity in Figure 6C. However, the *ATP2B2* variant increased the firing rate (Figure 2) but had little effect on output diversity (Figure 6C). We confirmed the relationship between the integral of the f-I curve and the average output diversity by calculating the correlation between these measures across all variants (Table 3C). We considered the output diversity below (synaptic conductances 2.5 to 32.5 pS) and beyond (synaptic conductances 35 to 50 pS) the approximate peak of the output diversity to separately take into account the neuron responsiveness to small and large-amplitude synaptic stimuli. The variant effects on f-I curve integral were strongly correlated with the effects on output diversity at large synaptic drive (correlation coefficient 0.73), but only mildly correlated with those on output diversity at small synaptic drive (correlation coefficient 0.31). Our earlier results showed that due to the strength of the SK currents, the variants of Ca^2+^-channel and transporter-encoding genes had qualitatively different effects than those of Na^+^ and HCN-channel-encoding genes (Mäki-Marttunen et al., 2017). We therefore also calculated the correlation coefficients separately for Ca^2+^-associated non-Ca^2+^-associated variants. These data showed that f-I curve integrals were moderately correlated with the output diversity at both small (correlation coefficient 0.64) and large (correlation coefficient 0.68) synaptic drives among the Na^+^, HCN, and K^+^-channel encoding variants. Taken together, these data suggest that variants that increase the excitability also increase the diversity of the neuron output: in other words, they make the neuron respond in a wider variety of ways (in terms of downstream spiking activity and modes of plasticity) to a given set of inputs.

**Table 3:** Correlations between different phenotypes across neuron models.

	**Hay**	**Hay-A_**1**_**	**Almog**	**Almog-A_**1**_**	**Almog-A_**2**_**	**Almog-A_**3**_**	**Almog-A_**4**_**	**Almog-A_**5**_**
**(A): CORRELATION OF F-I CURVE AND COINCIDENCE DETECTION DURING UP STATE**								
All:	−0.30	−0.61	−0.70	−0.61	−0.63	−0.66	−0.65	−0.59
Ca^2+^ genes:	−0.62	−0.73	−0.75	−0.69	−0.91	−0.74	−0.85	−0.85
non-Ca^2+^ genes:	−0.12	−0.57	0.88	0.91	0.84	0.89	0.64	0.67
**(B): CORRELATION OF F-I CURVE AND PREPULSE-MEDIATED ADAPTATION**								
All:	−0.72	0.11	−0.91	−0.85	−0.71	−0.81	−0.74	−0.63
Ca^2+^ genes:	−0.74	0.17	−0.95	−0.93	−0.94	−0.90	−0.92	−0.89
non-Ca^2+^ genes:	−0.51	−0.53	0.58	0.51	0.61	0.53	0.48	0.54
	**Hay (small** ***g*)**		**Hay (large** ***g*)**		**Almog (small** ***g*)**		**Almog (large** ***g*)**	
**(C): CORRELATION OF F-I CURVE AND CODING CAPACITY**								
All:	0.31		0.73		0.83		0.75	
Ca^2+^ genes:	0.31		0.74		0.86		0.80	
non-Ca^2+^ genes:	0.64		0.68		−0.28		−0.30	
**(D): CORRELATION OF PREPULSE-MEDIATED ADAPTATION AND CODING CAPACITY**								
All:	−0.38		−0.86		−0.92		−0.86	
Ca^2+^ genes:	−0.38		−0.86		−0.93		−0.90	
non-Ca^2+^ genes:	−0.48		−0.93		−0.36		−0.51	

***(A)** Correlation coefficients between f-I curve integrals (from Figure 2 and Figure S3) and integrals of the temporal windows of apical and perisomatic coincidence detection (from Figures 3F,G) in the up-state protocol according to different Hay and Almog models. Similar but mildly weaker correlations were obtained if the lengths of the temporal window (where dendritic membrane potential exceeded –20 mV) were used instead of the integrals of the temporal windows of Figure 3 (data not shown). **(B)** Correlation coefficients between f-I curve integrals and adaptation curve maxima (from Figure 5) in different Hay and Almog models. **(C)** Correlation coefficients between f-I curve integrals and neuron output diversities in Hay and Almog models. **(D)** Correlation coefficients between adaptation curve maxima and integrals of neuron output diverisity curves (from Figure 6C) in Hay and Almog models. In **(C)** and **(D)**, the correlations with predicted neuron output diversities are separated into two groups, where the average correlation coefficient is calculated as an average for conductances 32.5 pS or smaller (second and fourth columns), or 35 pS or larger (third and fifth columns). In each panel, the first row shows the correlation coefficients across all variants, while the second row shows those across variants in Ca^2+^-related genes (CACNA1C, CACNB2, CACNA1D, CACNA1I, ATP2A2, and ATP2B2) and the third row those across other genes (SCN1A, HCN1, and KCNB1)—note that we excluded KCNMA1 variants from this analysis as the underlying channels were only present in the Almog models*.

We replicated the results of Table 2 and Figure 6 using the Almog model in Table S5 and Figure S8. The results are qualitatively the same, although the relative importance of different sections is different in the Almog model, and the neuron output was on average more sensitive to the effects of the model variants. In the same way as in the Hay model data, the intracellular Ca^2+^ transients were higher in the middle and distal parts of the apical dendrite than in the proximal apical dendrite, except for the most proximal part (0–200 μm), which had large Ca^2+^ transients in Hay model but extremely small in Almog model (Figure 6A and Figure S8A). In addition, both models predicted that the variations in the size of the Ca^2+^ transients caused by the SCZ-associated genes were on average larger in the middle of the apical dendrite than in the proximal and distal apical dendrites (both models predicted largest average *d*_*H*_ across the variants for the Ca^2+^ transients 400–600 μm from the soma, these values were 10.41 in the Hay model and 38.8 in the Almog model). In the Almog model, Ca^2+^ dynamics were described also in the basal dendrites, but the Ca^2+^ concentration never reached the smaller threshold in this region (Figure S8A). Unlike in the Hay model, the number of subsequent spikes (*N*_spikes_>1) was highly sensitive to the effects of the variants in the Almog model (Figure S8B), causing a large average *d*_*H*_ (25.62 in the Almog model, 3.46 in the Hay model). Moreover, the correlation between f-I curve integral and predicted neuron output diversity across the variants was slightly larger for small input conductances (0.83) than for large input conductances (0.75) (Table 3C), which was opposite to Hay model predictions. Nevertheless, in a fashion similar to Hay model, the predicted output diversity had an inverted U-shaped curve with respect to the single-synapse conductance (Figure S8C). Except for the *ATP2B2, HCN1*, and *KCNB1* variants, which had a minuscule effect on the output diversity in the Hay model (Figure 6C), the variants had qualitatively similar effects on the output diversity in the two models (Figure 6C and Figure S8C). These results confirm the possibility that the SCZ-associated variants may alter the neural coding capacity in L5PCs in terms of spiking and Ca^2+^-transient output.

**Figure 7:**
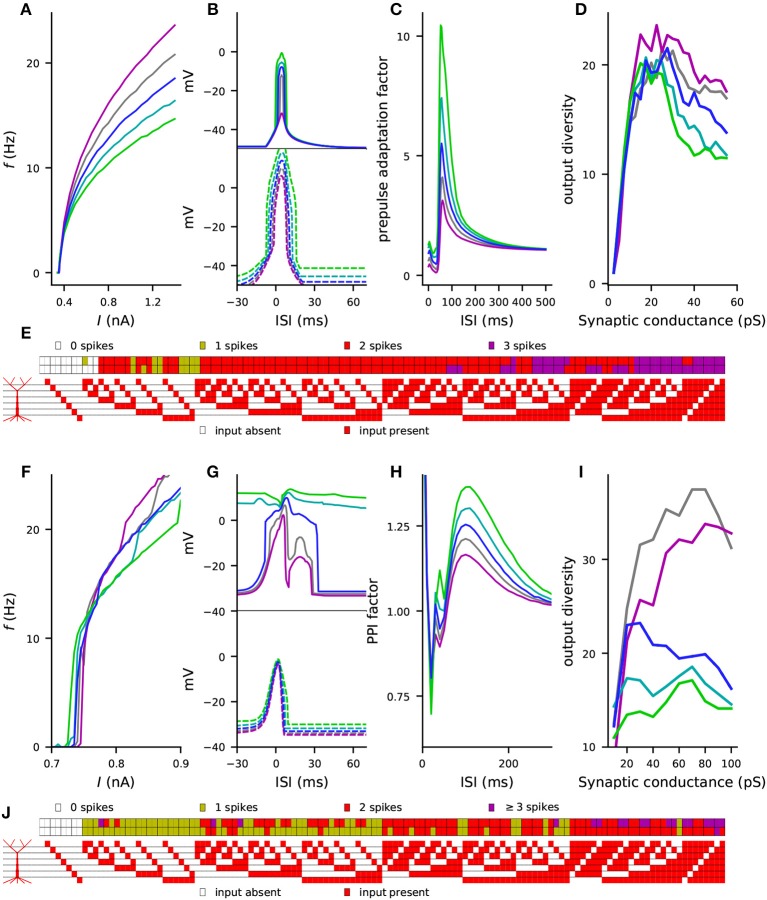
Combinations of variants can cause large effects on L5PC excitability, single-L5PC prepulse adaptation, and coding capacity. **(A–E)** show the Hay model predictions, while **(F–J)** show the Almog model predictions for the combination of variants of genes *CACNA1C, CACNA1D, CACNB2, CACNA1I, ATP2A2, SCN1A*, and *KCNB1*. See Table A2 in Supplementary Material for details on the variants selected for this experiment and Figure S3 for comparison of the steady-state firing behavior of these variants with that of other variants. In panels **(A–D)** and **(F–I)**, the blue curves represent the control neuron behavior, while the other colors represent combinations of differently scaled variants (purple: $\epsilon =\frac{1}{2}$, gray: $\epsilon =\frac{1}{4}$, cyan: $\epsilon =-\frac{1}{4}$, green: $\epsilon =-\frac{1}{2}$). **(A,F)** f-I curves, see Figures 2C,D for single-variant effects. **(B,G)** Windows of sensitivity to paired apical and somatic stimuli, see Figures 3D,E for single-variant effects. The upper panels show the data from the up-state experiment (solid lines), and the lower panels show the data from the down-state experiment (dashed lines). **(C,H)** Prepulse adaptation curves, see Figures 5C,D for single-variant effects. **(D,I)** Output diversity curves, see Figure 6C and Figure S8C for single-variant effects. **(E,J)** Coding by action potentials, see Figure 6A and Figure S8A for single-variant effects. The upper rows in **(E)** and **(J)** represent the control neuron output patterns (number of spikes displayed for each of the 128 input patterns), while the lower rows represent the output patterns of the combination of $\epsilon =\frac{1}{2}$ variants.

Finally, we performed the simulations of the four considered phenotypes (steady-state firing, Figure 2; interaction of the perisomatic region and apical dendrite during up and down states, Figure 3; prepulse-mediated adaptation, Figure 5; and coding capacity, Figure 6) for combinations of variants to illustrate how small genetic effects could produce large effects when combined. From each gene, we chose the $\epsilon =\frac{1}{2}$ variant that gave the maximal increase to the f-I curve integrals in the Hay model, except if all model variants of the underlying gene had negative effects (see Figure S3). Some of these model variants, namely those of *CACNA1C, CACNB2*, and *CACNA1D*, imposed cumulative effects on the same model parameters (see Table A2 in Supplementary Material), but most of them affected different types of transmembrane currents. Figures 7A–D shows the effects of this variant combination on the four phenotypes predicted with the Hay model, and Figures 7F–I shows the corresponding predictions from the Almog model. As expected, the effects of the variant combinations were larger than those of single model variants in Figures 2, 3, 5, 6. The coding by action potentials was also more dramatically affected by the combination of variants (Hamming distance between control and variant output patterns 16 (Hay model, Figure 7E) and 37 (Almog model, Figure 7J) than by the single variants (average Hamming distances 3.46 and 25.62; Figure 6A and Figure S8A). Importantly, the predictions of these phenotypes for the variant combinations followed the trends observed in Table 3: the combination of the variants, scaled as $\epsilon =\frac{1}{2}$ (purple), increased the firing rate of the L5PC (especially for large inputs), suppressed the temporal window of sensitivity to paired apical and somatic stimuli, decreased prepulse-inhibiting ability of the L5PC, and increased its output diversity—da and vice versa for the $\epsilon =-\frac{1}{2}$ scaling (green).

## 4. Discussion

In this work, we built synthetic models of SCZ-associated genetic variants following the framework of Mäki-Marttunen et al. (2016). These models described the effects of common genetic variants on voltage-dependence or kinetics of voltage-gated ion channels or the efficiency of Ca^2+^ transporters, based on functional genomics data from other, rare variants and a previously described downscaling scheme (Mäki-Marttunen et al., 2016). We showed that these model variants affected various properties of L5PC firing and input/output relationships. These properties include steady-state firing (Figure 2), integration of perisomatic and apical dendritic inputs during up and down states (Figure 3), prestimulus-mediated inhibition of a subsequent stimulus (Figure 5), as well as neural coding and output diversity (Figure 6). While effects of single variants were small, combinations of variants showed large effects on these phenotypes (Figure 7). Importantly, our framework enables a correlation analysis between the four phenotypes, i.e., it allows us to predict whether the effects of the variants on a specific phenotype will correlate with those on another phenotype, which can help in discovering their genetic architecture. These predictions are summarized in Table 4. In this work, we showed that the effects of the variants were qualitatively similar in two different neuron models (namely the Hay model Hay et al., 2011 and the Almog model Almog and Korngreen, 2014) and a number of altered models with in-between properties (see Figure 1) of these models (Figures 2, 3, 5). As an additional robustness analysis, we showed that these effects remain qualitatively the same when the L5PC receives noisy, *in vivo*-like inputs (Figure S4 and Figure 4). These results confirm and extend the earlier results of Mäki-Marttunen et al. (2016) that showed Hay-model L5PC sensitivity to variants of SCZ-associated genes: we now have evidence that this sensitivity applies to many types of L5PCs and can alter their coding capacity.

**Table 4:** Summary of the hypothesized effects.

**Phenotype**	**Effect**	**Alternative effect**	**Source**
Steady-state firing	**+**	–	Figure 2
Time window for integration of apical and somatic inputs	**–**	+	Figure 3
Prepulse-mediated adaptation	**–**	+	Figure 5
Neuron output diversity	**+**	–	Figure 6
Delta-band power in L5PC network	**+**	–	Mäki-Marttunen et al., 2018a

*The table shows the effects of SCZ-associated variants (of voltage-gated ion channel and Ca^2+^ transporter-encoding genes) on the four considered single-L5PC phenotypes, i.e., steady-state firing (f-I curve integrals), integration of perisomatic and apical dendritic input (during up state), prestimulus-mediated adaptation, and neuron coding capacity (output diversity). These effects are hypothesized based on the correlation analysis of Table 3. Note that as we do not have data on the effects of the SCZ-associated gene variants but we considered both gain- and loss-of-function variants, the correlation analysis cannot confirm the sign of effect—hence, the two columns. However, based on previous hypotheses on circuit mechanisms as well as behavioral and electrophysiological biomarkers of SCZ (see discussion below), we hypothesized the first column as the more parsimonious alternative for the L5PC-specific effects. The impacts can also be reflected against data from network simulations (Mäki-Marttunen et al., 2018a), where the amplitude of the prepulse adaptation was anticorrelated with both L5PC network gain and the spectral power of the network's response to delta oscillations—hence, the last row of the table*.

L5PCs, likely more than other excitatory neuron types in the cortex (cf. Larkman and Mason, 1990; Markram et al., 2015), express a wide spectrum of subtypes that differ from each other in terms of spiking behavior, morphology, and pattern of projection (Markram et al., 2015). Thick-tufted L5PCs are one of the most thoroughly characterized neocortical cell types, however, there is still a degree of diversity in the properties they are reported to possess. In Le Bé et al. (2006), no rhythmically bursting thick-tufted L5PCs were reported. By contrast, in Chagnac-Amitai et al. (1990), 10% of the thick-tufted L5PCs fired repetitive bursts when stimulated with prolonged depolarizing current. Similar results were obtained in a study of layers V and VI of the prefrontal cortex, but only for pyramidal neurons in layer VI (Yang et al., 1996). It is therefore fitting that of the two models we employed, the Almog model shows rhythmical bursting in response to a prolonged square-pulse current while the Hay model does not. In this work, basing on simulation results, we suggested that the conductance of the voltage-gated Ca^2+^ channels and the magnitude of the SK currents (and possibly the fast Na^+^ channel conductance) are crucial factors in determining this electrical property. Namely, in both Hay and Almog models, decreasing the conductance of the voltage-gated Ca^2+^ channels (which resulted in smaller SK currents) increased the number of spikes within bursts, but this effect was conditional on the conductance of fast Na^+^ channels (Hay model) or SK channels (Almog model) being larger than baseline (Figure 1). Nevertheless, while all the altered Almog models produced typical L5PC-like firing behavior, many of the more radically altered Hay models too easily induced a Ca^2+^-Na^+^ spike when stimulated at the dendrite (see Table S1), although their responses to somatic stimuli were well-constrained (Figure 2C). It is likely that many alternative parameter changes (as well as morphological variations; Hay et al., 2013) could produce similar or more realistic effects on the firing pattern in the Hay model, but a detailed characterization of these relationships is out of the scope of the present work.

Given the variety of models used in this work, it is rather surprising that the model variants have qualitatively similar effects on the firing behavior across the models (e.g., correlation coefficient between the f-I curve integrals of the variants in Hay and Almog models was on average 0.69 ± 0.08; Table S3). This is a reassuring sign of the sensitivity of the L5PC input/output relationship to variants in SCZ-associated ion-channel or Ca^2+^ transporter-encoding genes. Consistency in this regard is important in the light of the morphological and electrophysiological variations in L5PCs within and between species (Beaulieu-Laroche et al., 2018; Fletcher and Williams, 2019). The observation of qualitative similarity between neuron responses to noiseless and noisy inputs (Figure S4 and Figure 4) is less surprising, but nevertheless further confirms the robustness of the predicted effects of SCZ-associated genes on L5PC behavior.

Previously, impairments of apical dendritic functions have been suggested as a cause for mental disorder symptoms. Namely, overexcitability of the apical dendrite was suggested to lead to faulty perceptions whereas its underactivation was suggested to worsen the contextualization of perception (Larkum, 2013; Phillips and Silverstein, 2013). In SCZ, both deficits are expressed on a behavioral level, but it is not known which of the cellular-level deficits, if any, are present in L5PCs. Our results show that variants of SCZ-associated ion-channel and Ca^2+^ transporter-encoding genes can affect the sensitivity of coincidence detection between the soma and the apical dendrite. Moreover, our correlation analyses (Tables 3, 4) lend support for a decreased rather than increased temporal sensitivity of apical-perisomatic integration of inputs, while the overall excitability of the L5PC is increased. This is surprising, as an increased sensitivity to temporally near-coincident inputs would often be considered a sign of increased neuronal excitability. The cause for this mismatch is likely to be the bimodal effects of voltage-gated Ca^2+^ currents in L5PCs due to the SK currents, as analyzed in (Mäki-Marttunen et al., 2017). Let us illustrate this by considering the *CACNA1C* variant of Figures 2, 3, 5 as an example. This variant increased the mid-point voltage of activation and decreased the mid-point voltage of inactivation of the HVA Ca^2+^ channels (Table A2 in Supplementary Material). This should decrease the immediate Ca^2+^ influx in response to activation by depolarizing currents—this is indeed seen in Figure 3E as a decreased membrane potential transient in the dendrite compared to control neuron and thus a narrower temporal window of coincidence detection. However, decreased Ca^2+^ influx during the stimulus means also a decreased activation of SK currents, which would render the neuron more excitable for the subsequent stimuli (Mäki-Marttunen et al., 2017). This is reflected as an increased overall f-I curve (Figures 2C,D) and a decreased threshold for subsequent action potentials (Figures 5E,F) for the particular variant. Therefore, if L5PC activity is affected by SCZ-associated voltage-gated ion-channel and Ca^2+^ transporter-encoding variants, the single-cell excitability is likely to be bimodally affected such that the L5PCs are more excitable when constantly bombarded by excitatory inputs but less excitable when abruptly activated by coincident inputs at the apical dendrite and the perisomatic region. Thus, our results suggest that out of the two hypothesized behavioral phenotypes (Phillips and Silverstein, 2013), an impaired contextualization of perception in SCZ could be due to deficits in integration of sudden, strong inputs to L5PC, while faulty perceptions could be due to increased L5PC firing activity in response to long-lasting barrage of synaptic inputs. However, these speculations assume a common genetic influence of voltage-gated ion-channel and Ca^2+^ transporter-encoding genes on these phenotypes, while in fact many parallel SCZ-associated genetic pathways may interact under various metabolic states (Devor et al., 2017) and contribute to the behavioral phenotypes. Moreover, the SK channels are largely affected by neuromodulation, and thus the contribution of the variants to the abovementioned scenarios may be largely dependent on the neuromodulatory state of the neuron.

Counteracting and compensating genetic and cellular mechanisms that diminish the effects of variants of voltage-gated ion-channel and Ca^2+^ transporter-encoding genes are likely to exist, but they are not considered in this work. Nevertheless, compensatory mechanisms, even if present, would probably not restore all properties of the neurons and neuronal circuits but would rather leave certain other network properties altered in one way or another. For example, synaptic scaling that counteracted the increased L5PC firing did not compensate for a variant-induced increase in delta oscillation power in our previous network modeling study (Mäki-Marttunen et al., 2018a), which is consistent with the qualitative difference between the impact of synaptic scaling on steady-state firing under *in vivo*-like synaptic inputs (Figure S4D) and the effects of our model variants (Figure S4B) reported in this work.

In our discussion of the interaction between apical and somatic zones of integration we have so far concentrated on coincidence detection as its function. However, the particular shape and electrophysiological characteristics of L5PCs allows the apical dendrite to act as a modulator that amplifies or attenuates the cell's responses to its basal inputs (Phillips et al., 2015, 2016). In this mode of operation apical input does not determine whether the L5PC neuron responds or not, but affects how strongly it responds to its basal and perisomatic inputs, i.e., how many action potentials are generated and how large are the induced Ca^2+^ transients. In contrast to the notion of coincidence detection, this perspective on apical function emphasizes the clear asymmetry in the effects of apical and basal input. It is important to see that it may not be the case that apical inputs to L5PCs are dichotomously either driving or amplifying but may be driving or amplifying to different extents. This would then be compatible with computational (Rhodes and Llinás, 2001) and experimental (Zhu, 2000) evidence that inputs to the apical dendrite can also in some cases have a driving effect, either in addition to or instead of having an amplifying effect. There could well be major developmental changes in the balance between driving and amplifying effects of apical depolarization, and this may be a fertile ground for further studies of the genetic bases and pathophysiology of SCZ. For an initial discussion of this matter (see Phillips et al., 2015). We leave a more detailed analysis on how SCZ-associated genes affect the mode of apical function in L5PCs for future work.

The analysis of neural coding in L5PCs (Figure 6 and Figure S8) reveals an additional aspect on how the input/output relationship of an L5PC depends on the SCZ-associated genes. Traditionally, the intracellular Ca^2+^ dynamics have not been considered as a constituent to the output of the neuron in neural coding studies. However, it is known that large Ca^2+^ transients are able to cause long-lasting synaptic changes that are, on a longer time scale, an important contributor to the activity of both the neuron itself and its downstream neurons. We therefore consider both Ca^2+^ transients in the dendrites and the immediate L5PC spiking response (number of action potentials for a given stimulus) as the output of the neuron. According to a widely adopted phenomenological model (reviewed in Evans and Blackwell, 2015), Ca^2+^ transients that exceed a certain (lower) threshold cause long-term depression in the synaptic strength, while Ca^2+^ transients that exceed another (higher) threshold cause potentiation. In this work, we considered these outcomes at each of the 6 (Hay model) or 7 (Almog model) defined dendritic regions separately in addition to the spike count (which was constrained to 3 or less). This means that there were in theory 4 × 3^6^ (Hay) or 4 × 3^7^ (Hay) possible outcomes for a given input. However, Figure 6C and Figure S8C show that only a small fraction (up to 20–40) of these outcomes was obtained for spatially homogeneous inputs of a given amplitude. Interestingly, this fraction, representing the output diversity of the neuron, and its dependence on the strength of the underlying synaptic stimuli were influenced by the SCZ-associated genes.

While a decrease in the output diversity could represent a lowered responsiveness of the neuron, an increased output diversity could by contrast be, when too extreme, a cause for instability in the neuronal network. Limited range of output diversity may also reflect a better metabolic strategy (Barlow, 1961). Table 3D shows that an increased output diversity (for larger synaptic conductances) is strongly associated with a decrease in the predicted adaptation (Figure 5) and an increase in the overall L5PC excitability (Figure 2). These predicted phenotypes can be considered single-cell correlates of a deficit in the PPI of the startle and an excessively efficient integration of sensory and context-dependent inputs, respectively, both of which have been either widely observed in (PPI, Turetsky et al., 2009) or hypothesized to be linked to (excessive sensitivity in hallucinations, Larkum, 2013) SCZ. Supporting evidence for the increased rather than decreased excitability of pyramidal cells in SCZ patients is also obtained from experimental models of SCZ. In Crabtree et al. (2017) and Sun et al. (2018), increased pyramidal cell excitability was observed in medial prefrontal cortical slices of *DISC1*-mutated mice and neuron cultures from 22q11.2-deleted mice, respectively. The increased L5PC excitability could also be an important factor to the increased delta power in SCZ patients (Sponheim et al., 1994; Duan et al., 2015), as suggested by our previous modeling studies (Mäki-Marttunen et al., 2018a,b). This points toward a new hypothesis of SCZ-associated gene variants increasing the neuron output diversity and thus possibly pushing the cortical network to an unstable state. What is meant by this is that the L5PCs of SCZ patients, allowed by the larger neuronal coding capacity, may express more heterogeneity in their modes of spiking and plasticity than those of healthy controls. When this heterogeneity is present in many cells in the local circuits, it might lead to network dynamics that are more sensitive to perturbations. This hypothesis is a cell type-specific (although likely generalizable to other pyramidal neurons), genetically based adaptation of a higher-level hypothesis of an excessive noise in information processing in SCZ (Spitzer and Neumann, 1996; Christensen et al., 2013) (reviewed in Silverstein et al., 2017).

Quantifying the output diversity requires discretization of the continuous-time-continuous-state variables, namely, the membrane potential and intracellular Ca^2+^ concentration. For membrane potential, we only considered the number of action potentials (following the “rate code” paradigm), although their timing might also be important (the “temporal coding” paradigm) (Theunissen and Miller, 1995). For the Ca^2+^ concentration, we only considered the maximal amplitude, although their duration and spatial distribution are also imperative for the plasticity outcome (Evans and Blackwell, 2015). The results obtained in this work are subject to the choice of the thresholds for moderate and high Ca^2+^ amplitudes. These thresholds likely contribute to the exact shape of the output diversity curves as well as other properties of Figure 6 and Figure S8—however, the two models predicted similar results for the effects of the variants, especially in the case of large synaptic drive. Moreover, the stimulus protocol was simple in the sense that all synaptic inputs were activated only once, simultaneously. Future modeling work should address the way different spatio-temporal input patterns affect the output pattern of the neuron. Furthermore, special attention should be paid to the method for quantifying the difference between two output patterns. In this work, we only used the Hamming distance (number of non-identical entries) for quantifying the difference between two output patterns. However, alternative information theoretic measures could be applied, such as normalized compression distance (Li et al., 2004), which was previously used to quantify the differences in several types of dynamical data, including electrocardiogram time courses (Keogh et al., 2004), spiking neuronal network activity (Mäki-Marttunen et al., 2011a), and Boolean network outputs (Mäki-Marttunen et al., 2011b, 2013). In addition, more specialized measures of spike train difference that assess not only the difference between numbers of spikes but also their timing (see, e.g., Shadlen and Newsome, 1998; Jolivet et al., 2008) could be applied. Further information theoretic analysis could be carried out using approaches delineated in Silverstein et al. (2017). These aspects are left for future work.

A significant hindrance in SCZ research is the lack of clearly defined cellular phenotypes that would entail both the genetic background and the symptomatic, functional pathway of the disease as well as symptom manifestations. Two of the most widely suggested SCZ cellular and network-level mechanisms, namely, the NMDA receptor hypofunction hypothesis (Olney et al., 1999) and the hypothesis on GABAergic interneuron dysfunctions (Nakazawa et al., 2012), come close to such a concept, although these phenotypes capture only a fraction of the genetic pathways implicated in SCZ (Devor et al., 2017). Here, we compared the effects of our model variants on L5PC firing to the modeled effects NMDA and GABA hypofunctions. In our single-neuron experiments, where the L5PC was bombarded by spontaneous glutamatergic and GABAergic inputs in addition to a perisomatic depolarizing current, we showed that changes in NMDA- and GABA-receptor conductance led to altered firing behavior across the perisomatic stimulus intensities; see Figure S4D). Thus, these alterations contribute to changes in L5PC excitability, but not to altered L5PC gain as many of the model variants of especially Ca^2+^ channel and transporter-encoding genes (see Figure 2). Nevertheless, both previously applied synaptic impairments and our altered voltage-gated ion-channel functions have been shown to lead to SCZ-like abnormalities in neural oscillations and spectral responses in network simulations (Carlen et al., 2012; Metzner et al., 2016; Mäki-Marttunen et al., 2018a). Moreover, increased intrinsic pyramidal cell excitability (caused by variants of ion-channel-encoding genes as studied here) could lead to increased E/I ratio and glutamate spillover in a fashion similar to the hypothesized mechanism of NMDA receptor hypofunction in GABAergic neurons (Nakazawa et al., 2017) and could have symptomatic consequences as well (Phillips et al., 2016). Thus, common variants of voltage-gated ion-channel-encoding genes could be considered another plausible pathway integrating new aspects of SCZ genomics with hypothesized cellular and network-level disease mechanisms of SCZ. The effects of these variants on SCZ pathophysiology may depend on the variants more tightly connected to the NMDA receptor hypofunctions, but the existence and nature of these interactions remains speculative at the moment.

Our downscaling scheme is a theoretical approach for exploring the effects of common variants of SCZ-associated genes based on the electrophysiological properties of other variants of the same genes (Mäki-Marttunen et al., 2016). This approach was used as there are currently no data on electrophysiological effects of SCZ-associated genetic variants. It should, however, be noted that the effects of SCZ-associated genetic variants may also be mediated by altered gene-regulation mechanisms, and not only by differences in the functions of wild-type and variant proteins. Moreover, it is possible that the SCZ-associated genetic variants have non-linear, systemic effects more complex than the ones predicted in Figure 7. Nevertheless, our results provide an analysis for effects of many kinds of genetic alterations in the associated genes. The changes in voltage-dependence or kinetics of the modeled transmembrane currents, as listed in Table A1 (Supplementary Material), could be caused by altered balance of channel-subunit expression (cf. Isom et al., 1994; Brackenbury and Isom, 2011) as well as directly by variants in the protein-coding domains. Experimental studies should be carried out to confirm which of the two modes of alterations, if either, are caused by the SCZ-associated genetic variants. Future modeling work could pinpoint specific types of input patterns for which the L5PC responses are altered by the SCZ-associated variants and thus help make hypotheses on the altered single-cell and circuit behavior in the mental disease. A challenge for future computational and experimental studies is to find out how our hypothesis of altered voltage-gate ion-channel and Ca^2+^-transporter functions in SCZ compares to or complements the previously proposed hypotheses on SCZ, such as the NMDA receptor hypofunction hypothesis (Olney et al., 1999) and the hypothesis on GABAergic interneuron dysfunctions (Nakazawa et al., 2012).

## Data Availability Statement

All datasets generated for this study are included in the manuscript, Supplementary Files, and/or ModelDB entry 249463.

## Author Contributions

TM-M, AD, AMD, OA, and GE designed the study. WP provided data or analytical support. TM-M performed the analysis and wrote the manuscript. TM-M, AD, WP, AMD, OA, and GE interpreted the results.

### Conflict of Interest

The authors declare that the research was conducted in the absence of any commercial or financial relationships that could be construed as a potential conflict of interest.

## Footnotes

^1^The molecular basis of LVA Ca^2+^ currents in L5PCs is disputed. Experimental data shows that T-type Ca^2+^ currents are not present in the soma of an L5PC nor its neighborhood (Almog and Korngreen, 2009). Therefore, the role of low or medium-voltage activated Ca^2+^ channels was assigned to R-type channels (α pore encoded by *CACNA1E* gene) in the Almog model (Almog and Korngreen, 2014). In the Hay model, however, the LVA channel kinetics were based on measurements carried out on T-type Ca^2+^ channels. In this work, we consider the T-type Ca^2+^ channel gene *CACNA1I* to affect both the Almog-model *I*_*CaMVA*_ current and the Hay-model *I*_*CaLVA*_.
