# Supplementary material for: Computational Modeling of Genetic Contributions to Excitability and Neural Coding in Layer V Pyramidal Cells: Applications to Schizophrenia Pathology
Source: Front Comput Neurosci. 2019 Sep 26;13:66. doi: 10.3389/fncom.2019.00066 (PMC6775251; doi:10.3389/fncom.2019.00066)
Supplement: Supplementary file 1 [file Data_Sheet_1.pdf]

Computational modeling of genetic contributions to excitability and neural coding in layer V pyramidal cells: applications to schizophrenia pathology. Appendix: Tables A1 and A2.

Table A1: **Table of the genetic variants used in this study.** The first column of the table shows the gene that the variant studied in the named reference affected. The second and third columns show the current species and the model parameters that are affected by the variant, "offm" and "offh" meaning the mid-points of activation and inactivation, respectively, "slom" and "sloh" their slopes, and "taum" and "tauh" their time constants. These parameter names may refer to multiple model parameters: For example, for HVA Ca^{2+} currents, a change in "offm" means a concurrent change in parameters V_{offma} and V_{offmb}, while "offh" refers to parameters V_{offha} and V_{offhb}, "slom" to V_{sloma} and V_{slomb}, "sloh" to V_{sloha} and V_{slohb}, "taum" to τ_{ma} and τ_{mb}, and "tauh" to τ_{ha} and τ_{hb}. See model equations for details. The fourth column shows the direction and magnitude of the effect, ±x mV referring to a shift of the middle-point of the (in)activation curve by an absolute number of millivolts, and ±x% referring to a percentage change in the underlying quantity. In some cases, several variants were considered in a single study. Here, the variants are categorized by their type when necessary (e.g., in [1] several variants of four loci, of which three were in pore-lining IS6 segment and two in bundle-crossing region of segment IIS6, were considered, and the variants are here categorized according to the segment they acted on). The fifth column names the type of variant used, while the sixth and seventh columns show the cell type in which the effects are measured and the animal species used in the study. The final column gives relevant extra information. The variants are listed in the same order as in Table A1. The table is an extended version of the corresponding table in Mäki-Marttunen et al. (2016).

Gene	Current	Parameter	Effect	Type of variant	Cell type	Animal	Notes
CACNA1C [1]	I_{CaHVA}	offm<br>offh	-25.9..-1.4mV<br>-27.0..-3.8mV	L429T, L434T, S435T, S435A, S435P	TSA201	human	These represent variant of the IS6 segment
CACNA1C [1]	I_{CaHVA}	offm<br>offh	-37.3..-9.7mV<br>-30.0..-11.8mV	L779T, I781T, I781P	TSA201	human	These are variants of IIS6 segment. Double (IIS6+IS6) mutant effects seem additive
CACNA1C [2]	I_{CaHVA}	offm<br>slom<br>offh<br>sloh	-31.4..+7.0mV<br>-15..+45%<br>-28.5..+16.3mV<br>-28%..+38%	G432X, A780X, G1193X, A1503X	TSA201	human / rabbit	Double mutant G432S/S435G not considered
CACNA1C [3]	I_{CaHVA}	offm<br>slom	-38.5..+12.9mV<br>-54..+56%	I781X, C769P, G770P, N771P, I773P, F778P, L779P, A780P, A782P, V783P	TSA201	human	Data for co-expression with both b1a and b2a. Here, the maximal deviation from the corresponding wild-type value taken
CACNA1C [4]	I_{CaHVA}	offm<br>slom<br>offh	-27.8..+8.7mV<br>-11%..+14%<br>-19.1..+4.7mV	I781T, N785A, N785G, N785L	TSA201	human	Double mutants not included (effects do not seem additive)
CACNA1C [5]	I_{CaHVA}	offm<br>offh<br>sloh	-11.2..+1.0mV<br>-3.1..-0.3mV<br>+3%..+24%	Splice variants a1C77-A, -B, -C and -D	TSA201	human	Slopes for activation curves visibly different, but fits not carried out (do not fit well to Boltzmann curve)
CACNA1D [6] [7]	I_{CaHVA}	offm<br>slom<br>offh<br>sloh<br>tauh	-10.9..-8.5mV<br>-27..-13%<br>-3.0..+3.5mV<br>-12..-19%<br>+25%	Splice variant 42A	TSA201 / HEK293	human / rat / mouse	The splice variant is expressed in rat and human brain, thus both signs of effects could be possible. Here, the long form was considered the "control" and the short form (42A) the variant
CACNA1D [6] [7]	I_{CaHVA}	offm<br>slom<br>offh<br>sloh<br>tauh	-10.6..+3.4mV<br>-20..+12%<br>-5.3..+1.2mV<br>-34..-8%<br>-28%	Splice variant 43S	TSA201 / HEK293	human / rat / mouse	The splice variant is expressed in rat and human brain, thus both signs of effects could be possible. Here, the long form was considered the "control" and the short form (43S) the variant
CACNA1D [8] [9]	I_{CaHVA}	offm<br>slom<br>tauh	+3.5..+6.6mV<br>-25..+19%<br>-50..+12%	Homozygous knock-out	AV node / chromaffin cells	mouse	Some time constants compared between single tau fits (as double tau fits not always well fitted)
CACNA1D [10]	I_{CaHVA}	offm<br>slom<br>offh<br>sloh	-9.8mV<br>-20%<br>-15.4mV<br>+5%	A749G	TSA201	human	Also G407R and co-transfection with WT studied, but in these cases the inactivation was too deficient to be included here
CACNA1D [11]	I_{CaHVA}	offm<br>slom<br>offh<br>sloh<br>tauh	-24.2..+6.1mV<br>-30..+24%<br>-14.5..-3.6mV<br>-28%..+28%<br>+43%..+252%	V259D, I750M, P1336R	TSA201	human	
CACNA1D [12]	I_{CaHVA}	offm<br>slom<br>tauh	-17.8..-13.1mV<br>-19..-0%<br>-23%..+31%	rCav1.3scg variant and related mutants 7M2K, S244G, V1104A, and A2075V	TSA201	human / rat	Double/triple mutants studied as well but they did not produce any stronger effects. Effects measured w.r.t. rCav1.3L
CACNB2 [13]	I_{CaHVA}	offh<br>sloh	-5.2mV<br>-31%	T11I	TSA201	human	Small effects on offm ignored
CACNB2 [14]	I_{CaHVA}	taum	+70%	A1B2 vs A1 alone	HEK293	human / mouse	
CACNB2 [15]	I_{CaHVA}	offm<br>offh<br>taum<br>tauh	-4.9..+4.9mV<br>-5.1..+5.1mV<br>-40%..+68%<br>-40%..+66%	Splice variants N1, N3, N4, and N5	HEK293	human / mouse	
CACNB2 [16]	I_{CaHVA}	tauh	+26%	D601E	TSA201	human	
CACNA1I [17]	I_{CaLVA}	offm<br>offh<br>taum<br>tauh	-0.2..+1.3mV<br>-0.5..+1.6mV<br>-13..+45%<br>-20..+8%	Alternative splicing of exons 9 and 33	HEK293	human	Maximum of effects at -40 or 0 mV on kinetics considered. Changes in slopes were minuscule and thus ignored
CACNA1I [18]	I_{CaLVA}	offm<br>slom<br>offh<br>sloh<br>taum<br>tauh	-4.3..-1.2mV<br>+5..+14%<br>-4.4..-1.9mV<br>-11%..+4%<br>-47%..-15%<br>-54%..+1%	Truncated cDNAs L4, L6 and L9	HEK293	human / rat	Changes in slopes and decays nonsignificant. Slopes included but time constants not (diverse asymptotes make comparison difficult)
ATP2A2 [19]	(none)	γ	-30..-40%	Heterozygous null mutation	myocytes	mouse	
ATP2B2 [20]	(none)	τ_{decay}	+15..+113%	Heterozygous knockout	Purkinje cells	mouse	Compensatory mechanisms may prevent larger effects
ATP2B2 [21]	(none)	τ_{decay}<br>c_{min}	+32..+50%<br>+40%	Homozygous knockout	Purkinje cells	mouse	
ATP2B2 [22]	(none)	τ_{decay}	+53..+345%	G283S-, G293S-mutant	CHO	human / mouse	+53% for G293S, +345% for G283S
ATP2B2 [23]	(none)	c_{min}	+10%	E584K-, T692K-mutant	Hair cells	human / mouse	Other smaller-effect variants studied too
SCN1A [24]	I_{Nat}	offm<br>offh<br>slom<br>sloh	-0.3mV<br>+5.0mV<br>+15%<br>+23%	Q1489K	Cultured neocortical cells	human / rat	Slow inactivation could not be studied in detail in neurons
SCN1A [25]	I_{Nat}	offm<br>offh<br>slom<br>sloh	+2.8mV<br>+6.3..+9.6mV<br>-1.6%<br>+4.2%	L1649Q	TSA201	human	Electrophysiology done with the corresponding mutation L1636Q in the homologous SCN5A gene due to instabilities in recombinant bacteria
SCN1A [26]	I_{Nat}	offm<br>offh<br>slom<br>sloh<br>tauh	-4.0mV<br>-5.8mV<br>-8%<br>+13%<br>+43..+47%	R859H	TSA201	human	Persistent current also modified in mutant channels, but this is not taken into account here
SCN1A [26]	I_{Nat}	offm<br>offh<br>slom<br>sloh<br>tauh	-8.1mV<br>+2.2mV<br>-3%<br>-3%<br>+26..+59%	R865G	TSA201	human	Persistent current also modified in mutant channels, but this is not taken into account here
SCN1A [27]	I_{Nat}	offm<br>slom<br>tauh	+6.0mV<br>+16%<br>+29%	T1174S	TSA201	human	Data pooled with hbeta1 and hbeta2 subunit coexpression. Difference in inactivation nonsignificant
SCN1A [28]	I_{Nat}	offm<br>offh<br>slom<br>sloh	+10.0mV<br>-0.6mV<br>+15%<br>+14%	M145T	TSA201	human	

Table A1 continued.

Gene		Current	Parameter	Effect	Type of variant	Cell type	Animal	Notes
HCN1	[29]	I_h	offh<br>sloh	-2.1..-26.5mV<br>-12..-36%	D135W, D135H, D135N	HEK293	mouse	Other mutations studied as well, but they changed the current too radically
HCN1	[30]	I_h	offh<br>sloh	-25.9..+17.7mV<br>-40..+3%	E229A, K230A, G231A, M232A, D233A, S234A, E235G, V236A, Y237A, EVY235-237DDD	Oocytes	mouse / xenopus	
KCNB1	[31]	I_{Kp}	offm<br>offh<br>slom<br>sloh<br>taum<br>tauh	+5mV<br>+3mV<br>+11%<br>-14%<br>-50%<br>-47%	T203K mutant	HEK293	human / mouse	Double mutations studied as well, but they are not included here
KCNB1	[31]	I_{Kp}	offm<br>offh<br>slom<br>sloh<br>taum<br>tauh	+1mV<br>-6mV<br>+22%<br>+0%<br>-11%<br>-13%	T203D mutant	HEK293	human / mouse	
KCNB1	[31]	I_{Kp}	offm<br>offh<br>slom<br>sloh<br>taum<br>tauh	+6mV<br>-8mV<br>+33%<br>+0%<br>-50%<br>-13%	S347K mutant	HEK293	human / mouse	
KCNB1	[31]	I_{Kp}	offm<br>offh<br>slom<br>sloh<br>taum<br>tauh	-28mV<br>-27mV<br>+11%<br>-29%<br>+13%<br>+127%	S347D mutant	HEK293	human / mouse	
KCNB1	[31]	I_{Kp}	offm<br>offh<br>slom<br>sloh<br>taum<br>tauh	+14mV<br>-21mV<br>+100%<br>+0%<br>-61%<br>+20%	T203W mutant	HEK293	human / mouse	
KCNB1	[31]	I_{Kp}	offm<br>offh<br>slom<br>sloh<br>taum<br>tauh	-13mV<br>-13mV<br>+33%<br>-29%<br>-5%<br>+413%	S347W mutant	HEK293	human / mouse	
KCNMA1	[32]	I_{BK}	offm<br>taum	-13.8..+57.0mV<br>-49..+175%	E912A, D916A, N918A Q920A, D923A mutants	HEK293	mouse / human	Double mutant not included here
KCNMA1	[33]	I_{BK}	offm	+27.8..+49.2mV	Slo1C-KvT, Slo1C-Kv-minT	Oocytes	mouse / xenopus	
KCNMA1	[34]	I_{BK}	offm<br>taum	0..+16mV<br>+491%	hbr5	Oocytes	human / xenopus	Effects estimated from figure
KCNMA1	[35]	I_{BK}	offm<br>taum	-5..+15mV<br>-87..0%	e9alt, e9+e9alt	HEK293	human / mouse	Effects estimated from figure
KCNMA1	[36]	I_{BK}	offm	-58..+3mV	e20, e21(STREX), e22	HEK293	mouse / human	

Table A2: **Table for the effects of the genetic variants on model parameters.** The first column names the gene and the study in which the gene variant was analyzed. The second column shows the effect of the variant on the model parameters, “offm” and “offh” meaning the mid-points of activation and inactivation, respectively, “slom” and “sloh” their slopes, and “taum” and “tauh” their time constants. The third column shows the threshold scaling parameter of the variant. all models. The rows separated by horizontal lines correspond to different entries of Table A1: If the corresponding study showed a large range of effects on single model parameters, the endpoints of such ranges are here treated as different variants that are downscaled independently of each other. Stars (⋆) mark the variants used in Figure **??**, and the crosses (×) mark the variants included in the combination of variants in Figure **??**. The final two columns show the scaling coefficients in the unaltered Hay and Almog models — see Table S2 for the data with the altered models.

Gene	Parameter changes	c_{Hay}	c_{Almog}	
CACNA1C [1]	$V_{offm*,CaHVA}$: -25.9 mV; $V_{offh*,CaHVA}$: -27.0 mV	c = 0.066	c = 0.095	
CACNA1C [1]	$V_{offm*,CaHVA}$: -37.3 mV; $V_{offh*,CaHVA}$: -30.0 mV	c = 0.042	c = 0.059	
CACNA1C [2]	$V_{offm*,CaHVA}$: -31.4 mV; $V_{slom*,CaHVA}$: *0.85; $V_{offh*,CaHVA}$: -28.5 mV; $V_{sloh*,CaHVA}$: *0.72	c = 0.043	c = 0.070	
	$V_{offm*,CaHVA}$: +7.0 mV; $V_{slom*,CaHVA}$: *0.85; $V_{offh*,CaHVA}$: -28.5 mV; $V_{sloh*,CaHVA}$: *0.72	c = 0.101	c = 0.222	
	$V_{offm*,CaHVA}$: -31.4 mV; $V_{slom*,CaHVA}$: *1.45; $V_{offh*,CaHVA}$: -28.5 mV; $V_{sloh*,CaHVA}$: *0.72	c = 0.049	c = 0.074	
	$V_{offm*,CaHVA}$: +7.0 mV; $V_{slom*,CaHVA}$: *1.45; $V_{offh*,CaHVA}$: -28.5 mV; $V_{sloh*,CaHVA}$: *0.72	c = 0.076	c = 0.268	
	$V_{offm*,CaHVA}$: -31.4 mV; $V_{slom*,CaHVA}$: *0.85; $V_{offh*,CaHVA}$: +16.3 mV; $V_{sloh*,CaHVA}$: *0.72	c = 0.031	c = 0.065	
	$V_{offm*,CaHVA}$: +7.0 mV; $V_{slom*,CaHVA}$: *0.85; $V_{offh*,CaHVA}$: +16.3 mV; $V_{sloh*,CaHVA}$: *0.72	c = 0.208	c = 0.263	
	$V_{offm*,CaHVA}$: -31.4 mV; $V_{slom*,CaHVA}$: *1.45; $V_{offh*,CaHVA}$: +16.3 mV; $V_{sloh*,CaHVA}$: *0.72	c = 0.034	c = 0.067	
	$V_{offm*,CaHVA}$: +7.0 mV; $V_{slom*,CaHVA}$: *1.45; $V_{offh*,CaHVA}$: +16.3 mV; $V_{sloh*,CaHVA}$: *0.72	c = 0.290	c = 0.269	
	$V_{offm*,CaHVA}$: -31.4 mV; $V_{slom*,CaHVA}$: *0.85; $V_{offh*,CaHVA}$: -28.5 mV; $V_{sloh*,CaHVA}$: *1.38	c = 0.058	c = 0.070	
	$V_{offm*,CaHVA}$: +7.0 mV; $V_{slom*,CaHVA}$: *0.85; $V_{offh*,CaHVA}$: -28.5 mV; $V_{sloh*,CaHVA}$: *1.38	c = 0.059	c = 0.262	⋆ ×
	$V_{offm*,CaHVA}$: -31.4 mV; $V_{slom*,CaHVA}$: *1.45; $V_{offh*,CaHVA}$: -28.5 mV; $V_{sloh*,CaHVA}$: *1.38	c = 0.071	c = 0.078	
	$V_{offm*,CaHVA}$: +7.0 mV; $V_{slom*,CaHVA}$: *1.45; $V_{offh*,CaHVA}$: -28.5 mV; $V_{sloh*,CaHVA}$: *1.38	c = 0.049	c = 0.258	
	$V_{offm*,CaHVA}$: -31.4 mV; $V_{slom*,CaHVA}$: *0.85; $V_{offh*,CaHVA}$: +16.3 mV; $V_{sloh*,CaHVA}$: *1.38	c = 0.038	c = 0.077	
	$V_{offm*,CaHVA}$: +7.0 mV; $V_{slom*,CaHVA}$: *0.85; $V_{offh*,CaHVA}$: +16.3 mV; $V_{sloh*,CaHVA}$: *1.38	c = 0.176	c = 0.259	
	$V_{offm*,CaHVA}$: -31.4 mV; $V_{slom*,CaHVA}$: *1.45; $V_{offh*,CaHVA}$: +16.3 mV; $V_{sloh*,CaHVA}$: *1.38	c = 0.038	c = 0.067	
	$V_{offm*,CaHVA}$: +7.0 mV; $V_{slom*,CaHVA}$: *1.45; $V_{offh*,CaHVA}$: +16.3 mV; $V_{sloh*,CaHVA}$: *1.38	c = 0.113	c = 0.304	
CACNA1C [3]	$V_{offm*,CaHVA}$: -38.5 mV; $V_{slom*,CaHVA}$: *0.46	c = 0.028	c = 0.050	
	$V_{offm*,CaHVA}$: +12.9 mV; $V_{slom*,CaHVA}$: *0.46	c = 0.123	c = 0.143	
	$V_{offm*,CaHVA}$: -38.5 mV; $V_{slom*,CaHVA}$: *1.56	c = 0.035	c = 0.064	
	$V_{offm*,CaHVA}$: +12.9 mV; $V_{slom*,CaHVA}$: *1.56	c = 0.063	c = 0.165	
CACNA1C [4]	$V_{offm*,CaHVA}$: -27.8 mV; $V_{slom*,CaHVA}$: *0.89; $V_{offh*,CaHVA}$: -19.1 mV	c = 0.052	c = 0.054	
	$V_{offm*,CaHVA}$: +8.7 mV; $V_{slom*,CaHVA}$: *0.89; $V_{offh*,CaHVA}$: -19.1 mV	c = 0.077	c = 0.220	
	$V_{offm*,CaHVA}$: -27.8 mV; $V_{slom*,CaHVA}$: *1.14; $V_{offh*,CaHVA}$: -19.1 mV	c = 0.057	c = 0.078	
	$V_{offm*,CaHVA}$: +8.7 mV; $V_{slom*,CaHVA}$: *1.14; $V_{offh*,CaHVA}$: -19.1 mV	c = 0.069	c = 0.251	
	$V_{offm*,CaHVA}$: -27.8 mV; $V_{slom*,CaHVA}$: *0.89; $V_{offh*,CaHVA}$: +4.7 mV	c = 0.042	c = 0.079	
	$V_{offm*,CaHVA}$: +8.7 mV; $V_{slom*,CaHVA}$: *0.89; $V_{offh*,CaHVA}$: +4.7 mV	c = 0.145	c = 0.252	
	$V_{offm*,CaHVA}$: -27.8 mV; $V_{slom*,CaHVA}$: *1.14; $V_{offh*,CaHVA}$: +4.7 mV	c = 0.044	c = 0.062	
	$V_{offm*,CaHVA}$: +8.7 mV; $V_{slom*,CaHVA}$: *1.14; $V_{offh*,CaHVA}$: +4.7 mV	c = 0.119	c = 0.183	
CACNA1C [5]	$V_{offm*,CaHVA}$: -11.2 mV; $V_{offh*,CaHVA}$: -3.1 mV; $V_{sloh*,CaHVA}$: *1.24	c = 0.157	c = 0.188	
	$V_{offm*,CaHVA}$: +1.0 mV; $V_{offh*,CaHVA}$: -3.1 mV; $V_{sloh*,CaHVA}$: *1.24	c = 0.236	c = 1.625	
CACNA1D [6], [7]	$V_{offm*,CaHVA}$: -10.9 mV; $V_{slom*,CaHVA}$: *0.73; $V_{offh*,CaHVA}$: -3.0 mV; $V_{sloh*,CaHVA}$: *0.81; $\tau_{h*,CaHVA}$: *1.25	c = 0.083	c = 0.183	
	$V_{offm*,CaHVA}$: -10.9 mV; $V_{slom*,CaHVA}$: *0.73; $V_{offh*,CaHVA}$: +3.5 mV; $V_{sloh*,CaHVA}$: *0.81; $\tau_{h*,CaHVA}$: *1.25	c = 0.075	c = 0.187	
CACNA1D [6], [7]	$V_{offm*,CaHVA}$: -10.6 mV; $V_{slom*,CaHVA}$: *0.8; $V_{offh*,CaHVA}$: -5.3 mV; $V_{sloh*,CaHVA}$: *0.66; $\tau_{h*,CaHVA}$: *0.72	c = 0.080	c = 0.221	
	$V_{offm*,CaHVA}$: +3.4 mV; $V_{slom*,CaHVA}$: *0.8; $V_{offh*,CaHVA}$: -5.3 mV; $V_{sloh*,CaHVA}$: *0.66; $\tau_{h*,CaHVA}$: *0.72	c = 0.424	c = 0.502	
	$V_{offm*,CaHVA}$: -10.6 mV; $V_{slom*,CaHVA}$: *1.12; $V_{offh*,CaHVA}$: -5.3 mV; $V_{sloh*,CaHVA}$: *0.66; $\tau_{h*,CaHVA}$: *0.72	c = 0.094	c = 0.215	
	$V_{offm*,CaHVA}$: +3.4 mV; $V_{slom*,CaHVA}$: *1.12; $V_{offh*,CaHVA}$: -5.3 mV; $V_{sloh*,CaHVA}$: *0.66; $\tau_{h*,CaHVA}$: *0.72	c = 0.663	c = 0.506	
	$V_{offm*,CaHVA}$: -10.6 mV; $V_{slom*,CaHVA}$: *0.8; $V_{offh*,CaHVA}$: +1.2 mV; $V_{sloh*,CaHVA}$: *0.66; $\tau_{h*,CaHVA}$: *0.72	c = 0.072	c = 0.213	
	$V_{offm*,CaHVA}$: +3.4 mV; $V_{slom*,CaHVA}$: *0.8; $V_{offh*,CaHVA}$: +1.2 mV; $V_{sloh*,CaHVA}$: *0.66; $\tau_{h*,CaHVA}$: *0.72	c = 0.274	c = 0.500	
	$V_{offm*,CaHVA}$: -10.6 mV; $V_{slom*,CaHVA}$: *1.12; $V_{offh*,CaHVA}$: +1.2 mV; $V_{sloh*,CaHVA}$: *0.66; $\tau_{h*,CaHVA}$: *0.72	c = 0.083	c = 0.212	
	$V_{offm*,CaHVA}$: +3.4 mV; $V_{slom*,CaHVA}$: *1.12; $V_{offh*,CaHVA}$: +1.2 mV; $V_{sloh*,CaHVA}$: *0.66; $\tau_{h*,CaHVA}$: *0.72	c = 0.341	c = 0.625	
CACNA1D [8], [9]	$V_{offm*,CaHVA}$: +6.6 mV; $V_{slom*,CaHVA}$: *0.75; $\tau_{h*,CaHVA}$: *0.5	c = 0.190	c = 0.283	
	$V_{offm*,CaHVA}$: +6.6 mV; $V_{slom*,CaHVA}$: *1.19; $\tau_{h*,CaHVA}$: *0.5	c = 0.123	c = 0.242	
	$V_{offm*,CaHVA}$: +6.6 mV; $V_{slom*,CaHVA}$: *0.75; $\tau_{h*,CaHVA}$: *1.12	c = 0.209	c = 0.244	
	$V_{offm*,CaHVA}$: +6.6 mV; $V_{slom*,CaHVA}$: *1.19; $\tau_{h*,CaHVA}$: *1.12	c = 0.130	c = 0.246	
CACNA1D [10]	$V_{offm*,CaHVA}$: -9.8 mV; $V_{slom*,CaHVA}$: *0.8; $V_{offh*,CaHVA}$: -15.4 mV; $V_{sloh*,CaHVA}$: *1.05	c = 0.181	c = 0.225	
CACNA1D [11]	$V_{offm*,CaHVA}$: -24.2 mV; $V_{slom*,CaHVA}$: *0.7; $V_{offh*,CaHVA}$: -14.5 mV; $V_{sloh*,CaHVA}$: *0.72; $\tau_{h*,CaHVA}$: *3.52	c = 0.045	c = 0.083	
	$V_{offm*,CaHVA}$: +6.1 mV; $V_{slom*,CaHVA}$: *0.7; $V_{offh*,CaHVA}$: -14.5 mV; $V_{sloh*,CaHVA}$: *0.72; $\tau_{h*,CaHVA}$: *3.52	c = 0.318	c = 0.259	×
	$V_{offm*,CaHVA}$: -24.2 mV; $V_{slom*,CaHVA}$: *1.24; $V_{offh*,CaHVA}$: -14.5 mV; $V_{sloh*,CaHVA}$: *0.72; $\tau_{h*,CaHVA}$: *3.52	c = 0.053	c = 0.088	
	$V_{offm*,CaHVA}$: +6.1 mV; $V_{slom*,CaHVA}$: *1.24; $V_{offh*,CaHVA}$: -14.5 mV; $V_{sloh*,CaHVA}$: *0.72; $\tau_{h*,CaHVA}$: *3.52	c = 0.152	c = 0.263	
	$V_{offm*,CaHVA}$: -24.2 mV; $V_{slom*,CaHVA}$: *0.7; $V_{offh*,CaHVA}$: -14.5 mV; $V_{sloh*,CaHVA}$: *1.28; $\tau_{h*,CaHVA}$: *3.52	c = 0.059	c = 0.095	
	$V_{offm*,CaHVA}$: +6.1 mV; $V_{slom*,CaHVA}$: *0.7; $V_{offh*,CaHVA}$: -14.5 mV; $V_{sloh*,CaHVA}$: *1.28; $\tau_{h*,CaHVA}$: *3.52	c = 0.105	c = 0.282	
	$V_{offm*,CaHVA}$: -24.2 mV; $V_{slom*,CaHVA}$: *1.24; $V_{offh*,CaHVA}$: -14.5 mV; $V_{sloh*,CaHVA}$: *1.28; $\tau_{h*,CaHVA}$: *3.52	c = 0.074	c = 0.096	
	$V_{offm*,CaHVA}$: +6.1 mV; $V_{slom*,CaHVA}$: *1.24; $V_{offh*,CaHVA}$: -14.5 mV; $V_{sloh*,CaHVA}$: *1.28; $\tau_{h*,CaHVA}$: *3.52	c = 0.076	c = 0.313	
CACNA1D [12]	$V_{offm*,CaHVA}$: -17.8 mV; $V_{slom*,CaHVA}$: *0.81; $\tau_{h*,CaHVA}$: *0.77	c = 0.065	c = 0.126	
	$V_{offm*,CaHVA}$: -17.8 mV; $V_{slom*,CaHVA}$: *0.81; $\tau_{h*,CaHVA}$: *1.31	c = 0.063	c = 0.116	

Table A2 continued.

Gene	Parameter changes	c_{Hay}	c_{Almog}
CACNB2 [13]	$V_{offh*,CaHVA}$: -5.2 mV; $V_{sloh*,CaHVA}$: *0.69	c = 0.371	c > 2.000
CACNB2 [14]	$\tau_{h*,CaHVA}$: *1.7	c > 2.000	c > 2.000
CACNB2 [15]	$V_{offm*,CaHVA}$: -4.9 mV; $V_{offh*,CaHVA}$: -5.1 mV; $\tau_{m*,CaHVA}$: *0.6; $\tau_{h*,CaHVA}$: *0.6	c = 0.194	c = 0.537
	$V_{offm*,CaHVA}$: +4.9 mV; $V_{offh*,CaHVA}$: -5.1 mV; $\tau_{m*,CaHVA}$: *0.6; $\tau_{h*,CaHVA}$: *0.6	c = 0.278	c = 0.364
	$V_{offm*,CaHVA}$: -4.9 mV; $V_{offh*,CaHVA}$: +5.1 mV; $\tau_{m*,CaHVA}$: *0.6; $\tau_{h*,CaHVA}$: *0.6	c = 0.131	c = 0.569
	$V_{offm*,CaHVA}$: +4.9 mV; $V_{offh*,CaHVA}$: +5.1 mV; $\tau_{m*,CaHVA}$: *0.6; $\tau_{h*,CaHVA}$: *0.6	c = 1.153	c = 0.409
	$V_{offm*,CaHVA}$: -4.9 mV; $V_{offh*,CaHVA}$: -5.1 mV; $\tau_{m*,CaHVA}$: *1.68; $\tau_{h*,CaHVA}$: *0.6	c = 1.201	c = 0.449
	$V_{offm*,CaHVA}$: +4.9 mV; $V_{offh*,CaHVA}$: -5.1 mV; $\tau_{m*,CaHVA}$: *1.68; $\tau_{h*,CaHVA}$: *0.6	c = 0.105	c = 0.384
	$V_{offm*,CaHVA}$: -4.9 mV; $V_{offh*,CaHVA}$: +5.1 mV; $\tau_{m*,CaHVA}$: *1.68; $\tau_{h*,CaHVA}$: *0.6	c = 0.373	c = 0.453
	$V_{offm*,CaHVA}$: +4.9 mV; $V_{offh*,CaHVA}$: +5.1 mV; $\tau_{m*,CaHVA}$: *1.68; $\tau_{h*,CaHVA}$: *0.6	c = 0.144	c = 0.381
	$V_{offm*,CaHVA}$: -4.9 mV; $V_{offh*,CaHVA}$: -5.1 mV; $\tau_{m*,CaHVA}$: *0.6; $\tau_{h*,CaHVA}$: *1.66	c = 0.189	c = 0.464
	$V_{offm*,CaHVA}$: +4.9 mV; $V_{offh*,CaHVA}$: -5.1 mV; $\tau_{m*,CaHVA}$: *0.6; $\tau_{h*,CaHVA}$: *1.66	c = 0.342	c = 0.312 $\times$
	$V_{offm*,CaHVA}$: -4.9 mV; $V_{offh*,CaHVA}$: +5.1 mV; $\tau_{m*,CaHVA}$: *0.6; $\tau_{h*,CaHVA}$: *1.66	c = 0.122	c = 0.516
	$V_{offm*,CaHVA}$: +4.9 mV; $V_{offh*,CaHVA}$: +5.1 mV; $\tau_{m*,CaHVA}$: *0.6; $\tau_{h*,CaHVA}$: *1.66	c > 2.000	c = 0.418
	$V_{offm*,CaHVA}$: -4.9 mV; $V_{offh*,CaHVA}$: -5.1 mV; $\tau_{m*,CaHVA}$: *1.68; $\tau_{h*,CaHVA}$: *1.66	c = 0.848	c = 0.422
	$V_{offm*,CaHVA}$: +4.9 mV; $V_{offh*,CaHVA}$: -5.1 mV; $\tau_{m*,CaHVA}$: *1.68; $\tau_{h*,CaHVA}$: *1.66	c = 0.113	c = 0.412
	$V_{offm*,CaHVA}$: -4.9 mV; $V_{offh*,CaHVA}$: +5.1 mV; $\tau_{m*,CaHVA}$: *1.68; $\tau_{h*,CaHVA}$: *1.66	c = 0.320	c = 0.396
	$V_{offm*,CaHVA}$: +4.9 mV; $V_{offh*,CaHVA}$: +5.1 mV; $\tau_{m*,CaHVA}$: *1.68; $\tau_{h*,CaHVA}$: *1.66	c = 0.157	c = 0.363
CACNB2 [16]	$\tau_{h*,CaHVA}$: *1.26	c > 2.000	c > 2.000
CACNA1I [17]	$V_{offma,CaLVA}$: +1.3 mV; $V_{offha,CaLVA}$: +1.6 mV; $\tau_{m*,CaLVA}$: *0.87; $\tau_{h*,CaLVA}$: *0.8	c = 1.925	c = 1.875
	$V_{offma,CaLVA}$: +1.3 mV; $V_{offha,CaLVA}$: +1.6 mV; $\tau_{m*,CaLVA}$: *1.45; $\tau_{h*,CaLVA}$: *0.8	c > 2.000	c = 0.693
CACNA1I [18]	$V_{offma,CaLVA}$: -4.3 mV; $V_{sloma,CaLVA}$: *1.14; $V_{offha,CaLVA}$: -4.4 mV; $V_{sloha,CaLVA}$: *0.89; $\tau_{m*,CaLVA}$: *0.53; $\tau_{h*,CaLVA}$: *0.46	c = 1.040	c = 0.633 $\times$
	$V_{offma,CaLVA}$: -4.3 mV; $V_{sloma,CaLVA}$: *1.14; $V_{offha,CaLVA}$: -4.4 mV; $V_{sloha,CaLVA}$: *1.04; $\tau_{m*,CaLVA}$: *0.53; $\tau_{h*,CaLVA}$: *0.46	c = 0.846	c = 0.345
ATP2A2 [19]	$\gamma_{[Ca^{2+}]}$: *0.6	c = 0.179	c = 0.189 $\star$ $\times$
ATP2B2 [20]	$\tau_{[Ca^{2+}]}$: *1.97	c = 0.149	c = 0.305 $\star$
ATP2B2 [21]	$\tau_{[Ca^{2+}]}$: *1.5; $c_{min,[Ca^{2+}]}$: *1.4	c = 0.137	c = 0.484
ATP2B2 [22]	$\tau_{[Ca^{2+}]}$: *4.45	c = 0.067	c = 0.136
ATP2B2 [23]	$c_{min,[Ca^{2+}]}$: *1.1	c = 0.664	c > 2.000
SCN1A [24]	$V_{offm,Nat}$: -0.3 mV; $V_{offh,Nat}$: +5 mV; $V_{slom,Nat}$: *1.15; $V_{sloh,Nat}$: *1.23	c = 0.036	c = 0.005
SCN1A [25]	$V_{offm,Nat}$: +2.8 mV; $V_{offh,Nat}$: +9.6 mV; $V_{slom,Nat}$: *0.984; $V_{sloh,Nat}$: *1.042	c = 0.118	c = 0.015
SCN1A [26]	$V_{offm,Nat}$: -4.0 mV; $V_{offh,Nat}$: -5.8 mV; $V_{slom,Nat}$: *0.92; $V_{sloh,Nat}$: *1.13; $\tau_{h*,Nat}$: *1.47	c = 0.133	c = 0.223 $\star$
SCN1A [26]	$V_{offm,Nat}$: -8.1 mV; $V_{offh,Nat}$: +2.2 mV; $V_{slom,Nat}$: *0.97; $V_{sloh,Nat}$: *0.97; $\tau_{h*,Nat}$: *1.59	c = 0.023	c = 0.004
SCN1A [27]	$V_{offm,Nat}$: +6.0 mV; $V_{slom,Nat}$: *1.16; $\tau_{h*,Nat}$: *1.29	c = 0.049	c = 0.005
SCN1A [28]	$V_{offm,Nat}$: +10.0 mV; $V_{offh,Nat}$: -0.6 mV; $V_{slom,Nat}$: *1.15; $V_{sloh,Nat}$: *1.14	c = 0.023	c = 0.003 $\times$
HCN1 [29]	$V_{offm*,h}$: -26.5 mV; $V_{slom*,h}$: *0.64	c = 0.079	c = 0.008
HCN1 [30]	$V_{offm*,h}$: -25.9 mV; $V_{slom*,h}$: *0.6	c = 0.067	c = 0.007
	$V_{offm*,h}$: +17.7 mV; $V_{slom*,h}$: *0.6	c = 0.177	c = 0.092 $\star$
KCNB1 [31]	$V_{offm,Kp}$: +5 mV; $V_{offh,Kp}$: +3 mV; $V_{slom,Kp}$: *1.11; $V_{sloh,Kp}$: *0.86; $\tau_{m*,Kp}$: *0.5; $\tau_{h*,Kp}$: *0.53	c > 2.000	c = 0.171 $\star$ $\times$
KCNB1 [31]	$V_{offm,Kp}$: +1 mV; $V_{offh,Kp}$: -6 mV; $V_{slom,Kp}$: *1.22; $V_{sloh,Kp}$: *1.0; $\tau_{m*,Kp}$: *0.89; $\tau_{h*,Kp}$: *1.13	c > 2.000	c = 0.049
KCNB1 [31]	$V_{offm,Kp}$: +6 mV; $V_{offh,Kp}$: -8 mV; $V_{slom,Kp}$: *1.33; $V_{sloh,Kp}$: *1.0; $\tau_{m*,Kp}$: *0.5; $\tau_{h*,Kp}$: *0.87	c > 2.000	c = 0.047
KCNB1 [31]	$V_{offm,Kp}$: -28 mV; $V_{offh,Kp}$: -27 mV; $V_{slom,Kp}$: *1.11; $V_{sloh,Kp}$: *0.71; $\tau_{m*,Kp}$: *1.13; $\tau_{h*,Kp}$: *2.27	c > 2.000	c = 0.024
KCNB1 [31]	$V_{offm,Kp}$: +14 mV; $V_{offh,Kp}$: -21 mV; $V_{slom,Kp}$: *2.0; $V_{sloh,Kp}$: *1.0; $\tau_{m*,Kp}$: *0.39; $\tau_{h*,Kp}$: *1.2	c > 2.000	c = 0.021
KCNB1 [31]	$V_{offm,Kp}$: -13 mV; $V_{offh,Kp}$: -13 mV; $V_{slom,Kp}$: *1.33; $V_{sloh,Kp}$: *0.71; $\tau_{m*,Kp}$: *0.95; $\tau_{h*,Kp}$: *5.13	c > 2.000	c = 0.026
KCNMA1 [32]	$V_{offm,BK}$: -13.8 mV; $\tau_{m*,BK}$: *0.51	c = N/A	c = 1.795
	$V_{offm,BK}$: +57.0 mV; $\tau_{m*,BK}$: *0.51	c = N/A	c > 2.000
	$V_{offm,BK}$: -13.8 mV; $\tau_{m*,BK}$: *2.75	c = N/A	c = 1.841
	$V_{offm,BK}$: +57.0 mV; $\tau_{m*,BK}$: *2.75	c = N/A	c > 2.000
KCNMA1 [33]	$V_{offm,BK}$: +49.2 mV	c = N/A	c > 2.000
KCNMA1 [34]	$V_{offm,BK}$: +16 mV; $\tau_{m*,BK}$: *5.91	c = N/A	c > 2.000
KCNMA1 [35]	$V_{offm,BK}$: -5 mV; $\tau_{m*,BK}$: *7.59	c = N/A	c > 2.000
	$V_{offm,BK}$: +15 mV; $\tau_{m*,BK}$: *7.59	c = N/A	c > 2.000
KCNMA1 [36]	$V_{offm,BK}$: -58 mV	c = N/A	c = 0.430

References

[1] Michaela Kudrnac, Stanislav Beyl, Annette Hohaus, Anna Stary, Thomas Peterbauer, Eugen Timin, and Steffen Hering. Coupled and independent contributions of residues in IS6 and IIS6 to activation gating of CaV1.2. *J Biol Chem*, 284(18):12276–12284, 2009.

[2] Katrin Depil, Stanislav Beyl, Anna Stary-Weinzinger, Annette Hohaus, Eugen Timin, and Steffen Hering. Timothy mutation disrupts the link between activation and inactivation in cav1. 2 protein. *J Biol Chem*, 286(36):31557–31564, 2011.

[3] Annette Hohaus, Stanislav Beyl, Michaela Kudrnac, Stanislav Berjukow, Eugen N Timin, Rainer Marksteiner, Marion A Maw, and Steffen Hering. Structural determinants of l-type channel activation in segment iis6 revealed by a retinal disorder. *J Biol Chem*, 280(46):38471–38477, 2005.

[4] Anna Stary, Michaela Kudrnac, Stanislav Beyl, Annette Hohaus, Eugen Timin, Peter Wolschann, H Robert Guy, and Steffen Hering. Molecular dynamics and mutational analysis of a channelopathy mutation in the iis6 helix of cav1. 2. *Channels*, 2(3):216–223, 2008.

[5] Zhen Zhi Tang, Mui Cheng Liang, Songqing Lu, Dejie Yu, Chye Yun Yu, David T Yue, and Tuck Wah Soong. Transcript scanning reveals novel and extensive splice variations in human l-type voltage-gated calcium channel, cav1. 2 α1 subunit. *J Biol Chem*, 279(43):44335–44343, 2004.

[6] Bao Zhen Tan, Fengli Jiang, Ming Yeong Tan, Dejie Yu, Hua Huang, Yiru Shen, and Tuck Wah Soong. Functional characterization of alternative splicing in the c terminus of l-type cav1. 3 channels. *J Biol Chem*, 286(49):42725–42735, 2011.

[7] Gabriella Bock, Mathias Gebhart, Anja Scharinger, Wanchana Jangsangthong, Perrine Busquet, Chiara Poggiani, Simone Sartori, Matteo E Mangoni, Martina J Sinnegger-Brauns, Stefan Herzig, et al. Functional properties of a newly identified c-terminal splice variant of cav1. 3 l-type ca2+ channels. *J Biol Chem*, 286(49):42736–42748, 2011.

[8] Q. Zhang, V. Timofeyev, H. Qiu, L. Lu, N. Li, A. Singapuri, C. L. Torado, H. S. Shin, and N. Chiamvimonvat. Expression and roles of Cav1.3 (α1D) L-type Ca2+ channel in atrioventricular node automaticity. *J Mol Cell Cardiol*, 50(1):194–202, 2011.

[9] Alberto Pérez-Alvarez, Alicia Hernández-Vivanco, Jose Carlos Caba-González, and Almudena Albillos. Different roles attributed to cav1 channel subtypes in spontaneous action potential firing and fine tuning of exocytosis in mouse chromaffin cells. *J Neurochem*, 116(1):105–121, 2011.

[10] Alexandra Pinggera, Andreas Lieb, Bruno Benedetti, Michaela Lampert, Stefania Monteleone, Klaus R Liedl, Petronel Tuluc, and Jörg Striessnig. Cacna1d de novo mutations in autism spectrum disorders activate cav1. 3 l-type calcium channels. *Biol Psychiatry*, 77(9):816–822, 2015.

[11] Elena AB Azizan, Hanne Poulsen, Petronel Tuluc, Junhua Zhou, Michael V Clausen, Andreas Lieb, Carmela Maniero, Sumedha Garg, Elena G Bochukova, Wanfeng Zhao, et al. Somatic mutations in atp1a1 and cacna1d underlie a common subtype of adrenal hypertension. *Nat Genet*, 45(9):1055–1060, 2013.

[12] Andreas Lieb, Anja Scharinger, Simone Sartori, Martina J Sinnegger-Brauns, and Jörg Striessnig. Structural determinants of cav1. 3 l-type calcium channel gating. *Channels*, 6(3):197–205, 2012.

[13] Jonathan M Cordeiro, Mark Marieb, Ryan Pfeiffer, Kirstine Calloe, Elena Burashnikov, and Charles Antzelevitch. Accelerated inactivation of the L-type calcium current due to a mutation in CACNB2b underlies brugada syndrome. *J Mol Cell Cardiol*, 46(5):695–703, 2009.

[14] Enrique Massa, Kevin M Kelly, David I Yule, Robert L MacDonald, and Michael D Uhler. Comparison of fura-2 imaging and electrophysiological analysis of murine calcium channel alpha 1 subunits coexpressed with novel beta 2 subunit isoforms. *Mol Pharmacol*, 47(4):707–716, 1995.

[15] Sabine Link, Marcel Meissner, Brigitte Held, Andreas Beck, Petra Weissgerber, Marc Freichel, and Veit Flockerzi. Diversity and developmental expression of L-type calcium channel β2 proteins and their influence on calcium current in murine heart. *J Biol Chem*, 284(44):30129–30137, 2009.

[16] Dan Hu, Hector Barajas-Martinez, Vladislav V Nesterenko, Ryan Pfeiffer, Alejandra Guerchicoff, Jonathan M Cordeiro, Anne B Curtis, Guido D Pollevick, Yuesheng Wu, Elena Burashnikov, et al. Dual variation in scn5a and cacnb2b underlies the development of cardiac conduction disease without brugada syndrome. *Pacing Clin Electrophysiol*, 33(3):274–285, 2010.

[17] Janet Murbartián, Juan Manuel Arias, and Edward Perez-Reyes. Functional impact of alternative splicing of human T-type Cav3.3 calcium channels. *J Neurophysiol*, 92(6):3399–3407, 2004.

[18] Juan Carlos Gomora, Janet Murbartian, Juan Manuel Arias, Jung-Ha Lee, and Edward Perez-Reyes. Cloning and expression of the human t-type channel ca v 3.3: insights into prepulse facilitation. *Biophys J*, 83(1):229–241, 2002.

[19] Yong Ji, M Jane Lalli, Gopal J Babu, Yanfang Xu, Darryl L Kirkpatrick, Lynne H Liu, Nipavan Chiamvimonvat, Richard A Walsh, Gary E Shull, and Muthu Periasamy. Disruption of a single copy of the serca2 gene results in altered ca2+ homeostasis and cardiomyocyte function. *J Biol Chem*, 275(48):38073–38080, 2000.

[20] Amanda K Fakira, Lawrence D Gaspers, Andrew P Thomas, Hong Li, Mohit R Jain, and Stella Elkabes. Purkinje cell dysfunction and delayed death in plasma membrane calcium atpase 2-heterozygous mice. *Mol Cell Neurosci*, 51(1):22–31, 2012.

[21] Ruth M Empson, Walther Akemann, and Thomas Knöpfel. The role of the calcium transporter protein plasma membrane calcium ATPase PMCA2 in cerebellar Purkinje neuron function. *Funct Neurol*, 25(3):153, 2010.

[22] R Ficarella, F Di Leva, M Bortolozzi, S Ortolano, F Donaudy, M Petrillo, S Melchionda, A Lelli, T Domi, L Fedrizzi, et al. A functional study of plasma-membrane calcium-pump isoform 2 mutants causing digenic deafness. *Proc Natl Acad Sci USA*, 104(5):1516–1521, 2007.

[23] Marta Giacomello, Agnese De Mario, Raffaele Lopreiato, Simona Primerano, Mara Campeol, Marisa Brini, and Ernesto Carafoli. Mutations in pmca2 and hereditary deafness: a molecular analysis of the pump defect. *Cell Calcium*, 50(6):569–576, 2011.

[24] Sandrine Cestèle, Paolo Scalmani, Raffaella Rusconi, Benedetta Terragni, Silvana Franceschetti, and Massimo Mantegazza. Self-limited hyperexcitability: Functional effect of a familial hemiplegic migraine mutation of the Nav1.1 (SCN1A) na+ channel. *J Neurosci*, 28(29):7273–7283, 2008.

[25] Kaate RJ Vanmolkot, Elena Babini, Boukje de Vries, Anine H Stam, Tobias Freilinger, Gisela M Terwindt, Lisa Norris, Joost Haan, Rune R Frants, Nabih M Ramadan, et al. The novel p.L1649Q mutation in the SCN1A epilepsy gene is associated with familial hemiplegic migraine: genetic and functional studies. *Hum Mutat*, 28(5):522–522, 2007.

[26] Linda Volkers, Kristopher M Kahlig, Nienke E Verbeek, Joost HG Das, Marjan JA van Kempen, Hans Stroink, Paul Augustijn, Onno van Nieuwenhuizen, Dick Lindhout, Alfred L George, et al. Nav1. 1 dysfunction in genetic epilepsy with febrile seizures-plus or dravet syndrome. *European J Neurosci*, 34(8):1268–1275, 2011.

[27] Sandrine Cestèle, Angelo Labate, Raffaella Rusconi, Patrizia Tarantino, Laura Mumoli, Silvana Franceschetti, Grazia Annesi, Massimo Mantegazza, and Antonio Gambardella. Divergent effects of the t1174s scn1a mutation associated with seizures and hemiplegic migraine. *Epilepsia*, 54(5):927–935, 2013.

[28] Massimo Mantegazza, Antonio Gambardella, Raffaella Rusconi, Emanuele Schiavon, Ferdinanda Annesi, Rita Restano Cassulini, Angelo Labate, Sara Carrideo, Rosanna Chifari, Maria Paola Canevini, et al. Identification of an nav1.1 sodium channel (scn1a) loss-of-function mutation associated with familial simple febrile seizures. *Proc Natl Acad Sci USA of the United States of America*, 102(50):18177–18182, 2005.

[29] Takahiro M Ishii, Noriyuki Nakashima, and Harunori Ohmori. Tryptophan-scanning mutagenesis in the s1 domain of mammalian hcn channel reveals residues critical for voltage-gated activation. *J Physiol (Lond)*, 579(2):291–301, 2007.

[30] Heinte Lesso and Ronald A Li. Helical secondary structure of the external s3-s4 linker of pacemaker (hcn) channels revealed by site-dependent perturbations of activation phenotype. *J Biol Chem*, 278(25):22290–22297, 2003.

[31] E Bocksteins, N Ottschytsch, J-P Timmermans, AJ Labro, and DJ Snyders. Functional interactions between residues in the S1, S4, and S5 domains of Kv2.1. *Eur Biophys J*, 40(6):783–793, 2011.

[32] Jian-Zhong Sheng, Aalim Weljie, Lusia Sy, Shizhang Ling, Hans J Vogel, and Andrew P Braun. Homology modeling identifies c-terminal residues that contribute to the ca2+ sensitivity of a bkca channel. *Biophys J*, 89(5):3079–3092, 2005.

[33] Gonzalo Budelli, Yanyan Geng, Alice Butler, Karl L Magleby, and Lawrence Salkoff. Properties of slo1 k+ channels with and without the gating ring. *Proc Natl Acad Sci USA*, page 201313433, 2013.

[34] Xiaojin Liu, Yongchan Chang, Peter H Reinhart, and Harald Sontheimer. Cloning and characterization of glioma bk, a novel bk channel isoform highly expressed in human glioma cells. *J Neurosci*, 22(5):1840–1849, 2002.

[35] Malle Soom, Guido Gessner, Heike Heuer, Toshinori Hoshi, and Stefan H Heinemann. A mutually exclusive alternative exon of slo 1 codes for a neuronal bk channel with altered function. *Channels*, 2(4):278–282, 2008.

[36] Lie Chen, Lijun Tian, Stephen H-F MacDonald, Heather McClafferty, Martin SL Hammond, Jean-Marc Huibant, Peter Ruth, Hans-Guenther Knaus, and Michael J Shipston. Functionally diverse complement of large conductance calcium-and voltage-activated potassium channel (bk) α-subunits generated from a single site of splicing. *J Biol Chem*, 280(39):33599–33609, 2005.
