# Supplementary material for: Computational Modeling of Genetic Contributions to Excitability and Neural Coding in Layer V Pyramidal Cells: Applications to Schizophrenia Pathology
Source: Front Comput Neurosci. 2019 Sep 26;13:66. doi: 10.3389/fncom.2019.00066 (PMC6775251; doi:10.3389/fncom.2019.00066)
Supplement: Supplementary file 2 [file Data_Sheet_2.pdf]

Computational modeling of genetic contributions to excitability and neural coding in layer V pyramidal cells: applications to schizophrenia pathology. Supplementary material.

Table S1: **Control neuron firing behaviour in each model.** The first column shows the name of the model, and the second to fifth columns show the threshold amplitudes for inducing an action potential as a response to the stimuli of conditions I – III. In condition III, the proportion of amplitudes A_{3a} and A_{3b} was kept fixed as $A_{3a}/A_{3b} = A_1/A_2$ in order to restrict to a single threshold value. The sixth column shows the number of action potentials as a response to a short (5 ms) somatic square pulse of amplitude $1.15A_1$. The seventh and eighth column show the membrane potential at the distal (at a distance of 800 µm from soma) apical dendrite at rest and shortly (4 ms) after the first spike induced by this stimulus. The considered interval, 4 ms, was approximately half of the inter-spike interval in bursts of the Almog models and altered Hay models. In Hay- and Almog-model neurons, the difference between membrane potentials at rest and 4 ms after a spike was small. By contrast, in the altered Hay-model neurons, the membrane potential quickly rose to a value near the Na^+ reversal potential after a spike, initiating a dendritic action potential which then propagates to soma and induced further spiking.

	A_1 (nA)	A_2 (µS)	A_{3a} (nA)	A_{3b} (µS)	No. spikes (cond. I)	Apical V_m at rest	Apical V_m 4 ms after spike
Hay	1.32	0.0346	0.829	0.0218	1	-70.1	-62.6
Hay-A_1	1.32	0.0182	1.07	0.0148	2	-70.1	15.0
Hay-A_2	1.32	0.0150	1.09	0.0124	2	-70.1	28.1
Hay-A_3	1.32	0.0150	1.09	0.0124	3	-70.1	28.1
Hay-A_4	1.32	0.0150	1.09	0.0124	3	-70.1	28.1
Hay-A_5	1.32	0.0150	1.09	0.0124	3	-70.1	28.1
Hay-A_6	1.32	0.0150	1.09	0.0124	3	-70.1	28.1
Almog	0.462	0.0179	0.389	0.0151	1	-58.3	-44.7
Almog-A_1	0.462	0.0179	0.389	0.0151	1	-58.3	-44.7
Almog-A_2	0.462	0.0179	0.390	0.0151	1	-58.3	-44.7
Almog-A_3	0.462	0.0179	0.390	0.0151	1	-58.3	-44.7
Almog-A_4	0.462	0.0179	0.390	0.0151	1	-58.3	-44.7
Almog-A_5	0.462	0.0174	0.390	0.0151	1	-58.2	-40.7
Almog-A_6	0.462	0.0168	0.389	0.0151	1	-58.2	-32.5

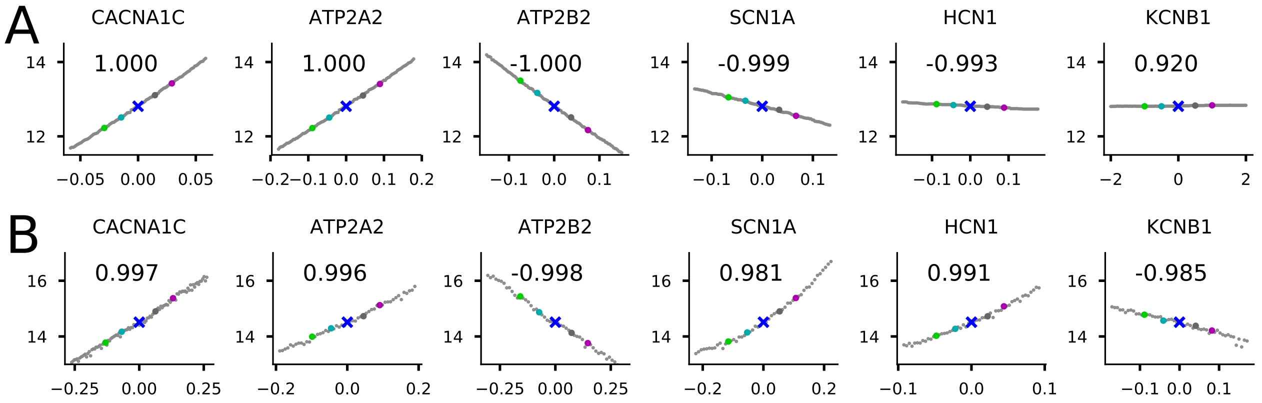

Figure S1: **The effects of the model variants are relatively linear with respect to the scaling coefficient.** The y-axis shows the f-I curve averages (spikes/s) with respect to the scaling coefficient in the Hay (A) or Almog (B) model. The x-axis shows the scaling coefficients in a range $(-c, c)$, where c is the threshold scaling parameter corresponding to the variant of Figure 2. 101 (A) or 51 (B) data points were considered (light gray dots), and the Pearson correlation coefficient of these data are displayed above the data, values close to ±1 indicating a linear behaviour. The purple, dark gray, blue, cyan, and green data points correspond to the $\epsilon = \frac{1}{2}$, $\frac{1}{4}$, 0 (control), -$\frac{1}{4}$, and -$\frac{1}{2}$ variants and their f-I curve averages (see Figure 2E), respectively.

Table S2: **Table of the scaling coefficient parameters in unaltered and altered Hay and Almog models.** An expansion of Table A2, including altered models. The table entries are ordered as in Table A2. In most cases (1304/1463 table entries, i.e., 89%) a degree of scaling was performed (scaling coefficient < 2.0), while in 11% of the cases none of the scaling conditions were violated by the unscaled variant (this happened for at least one of the 14 models in 24 out of 109 model variants).

Gene	Hay	Hay-A_1	Hay-A_2	Hay-A_3	Hay-A_4	Hay-A_5	Hay-A_6	Almog	Almog-A_1	Almog-A_2	Almog-A_3	Almog-A_4	Almog-A_5	Almog-A_6
CACNA1C [1]	0.066	0.054	0.057	0.052	0.061	0.051	0.052	0.095	0.086	0.027	0.123	0.019	0.085	0.073
CACNA1C [1]	0.042	0.033	0.037	0.033	0.039	0.033	0.030	0.059	0.063	0.025	0.088	0.016	0.060	0.050
CACNA1C [2]	0.043	0.034	0.037	0.033	0.038	0.033	0.031	0.070	0.062	0.031	0.100	0.015	0.071	0.040
	0.101	0.097	0.066	0.110	0.109	0.135	0.123	0.222	0.193	0.354	0.101	0.560	0.508	0.078
	0.049	0.039	0.045	0.041	0.048	0.039	0.038	0.074	0.068	0.022	0.098	0.015	0.068	0.063
	0.076	0.070	0.043	0.073	0.072	0.090	0.119	0.268	0.209	0.450	0.125	0.759	0.745	0.116
	0.031	0.025	0.027	0.024	0.028	0.025	0.027	0.065	0.070	0.023	0.099	0.013	0.070	0.063
	0.208	1.024	0.885	0.855	0.692	0.558	0.281	0.263	0.168	0.328	0.133	0.543	0.475	0.085
	0.034	0.026	0.032	0.028	0.032	0.028	0.025	0.067	0.062	0.031	0.098	0.015	0.067	0.044
	0.290	0.497	0.201	0.325	0.307	0.496	0.392	0.269	0.250	0.486	0.157	0.813	0.865	0.115
	0.058	0.048	0.049	0.045	0.052	0.046	0.041	0.070	0.063	0.032	0.101	0.014	0.070	0.039
	0.059	0.057	0.036	0.060	0.059	0.076	0.071	0.262	0.196	0.344	0.100	0.547	0.500	0.098
	0.071	0.060	0.066	0.060	0.071	0.059	0.067	0.078	0.065	0.031	0.100	0.014	0.067	0.035
	0.049	0.046	0.028	0.052	0.047	0.059	0.055	0.258	0.218	0.455	0.164	0.726	0.719	0.123
	0.038	0.031	0.033	0.030	0.034	0.031	0.026	0.077	0.058	0.032	0.101	0.015	0.068	0.059
	0.176	0.175	0.113	0.180	0.158	0.234	0.216	0.259	0.211	0.343	0.114	0.597	0.523	0.086
	0.038	0.035	0.040	0.035	0.041	0.037	0.031	0.067	0.068	0.023	0.098	0.014	0.066	0.035
	0.113	0.105	0.060	0.099	0.090	0.129	0.129	0.304	0.229	0.526	0.142	0.823	0.945	0.124
CACNA1C [3]	0.028	0.023	0.024	0.022	0.024	0.022	0.018	0.050	0.053	0.021	0.084	0.013	0.058	0.052
	0.123	0.129	0.103	0.156	0.143	0.205	0.109	0.143	0.100	0.188	0.065	0.293	0.282	0.046
	0.035	0.027	0.033	0.029	0.034	0.030	0.027	0.064	0.056	0.026	0.081	0.012	0.055	0.029
	0.063	0.059	0.035	0.063	0.057	0.076	0.073	0.165	0.134	0.250	0.068	0.407	0.340	0.057
CACNA1C [4]	0.052	0.043	0.039	0.041	0.047	0.041	0.036	0.054	0.072	0.036	0.117	0.017	0.079	0.061
	0.077	0.074	0.047	0.080	0.081	0.100	0.134	0.220	0.159	0.269	0.089	0.473	0.382	0.072
	0.057	0.047	0.051	0.046	0.053	0.045	0.047	0.078	0.073	0.036	0.112	0.017	0.078	0.039
	0.069	0.065	0.040	0.069	0.069	0.085	0.080	0.251	0.188	0.292	0.118	0.517	0.462	0.073
	0.042	0.031	0.037	0.033	0.038	0.034	0.030	0.079	0.078	0.036	0.113	0.017	0.079	0.072
	0.145	0.140	0.090	0.148	0.138	0.195	0.181	0.252	0.140	0.277	0.125	0.469	0.422	0.079
	0.044	0.035	0.040	0.036	0.041	0.037	0.033	0.062	0.072	0.036	0.113	0.018	0.077	0.073
	0.119	0.112	0.068	0.113	0.104	0.149	0.148	0.183	0.175	0.305	0.108	0.508	0.422	0.078
CACNA1C [5]	0.157	0.130	0.139	0.127	0.146	0.127	0.127	0.188	0.175	0.088	0.270	0.042	0.191	0.109
	0.236	0.230	0.143	0.235	0.219	0.293	0.279	1.625	1.416	2.000	0.824	2.000	2.000	0.747
CACNA1D [6], [7]	0.083	0.069	0.069	0.062	0.071	0.064	0.057	0.183	0.195	0.096	0.300	0.045	0.211	0.133
	0.075	0.062	0.063	0.056	0.065	0.058	0.063	0.187	0.179	0.084	0.295	0.047	0.203	0.220
CACNA1D [6], [7]	0.080	0.067	0.068	0.061	0.071	0.063	0.056	0.221	0.195	0.101	0.318	0.049	0.225	0.139
	0.424	1.057	0.618	0.540	0.640	0.525	0.498	0.502	0.344	0.636	0.205	1.004	0.839	0.157
	0.094	0.073	0.088	0.077	0.090	0.075	0.086	0.215	0.198	0.092	0.306	0.047	0.209	0.117
	0.663	0.769	0.500	0.749	0.600	0.980	0.581	0.506	0.500	0.702	0.247	1.139	1.072	0.205
	0.072	0.061	0.062	0.056	0.064	0.057	0.059	0.213	0.198	0.085	0.314	0.047	0.221	0.259
	0.274	0.355	0.324	0.280	0.324	0.268	0.312	0.500	0.376	0.602	0.207	0.968	0.859	0.156
	0.083	0.069	0.078	0.068	0.080	0.069	0.073	0.212	0.213	0.095	0.306	0.039	0.210	0.111
	0.341	1.910	1.188	1.411	1.468	1.334	0.678	0.625	0.391	0.715	0.282	1.094	1.051	0.194
CACNA1D [8], [9]	0.190	0.191	0.138	0.224	0.198	0.284	0.201	0.283	0.198	0.353	0.102	0.570	0.522	0.092
	0.123	0.115	0.069	0.119	0.111	0.151	0.147	0.242	0.250	0.402	0.157	0.686	0.610	0.106
	0.209	0.210	0.153	0.263	0.224	0.314	0.223	0.244	0.219	0.377	0.112	0.606	0.563	0.103
	0.130	0.122	0.073	0.121	0.118	0.159	0.153	0.246	0.202	0.516	0.157	0.719	0.663	0.117
CACNA1D [10]	0.181	0.146	0.139	0.131	0.153	0.133	0.111	0.225	0.211	0.062	0.327	0.055	0.235	0.250
CACNA1D [11]	0.045	0.036	0.038	0.033	0.040	0.035	0.032	0.083	0.082	0.040	0.127	0.020	0.088	0.055
	0.318	0.334	0.294	0.485	0.453	0.585	0.266	0.259	0.219	0.344	0.115	0.532	0.501	0.105
	0.053	0.041	0.048	0.040	0.050	0.043	0.047	0.088	0.080	0.040	0.125	0.020	0.086	0.068
	0.152	0.137	0.082	0.149	0.137	0.181	0.163	0.263	0.266	0.397	0.141	0.610	0.562	0.135
	0.059	0.046	0.049	0.043	0.052	0.046	0.038	0.095	0.081	0.030	0.127	0.018	0.089	0.053
	0.105	0.105	0.069	0.123	0.113	0.148	0.143	0.282	0.201	0.379	0.121	0.538	0.509	0.093
	0.074	0.058	0.067	0.058	0.072	0.062	0.059	0.096	0.080	0.041	0.121	0.020	0.084	0.046
	0.076	0.072	0.042	0.070	0.074	0.091	0.086	0.313	0.249	0.438	0.165	0.621	0.600	0.149
CACNA1D [12]	0.065	0.054	0.056	0.050	0.057	0.052	0.052	0.126	0.109	0.053	0.181	0.027	0.127	0.125
	0.063	0.051	0.055	0.049	0.057	0.051	0.043	0.116	0.113	0.053	0.176	0.028	0.124	0.089
CACNB2 [13]	0.371	0.374	0.393	0.323	0.401	0.306	0.333	2.000	1.871	2.000	1.766	2.000	2.000	1.402
CACNB2 [14]	2.000	2.000	2.000	2.000	2.000	2.000	1.589	2.000	2.000	2.000	2.000	2.000	2.000	2.000
CACNB2 [15]	0.194	0.176	0.263	0.214	0.291	0.207	0.221	0.537	0.512	0.235	0.805	0.114	0.550	0.337
	0.278	0.231	0.127	0.203	0.185	0.244	0.264	0.364	0.236	0.474	0.151	0.802	0.679	0.129
	0.131	0.117	0.164	0.141	0.172	0.126	0.139	0.569	0.520	0.230	0.776	0.107	0.534	0.404
	1.153	0.902	0.239	0.389	0.343	0.540	0.308	0.409	0.283	0.476	0.176	0.817	0.656	0.119
	1.201	0.741	0.500	0.483	0.584	0.558	0.618	0.449	0.408	0.207	0.633	0.090	0.439	0.367
	0.105	0.105	0.074	0.125	0.109	0.161	0.156	0.384	0.232	0.544	0.197	0.780	0.750	0.156
	0.373	0.251	0.226	0.224	0.227	0.232	0.219	0.453	0.438	0.204	0.635	0.095	0.435	0.234
	0.144	0.145	0.104	0.185	0.159	0.234	0.257	0.381	0.302	0.477	0.168	0.805	0.723	0.156
	0.189	0.157	0.224	0.182	0.248	0.191	0.207	0.464	0.432	0.184	0.703	0.091	0.476	0.252
	0.342	0.276	0.158	0.236	0.212	0.282	0.273	0.312	0.376	0.509	0.176	0.832	0.703	0.141
	0.122	0.109	0.151	0.123	0.157	0.122	0.125	0.516	0.469	0.154	0.698	0.102	0.473	0.224
	2.000	2.000	0.386	0.492	0.493	0.693	0.376	0.418	0.249	0.567	0.174	0.812	0.728	0.132
	0.848	0.531	0.396	0.365	0.454	0.414	0.373	0.422	0.406	0.125	0.570	0.091	0.394	0.260
	0.113	0.112	0.078	0.129	0.125	0.175	0.169	0.412	0.319	0.517	0.174	0.808	0.743	0.148
	0.320	0.216	0.205	0.185	0.206	0.202	0.164	0.396	0.351	0.186	0.571	0.088	0.395	0.249
	0.157	0.160	0.116	0.192	0.171	0.268	0.288	0.363	0.321	0.598	0.200	0.857	0.756	0.167
CACNB2 [16]	2.000	2.000	2.000	2.000	2.000	2.000	2.000	2.000	2.000	2.000	2.000	2.000	2.000	2.000
CACNA1I [17]	1.925	2.000	2.000	2.000	2.000	2.000	2.000	1.875	1.992	0.865	2.000	0.386	1.874	1.500
	2.000	2.000	2.000	2.000	2.000	2.000	2.000	0.693	0.625	1.310	0.353	1.997	1.379	0.292
CACNA1I [18]	1.040	2.000	2.000	2.000	2.000	2.000	0.641	0.633	0.655	0.282	2.000	0.128	0.556	0.469
	0.846	2.000	2.000	2.000	2.000	2.000	2.000	0.345	0.359	0.125	0.598	0.086	0.381	0.345
ATP2A2 [19]	0.179	0.199	0.117	0.188	0.158	0.217	0.211	0.189	0.167	0.250	0.115	0.550	0.577	0.141
ATP2B2 [20]	0.149	0.153	0.176	0.131	0.159	0.144	0.120	0.305	0.361	0.143	0.569	0.117	0.438	0.453
ATP2B2 [21]	0.137	0.138	0.181	0.147	0.150	0.151	0.119	0.484	0.565	0.282	0.948	0.211	0.719	0.625
ATP2B2 [22]	0.067	0.069	0.080	0.065	0.073	0.065	0.054	0.136	0.161	0.074	0.255	0.048	0.179	0.162
ATP2B2 [23]	0.664	1.121	1.418	1.411	1.357	1.194	0.930	2.000	2.000	2.000	2.000	2.000	2.000	2.000
SCN1A [24]	0.036	0.132	0.136	0.217	0.154	0.228	0.156	0.005	0.007	0.011	0.008	0.012	0.011	0.007
SCN1A [25]	0.118	0.330	0.779	0.762	0.591	0.535	0.361	0.015	0.028	0.038	0.028	0.055	0.036	0.030
SCN1A [26]	0.133	0.264	0.077	0.074	0.074	0.068	0.067	0.223	0.203	0.312	0.184	0.315	0.234	0.237
SCN1A [26]	0.023	0.081	0.125	0.125	0.125	0.125	0.111	0.004	0.004	0.007	0.006	0.007	0.007	0.004
SCN1A [27]	0.049	0.081	0.184	0.184	0.184	0.184	0.184	0.005	0.005	0.004	0.006	0.007	0.012	0.006
SCN1A [28]	0.023	0.044	0.106	0.106	0.106	0.106	0.086	0.003	0.002	0.002	0.003	0.003	0.005	0.003
HCN1 [29]	0.079	0.185	0.185	0.185	0.185	0.179	0.180	0.008	0.008	0.007	0.010	0.009	0.017	0.011
HCN1 [30]	0.067	0.164	0.164	0.164	0.164	0.164	0.164	0.007	0.008	0.007	0.008	0.010	0.017	0.011
	0.177	0.434	0.434	0.434	0.434	0.412	0.416	0.092	0.091	0.149	0.115	0.140	0.136	0.104
KCNB1 [31]	2.000	2.000	2.000	2.000	2.000	2.000	2.000	0.171	0.156	0.125	0.172	0.227	0.354	0.225
KCNB1 [31]	2.000	2.000	2.000	2.000	2.000	2.000	2.000	0.049	0.053	0.049	0.060	0.062	0.117	0.070
KCNB1 [31]	2.000	2.000	2.000	2.000	2.000	2.000	2.000	0.047	0.043	0.043	0.047	0.050	0.093	0.062
KCNB1 [31]	2.000	2.000	2.000	2.000	2.000	2.000	2.000	0.024	0.025	0.022	0.030	0.028	0.053	0.027
KCNB1 [31]	2.000	2.000	2.000	2.000	2.000	2.000	2.000	0.021	0.020	0.020	0.021	0.024	0.037	0.022
KCNB1 [31]	2.000	2.000	2.000	2.000	2.000	2.000	2.000	0.026	0.020	0.020	0.031	0.023	0.045	0.027
KCNMA1 [32]	N/A	N/A	N/A	N/A	N/A	N/A	N/A	1.795	1.847	1.650	2.000	1.728	2.000	2.000
	N/A	N/A	N/A	N/A	N/A	N/A	N/A	2.000	2.000	2.000	2.000	2.000	2.000	2.000
	N/A	N/A	N/A	N/A	N/A	N/A	N/A	1.841	1.898	1.683	2.000	1.750	2.000	2.000
	N/A	N/A	N/A	N/A	N/A	N/A	N/A	2.000	2.000	2.000	2.000	2.000	2.000	2.000
KCNMA1 [33]	N/A	N/A	N/A	N/A	N/A	N/A	N/A	2.000	2.000	2.000	2.000	2.000	2.000	2.000
KCNMA1 [34]	N/A	N/A	N/A	N/A	N/A	N/A	N/A	2.000	2.000	2.000	2.000	2.000	2.000	2.000
KCNMA1 [35]	N/A	N/A	N/A	N/A	N/A	N/A	N/A	2.000	2.000	2.000	2.000	2.000	2.000	2.000
	N/A	N/A	N/A	N/A	N/A	N/A	N/A	2.000	2.000	2.000	2.000	2.000	2.000	2.000
KCNMA1 [36]	N/A	N/A	N/A	N/A	N/A	N/A	N/A	0.430	0.441	0.406	0.481	0.410	0.501	0.538

Table S3: **Correlations between the integrals of f-I curves predicted by different models (data of Figure S3) across variants.** Mean ± STD of all correlations (excluding self-correlations): 0.81 ± 015. Mean ± STD of the correlations between Hay and Almog models: 0.69 ± 0.08.

	Hay	Hay-A$_1$	Hay-A$_2$	Hay-A$_3$	Hay-A$_4$	Hay-A$_5$	Hay-A$_6$	Almog	Almog-A$_1$	Almog-A$_2$	Almog-A$_3$	Almog-A$_4$	Almog-A$_5$	Almog-A$_6$
Hay	1.00	0.97	0.97	0.97	0.96	0.96	0.94	0.85	0.80	0.70	0.78	0.74	0.57	0.76
Hay-A$_1$		1.00	0.97	0.98	0.97	0.98	0.96	0.76	0.72	0.62	0.70	0.67	0.53	0.69
Hay-A$_2$			1.00	0.99	0.99	0.98	0.95	0.79	0.75	0.66	0.73	0.69	0.53	0.71
Hay-A$_3$				1.00	0.99	0.99	0.96	0.80	0.77	0.67	0.74	0.71	0.57	0.73
Hay-A$_4$					1.00	0.99	0.96	0.76	0.74	0.65	0.70	0.68	0.52	0.70
Hay-A$_5$						1.00	0.97	0.76	0.73	0.63	0.70	0.67	0.53	0.70
Hay-A$_6$							1.00	0.72	0.68	0.57	0.64	0.61	0.49	0.65
Almog								1.00	0.97	0.91	0.96	0.91	0.82	0.95
Almog-A$_1$									1.00	0.94	0.98	0.95	0.88	0.98
Almog-A$_2$										1.00	0.94	0.95	0.92	0.94
Almog-A$_3$											1.00	0.96	0.89	0.98
Almog-A$_4$												1.00	0.91	0.95
Almog-A$_5$													1.00	0.92
Almog-A$_6$														1.00

Table S4: **Stimulus amplitudes used for Hay (left) and Almog (right) models.** The first two columns show the amplitude of the somatic 5-ms square-pulse stimulus during the up and down states, respectively. The third column shows the amplitude of the proximal apical 600-ms square-pulse stimulus used for imitating the up state. The fourth column shows the maximal amplitude of the EPSP-like stimulus, injected at the apical dendrite, 600 or 850 μm from the soma.

	$A_{\text{soma up}}$ (nA)	$A_{\text{soma down}}$ (nA)	$A_{200\mu\text{m}}$ (nA)	$A_{\text{EPSP }600\mu\text{m}}$ (nA)
Hay	0.528	1.782	0.373	0.680
Hay-A$_1$	0.528	1.782	0.368	0.574
Hay-A$_2$	0.528	1.782	0.364	0.531
Hay-A$_3$	0.528	1.782	0.364	0.531
Hay-A$_4$	0.528	1.782	0.364	0.531
Hay-A$_5$	0.528	1.782	0.364	0.531
Hay-A$_6$	0.528	1.782	0.364	0.531

	$A_{\text{soma up}}$ (nA)	$A_{\text{soma down}}$ (nA)	$A_{400\mu\text{m}}$ (nA)	$A_{\text{EPSP }850\mu\text{m}}$ (nA)
Almog	0.185	0.624	0.769	0.600
Almog-A$_1$	0.185	0.624	0.769	0.599
Almog-A$_2$	0.185	0.624	0.769	0.600
Almog-A$_3$	0.185	0.624	0.769	0.601
Almog-A$_4$	0.185	0.624	0.769	0.601
Almog-A$_5$	0.185	0.624	0.768	0.518
Almog-A$_6$	0.185	0.624	0.767	0.432

Table S5: **A**: Threshold conductances with which 1000 excitatory conductance-based alpha synapses ($\tau = 5$ ms, $E_{rev} = 0$ mV) induced a spike in the control Almog model. The synapses were uniformly distributed across compartments in one of seven regions (1: 0-200 μm, 2: 200-400 μm, 3: 400-600 μm, 4: 600-800 μm, 5: 800-1000 μm, 6: > 1000 μm from the soma along the apical dendrite, or 7: in the basal dendrites). **B**: Correlations between the input and output patterns in the control Almog model neuron. The [Ca^{2+}] in the most proximal apical region and in the basal dendrites never exceeded the threshold of medium, and thus correlations with input patterns were indefinite. See Table 2 for corresponding Hay-model data.

**A** Region	Threshold
1: 0–200 μm	9.01e-06
2: 200–400 μm	1.26e-05
3: 400–600 μm	2.22e-05
4: 600–800 μm	4.93e-05
5: 800–1000 μm	0.000192
6: > 1000 μm	0.000767
7: basal	1.02e-05

Input location	N_{spikes}	Ca1	Ca2	Ca3	Ca4	Ca5	Ca6	Ca7
apic1	0.20	–	-0.09	-0.03	0.04	0.03	0.04	–
apic2	0.28	–	-0.09	0.05	0.04	0.04	0.04	–
apic3	0.38	–	0.09	0.26	0.06	0.04	0.05	–
apic4	0.20	–	-0.09	0.23	0.06	0.04	0.04	–
apic5	0.36	–	-0.09	0.31	0.54	0.55	0.52	–
apic6	0.34	–	0.09	0.34	0.59	0.60	0.63	–
basal	0.18	–	-0.09	-0.05	0.04	0.03	0.04	–

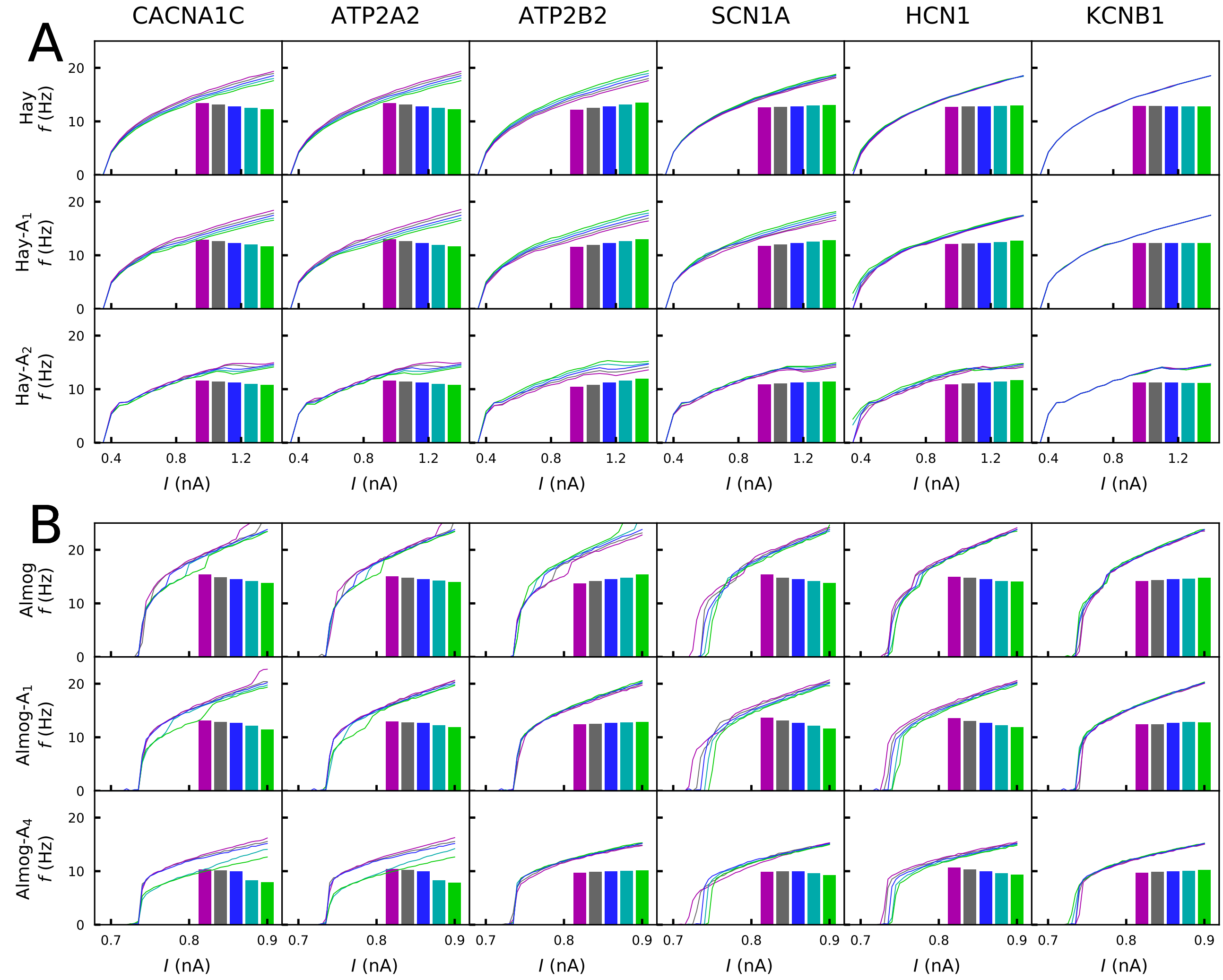

Figure S2: **Effects of the variants on steady-state firing behavior are qualitatively similar across the models.** The f-I curves according to unaltered and altered Hay (A) and Almog (B) models are plotted for each of the variants of Figure 2. The color coding is the same as in Figure 2. The insets show the averages of the f-I curves of unaltered and altered Hay (A) and Almog (B) models across the somatic current amplitudes (0.3–1.4 nA in (A) and 0.65–0.9 nA in (B)).

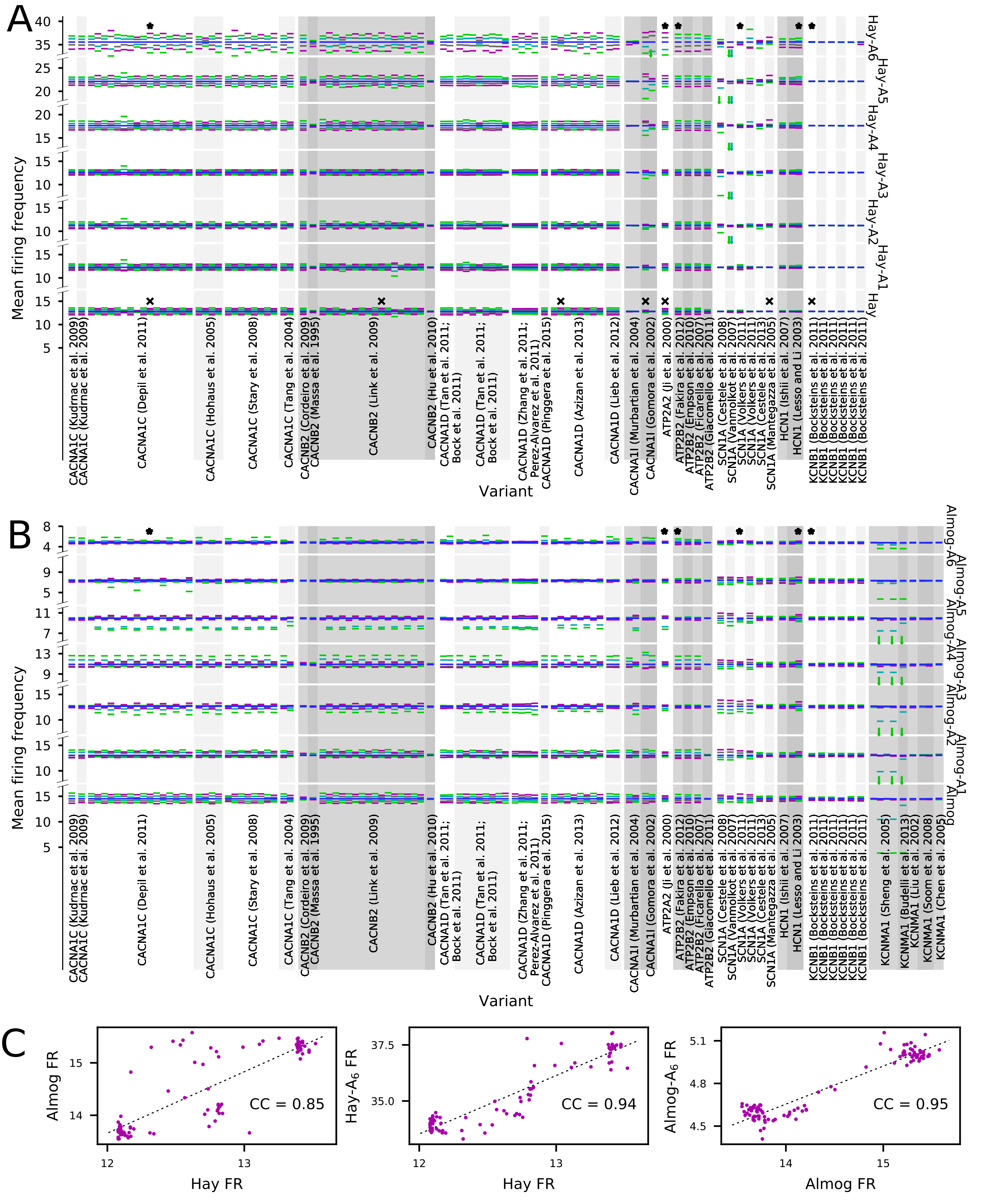

Figure S3: **A: Steady-state firing behavior of all variants according to the Hay model and the altered Hay models.** The y-axis data show the mean firing rates of Figure 2E for all variants of the Hay models, while the x-axis shows the different variants. Data outside the visible field reserved for the alternative Hay models (separated by discontinuities in the y-axis) are represented by arrows of the corresponding color (color coding as in Figure 2). The variants are ordered as in Table A2, and the variants used in Figure 2 are marked with stars (⋆). The variants chosen for the combination of variants in Figure 7 (representing the maximal increase in Hay-model f-I curve among the variants of the underlying gene) are marked with '×'. **B: Steady-state firing behavior of all variants according to the Almog model and the altered Almog models.** The y-axis data show the mean firing rates of Figure 2F for all variants of the Almog models, see panel (A). **C**: Illustration of the correlation coefficient of the f-I curve integrals between Hay and Almog model (left), Hay and Hay-A_6 model (middle), and Almog and Almog-A_6 model (right). Units in spikes/s.

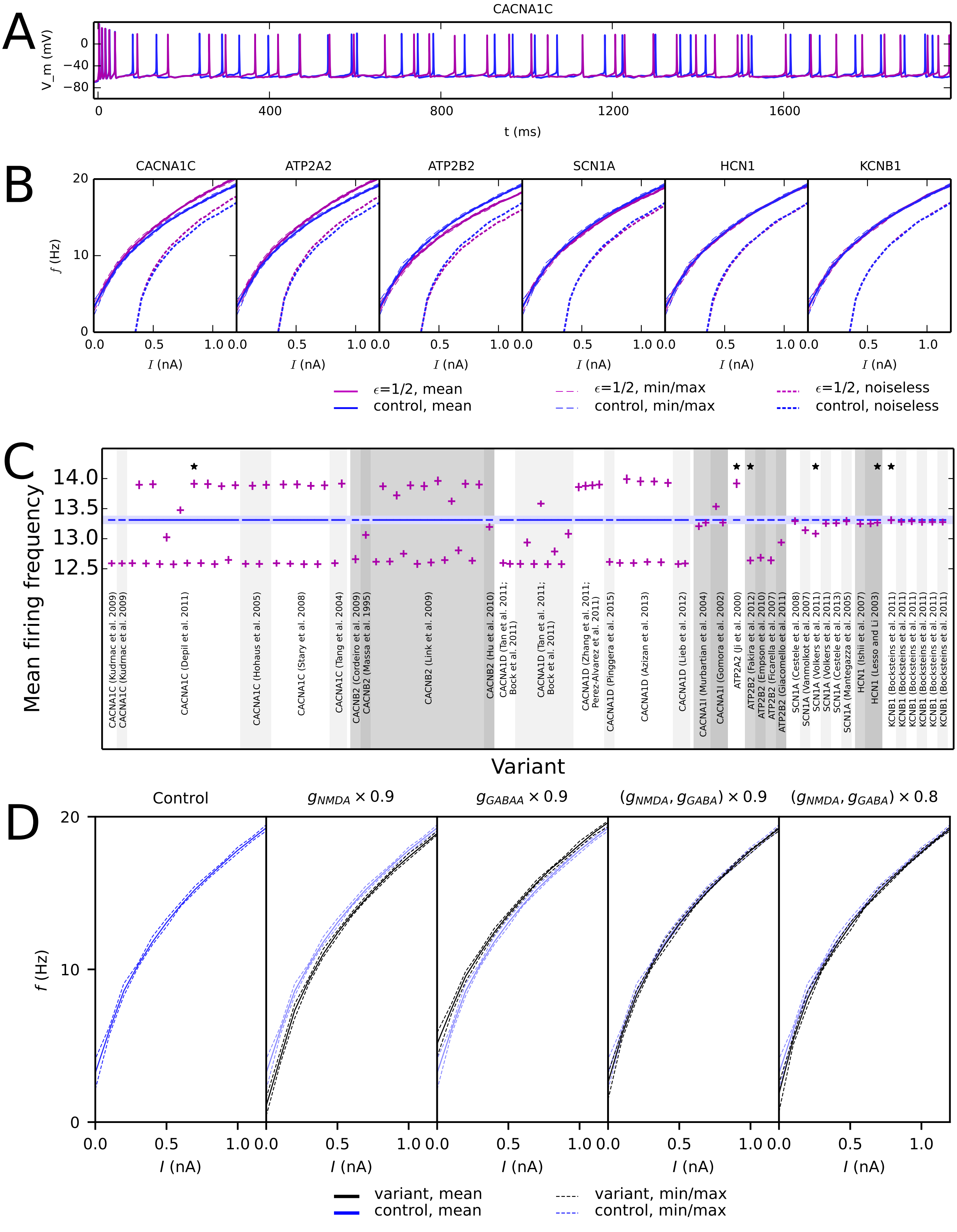

Figure S4: **Models of variant neurons with noisy inputs produce qualitatively similar predictions of altered gain as noiseless models, while variations of synaptic conductances alter the L5PC excitability without affecting the gain.** **A**: Membrane potential time series of the control Hay-model neuron and an example *CACNA1C* variant ($\epsilon = \frac{1}{2}$) in the presence of noise (spontaneous glutamatergic and GABAergic synaptic activation) and a 1.2 nA somatic DC (onset at 0 ms). **B**: Solid curves represent the mean noisy f-I data for the variants of Figure 2C (only $\epsilon = \frac{1}{2}$ scaling), surrounded by thin dashed curves that represent the STD of these data. Thick dashed curves represent the noiseless cases for comparison (from Figure 2C). **C**: Steady-state firing behavior of all variants according to the Hay model with noisy inputs. The shaded blue area shows the mean±SD for the control neuron, and the purple ticks show the mean±SD for the $\epsilon = \frac{1}{2}$ variants (ordered as in Table A2 and Figure S3). Variants of panel B are marked with stars. **D**: Variation of NMDA and GABA conductances, following the NMDA and GABA-hypofunction theories of SCZ, causes a steady increase or decrease of the f-I curve across the input current amplitudes. Blue curves show the control Hay model data from (B). Black curves show firing-rate data from cases where the NMDA-receptor (second panel from left), GABA-receptor (middle panel) conductance or both (second two panels from right) are decreased. Solid curves represent the mean noisy f-I data, surrounded by thin dashed curves that represent the STD of these data.

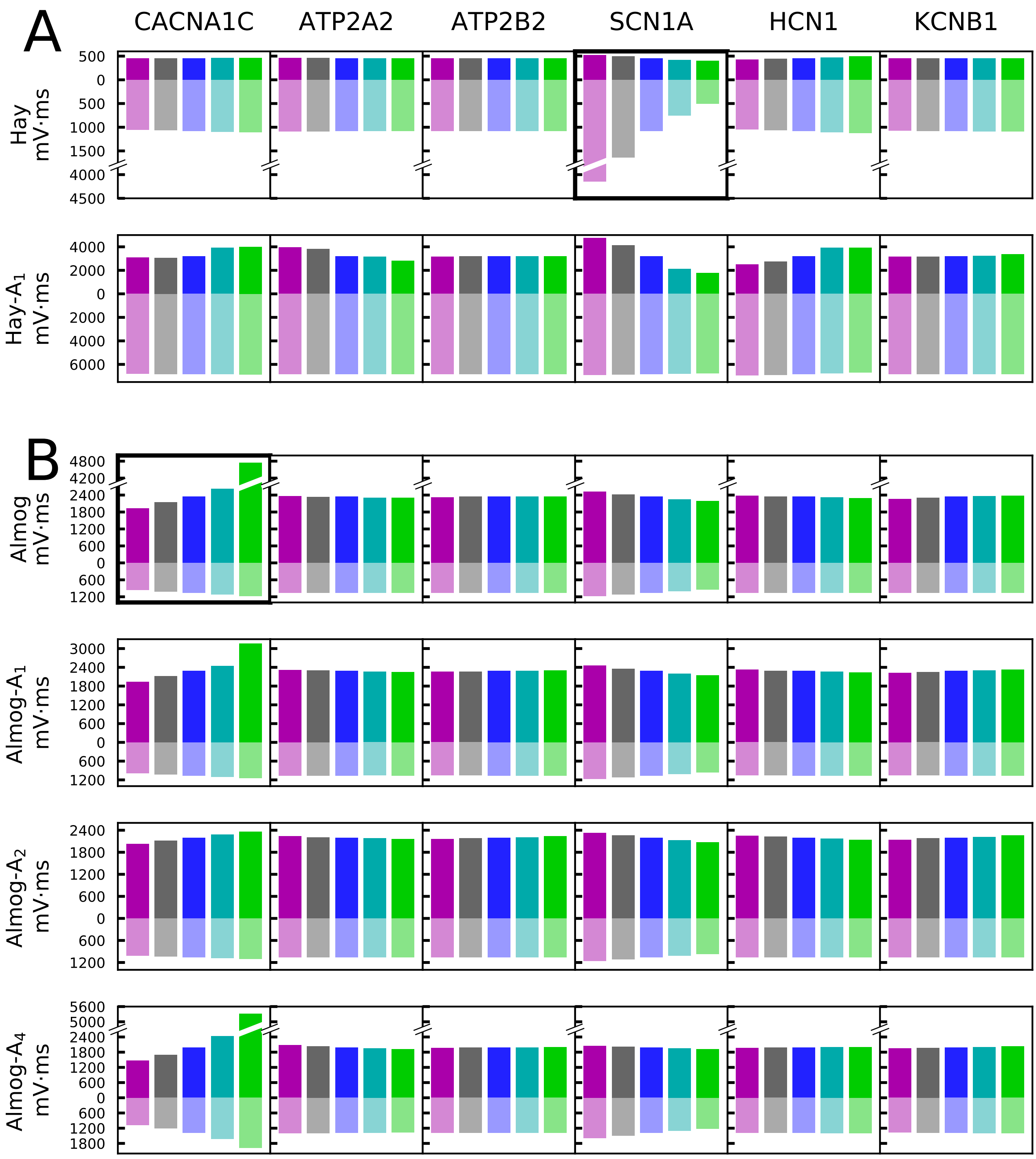

Figure S5: **Variants affect the sensitivity to coincidence of somatic and apical stimuli during up and down states**. The y-axes show the integrals of the up- (above zero, strong colors) and down-state (below zero, dim colors) temporal windows of Figure 3D–E across the tested ISIs (-30 to 70 ms) for the variants of Figure 2. Blue: control neuron, other colors: different scalings of the variants of Figure 2. See Figure 3F–G for details. The panels with thick borders represent the data from the variants shown in Figure 3A–B.

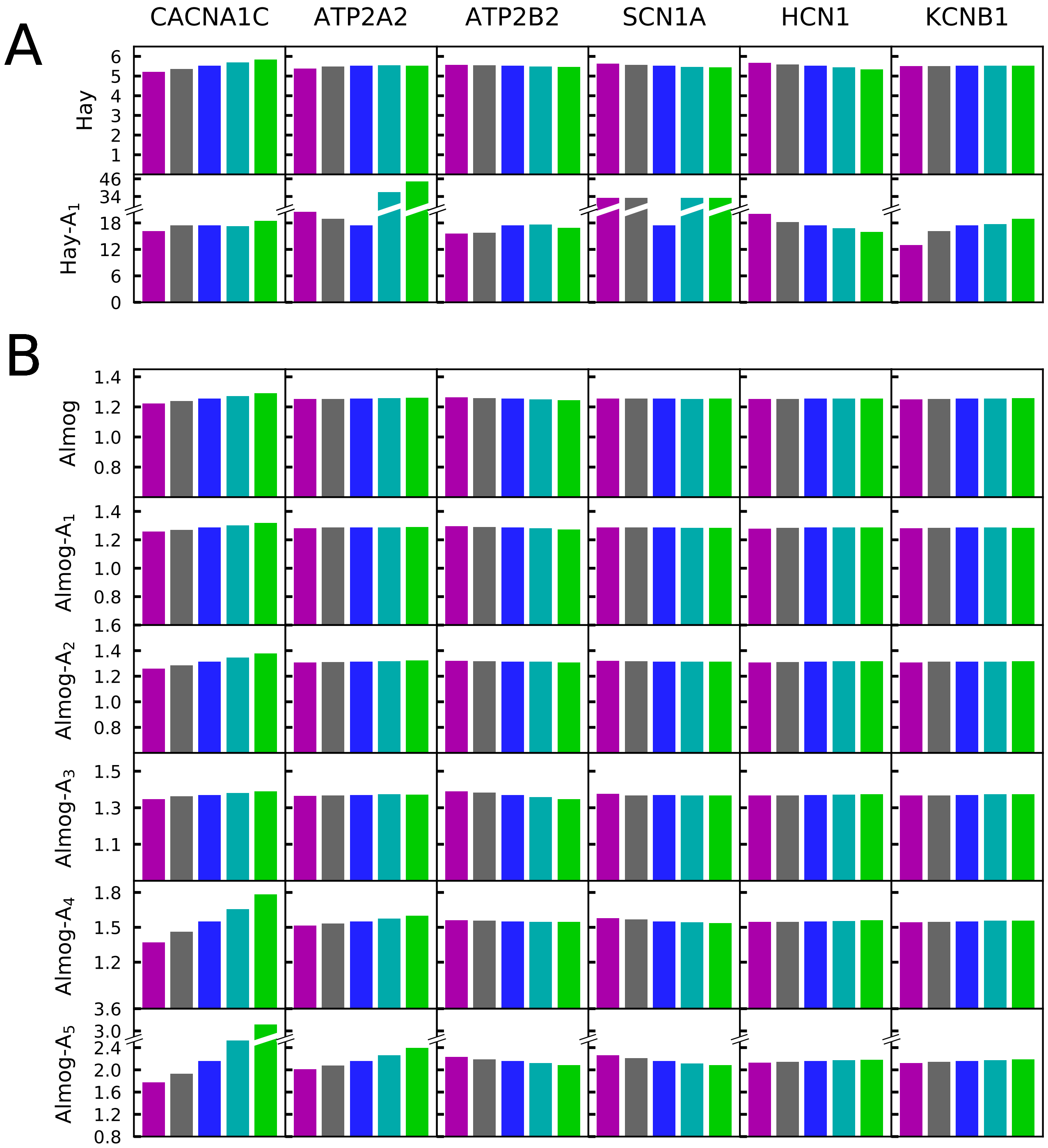

Figure S6: **Variants affect the L5PC adaptation by a prepulse**. The maximal threshold conductance factor from the adaptation curves of Figure 5C–D, excluding the first 40 ms. **A**: Adaptation data from the Hay-model and Hay-A_1-model neurons (see Figure 5E). **B**: Adaptation data from the unaltered and altered Almog models (see Figure 5F). Colors as in Figure 2. The first column of panels are the same as in Figure 5E–F, re-illustrated here for consistency.

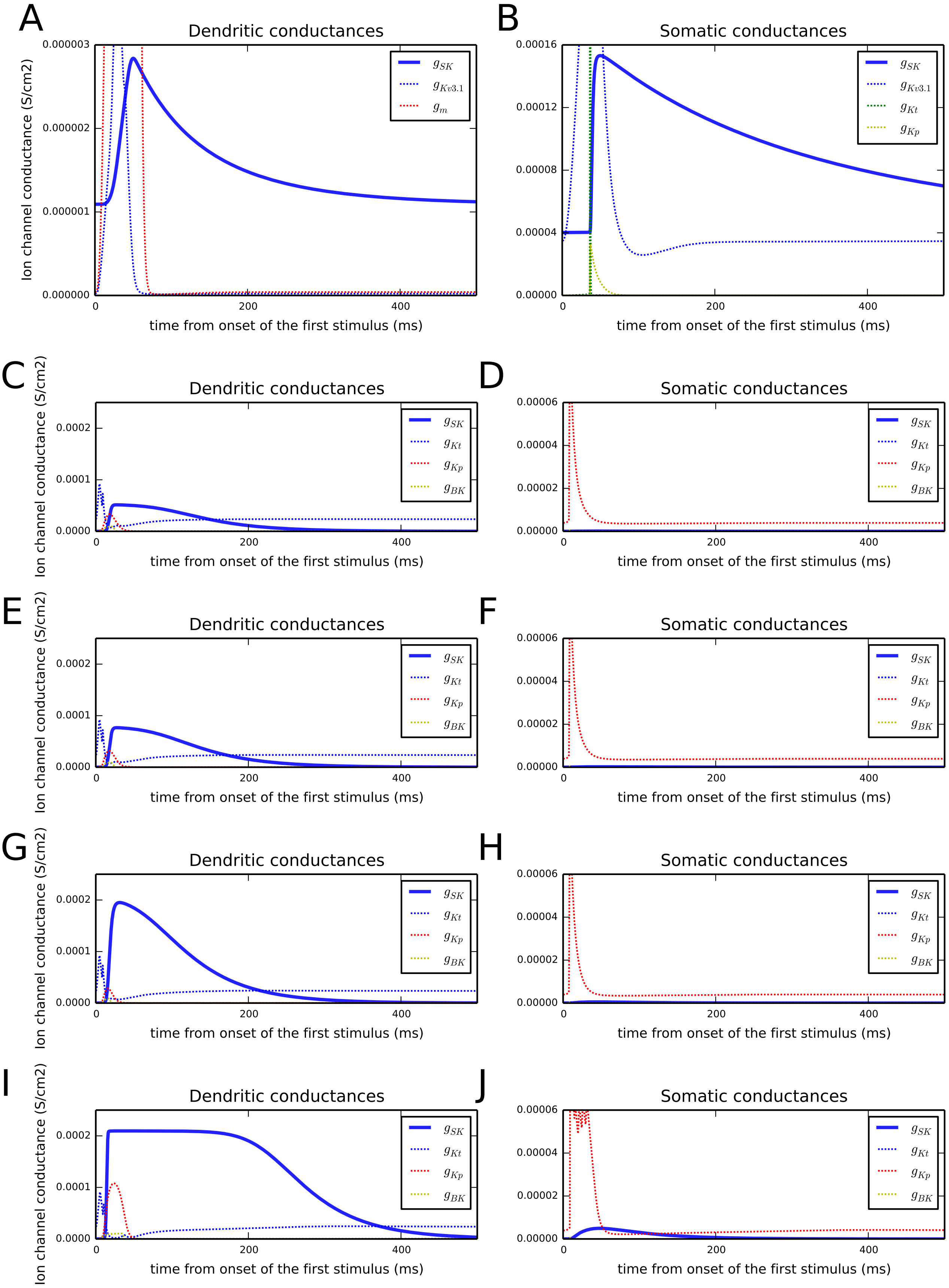

Figure S7: **K$^+$ currents in the apical dendrite and in the soma following a suprathreshold activation of 3000 synapses distributed along the apical dendrite as predicted by different L5PC models**. **A,C,E,G,I**: Currents recorded in the apical dendrite, 620 μm from the soma. **B,D,F,H,J**: Currents recorded in the soma. The models used were the Hay model (A–B), the Almog model (C–D), the Almog-A$_2$ model (E–F), the Almog-A$_4$ model (G–H), and the Almog-A$_6$ model (I–J). Only data from control L5PCs are shown here (no genetic variants were introduced).

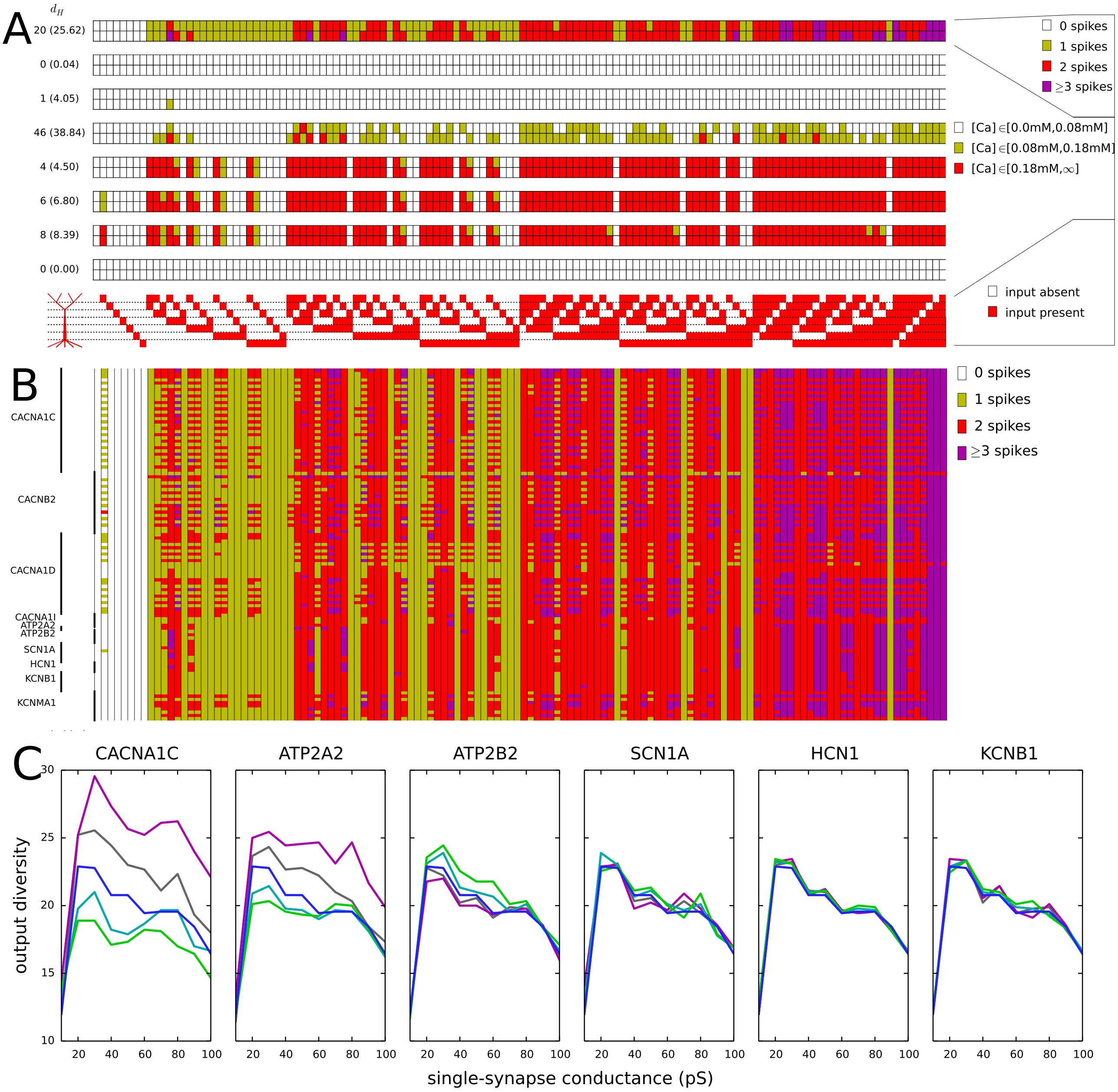

Figure S8: **Variants affect the coding of information in Almog-model L5PCs**. The experiment of Figure 6 was repeated using the Almog model. **A**: The maximal conductance of each synaptic input (when activated) was half of threshold value in the underlying dendritic region for inducing a spike (Table S5A). The eight panels show the numbers of spikes induced (top) and the Ca^{2+} response in the apical regions 1–6 (six panels below) and in the basal dendritic region (bottom panel) for each of the 128 input combinations. As the range of Ca^{2+} was different in the Almog model than in the Hay model, different thresholds for low ($[Ca^{2+}] < 0.08$ mM), medium ($[Ca^{2+}] < 0.18$ mM) and high ($[Ca^{2+}] \geq 0.18$ mM) Ca^{2+} levels were chosen for the Almog model. Upper rows of these panels show the control L5PC data, and the lower rows show the data from the *CACNA1C* variant of Figure 2. **B**: Compiled spike-number data from all $\epsilon = \frac{1}{2}$ variants of Table A2. The Almog-model L5PC is activated as in panel (A). **C**: Output diversities of the variants of Figure 2 according to the Almog model.

References

[1] Michaela Kudrnac, Stanislav Beyl, Annette Hohaus, Anna Stary, Thomas Peterbauer, Eugen Timin, and Steffen Hering. Coupled and independent contributions of residues in IS6 and IIS6 to activation gating of CaV1.2. *J Biol Chem*, 284(18):12276–12284, 2009.

[2] Katrin Depil, Stanislav Beyl, Anna Stary-Weinzinger, Annette Hohaus, Eugen Timin, and Steffen Hering. Timothy mutation disrupts the link between activation and inactivation in cav1. 2 protein. *J Biol Chem*, 286(36):31557–31564, 2011.

[3] Annette Hohaus, Stanislav Beyl, Michaela Kudrnac, Stanislav Berjukow, Eugen N Timin, Rainer Marksteiner, Marion A Maw, and Steffen Hering. Structural determinants of l-type channel activation in segment iis6 revealed by a retinal disorder. *J Biol Chem*, 280(46):38471–38477, 2005.

[4] Anna Stary, Michaela Kudrnac, Stanislav Beyl, Annette Hohaus, Eugen Timin, Peter Wolschann, H Robert Guy, and Steffen Hering. Molecular dynamics and mutational analysis of a channelopathy mutation in the iis6 helix of cav1. 2. *Channels*, 2(3):216–223, 2008.

[5] Zhen Zhi Tang, Mui Cheng Liang, Songqing Lu, Dejie Yu, Chye Yun Yu, David T Yue, and Tuck Wah Soong. Transcript scanning reveals novel and extensive splice variations in human l-type voltage-gated calcium channel, cav1. 2 α1 subunit. *J Biol Chem*, 279(43):44335–44343, 2004.

[6] Bao Zhen Tan, Fengli Jiang, Ming Yeong Tan, Dejie Yu, Hua Huang, Yiru Shen, and Tuck Wah Soong. Functional characterization of alternative splicing in the c terminus of l-type cav1. 3 channels. *J Biol Chem*, 286(49):42725–42735, 2011.

[7] Gabriella Bock, Mathias Gebhart, Anja Scharinger, Wanchana Jangsangthong, Perrine Busquet, Chiara Poggiani, Simone Sartori, Matteo E Mangoni, Martina J Sinnegger-Brauns, Stefan Herzig, et al. Functional properties of a newly identified c-terminal splice variant of cav1. 3 l-type ca2+ channels. *J Biol Chem*, 286(49):42736–42748, 2011.

[8] Q. Zhang, V. Timofeyev, H. Qiu, L. Lu, N. Li, A. Singapuri, C. L. Torado, H. S. Shin, and N. Chiamvimonvat. Expression and roles of Cav1.3 (α1D) L-type Ca2+ channel in atrioventricular node automaticity. *J Mol Cell Cardiol*, 50(1):194–202, 2011.

[9] Alberto Pérez-Alvarez, Alicia Hernández-Vivanco, Jose Carlos Caba-González, and Almudena Albillos. Different roles attributed to cav1 channel subtypes in spontaneous action potential firing and fine tuning of exocytosis in mouse chromaffin cells. *J Neurochem*, 116(1):105–121, 2011.

[10] Alexandra Pinggera, Andreas Lieb, Bruno Benedetti, Michaela Lampert, Stefania Monteleone, Klaus R Liedl, Petronel Tuluc, and Jörg Striessnig. Cacna1d de novo mutations in autism spectrum disorders activate cav1. 3 l-type calcium channels. *Biol Psychiatry*, 77(9):816–822, 2015.

[11] Elena AB Azizan, Hanne Poulsen, Petronel Tuluc, Junhua Zhou, Michael V Clausen, Andreas Lieb, Carmela Maniero, Sumedha Garg, Elena G Bochukova, Wanfeng Zhao, et al. Somatic mutations in atp1a1 and cacna1d underlie a common subtype of adrenal hypertension. *Nat Genet*, 45(9):1055–1060, 2013.

[12] Andreas Lieb, Anja Scharinger, Simone Sartori, Martina J Sinnegger-Brauns, and Jörg Striessnig. Structural determinants of cav1. 3 l-type calcium channel gating. *Channels*, 6(3):197–205, 2012.

[13] Jonathan M Cordeiro, Mark Marieb, Ryan Pfeiffer, Kirstine Calloe, Elena Burashnikov, and Charles Antzelevitch. Accelerated inactivation of the L-type calcium current due to a mutation in CACNB2b underlies brugada syndrome. *J Mol Cell Cardiol*, 46(5):695–703, 2009.

[14] Enrique Massa, Kevin M Kelly, David I Yule, Robert L MacDonald, and Michael D Uhler. Comparison of fura-2 imaging and electrophysiological analysis of murine calcium channel alpha 1 subunits coexpressed with novel beta 2 subunit isoforms. *Mol Pharmacol*, 47(4):707–716, 1995.

[15] Sabine Link, Marcel Meissner, Brigitte Held, Andreas Beck, Petra Weissgerber, Marc Freichel, and Veit Flockerzi. Diversity and developmental expression of L-type calcium channel β2 proteins and their influence on calcium current in murine heart. *J Biol Chem*, 284(44):30129–30137, 2009.

[16] Dan Hu, Hector Barajas-Martinez, Vladislav V Nesterenko, Ryan Pfeiffer, Alejandra Guerchicoff, Jonathan M Cordeiro, Anne B Curtis, Guido D Pollevick, Yuesheng Wu, Elena Burashnikov, et al. Dual variation in scn5a and cacnb2b underlies the development of cardiac conduction disease without brugada syndrome. *Pacing Clin Electrophysiol*, 33(3):274–285, 2010.

[17] Janet Murbartián, Juan Manuel Arias, and Edward Perez-Reyes. Functional impact of alternative splicing of human T-type Cav3.3 calcium channels. *J Neurophysiol*, 92(6):3399–3407, 2004.

[18] Juan Carlos Gomora, Janet Murbartian, Juan Manuel Arias, Jung-Ha Lee, and Edward Perez-Reyes. Cloning and expression of the human t-type channel ca v 3.3: insights into prepulse facilitation. *Biophys J*, 83(1):229–241, 2002.

[19] Yong Ji, M Jane Lalli, Gopal J Babu, Yanfang Xu, Darryl L Kirkpatrick, Lynne H Liu, Nipavan Chiamvimonvat, Richard A Walsh, Gary E Shull, and Muthu Periasamy. Disruption of a single copy of the serca2 gene results in altered ca2+ homeostasis and cardiomyocyte function. *J Biol Chem*, 275(48):38073–38080, 2000.

[20] Amanda K Fakira, Lawrence D Gaspers, Andrew P Thomas, Hong Li, Mohit R Jain, and Stella Elkabes. Purkinje cell dysfunction and delayed death in plasma membrane calcium atpase 2-heterozygous mice. *Mol Cell Neurosci*, 51(1):22–31, 2012.

[21] Ruth M Empson, Walther Akemann, and Thomas Knöpfel. The role of the calcium transporter protein plasma membrane calcium ATPase PMCA2 in cerebellar Purkinje neuron function. *Funct Neurol*, 25(3):153, 2010.

[22] R Ficarella, F Di Leva, M Bortolozzi, S Ortolano, F Donaudy, M Petrillo, S Melchionda, A Lelli, T Domi, L Fedrizzi, et al. A functional study of plasma-membrane calcium-pump isoform 2 mutants causing digenic deafness. *Proc Natl Acad Sci USA*, 104(5):1516–1521, 2007.

[23] Marta Giacomello, Agnese De Mario, Raffaele Lopreiato, Simona Primerano, Mara Campeol, Marisa Brini, and Ernesto Carafoli. Mutations in pmca2 and hereditary deafness: a molecular analysis of the pump defect. *Cell Calcium*, 50(6):569–576, 2011.

[24] Sandrine Cestèle, Paolo Scalmani, Raffaella Rusconi, Benedetta Terragni, Silvana Franceschetti, and Massimo Mantegazza. Self-limited hyperexcitability: Functional effect of a familial hemiplegic migraine mutation of the Nav1.1 (SCN1A) na+ channel. *J Neurosci*, 28(29):7273–7283, 2008.

[25] Kaate RJ Vanmolkot, Elena Babini, Boukje de Vries, Anine H Stam, Tobias Freilinger, Gisela M Terwindt, Lisa Norris, Joost Haan, Rune R Frants, Nabih M Ramadan, et al. The novel p.L1649Q mutation in the SCN1A epilepsy gene is associated with familial hemiplegic migraine: genetic and functional studies. *Hum Mutat*, 28(5):522–522, 2007.

[26] Linda Volkers, Kristopher M Kahlig, Nienke E Verbeek, Joost HG Das, Marjan JA van Kempen, Hans Stroink, Paul Augustijn, Onno van Nieuwenhuizen, Dick Lindhout, Alfred L George, et al. Nav1. 1 dysfunction in genetic epilepsy with febrile seizures-plus or dravet syndrome. *European J Neurosci*, 34(8):1268–1275, 2011.

[27] Sandrine Cestèle, Angelo Labate, Raffaella Rusconi, Patrizia Tarantino, Laura Mumoli, Silvana Franceschetti, Grazia Annesi, Massimo Mantegazza, and Antonio Gambardella. Divergent effects of the t1174s scn1a mutation associated with seizures and hemiplegic migraine. *Epilepsia*, 54(5):927–935, 2013.

[28] Massimo Mantegazza, Antonio Gambardella, Raffaella Rusconi, Emanuele Schiavon, Ferdinanda Annesi, Rita Restano Cassulini, Angelo Labate, Sara Carrideo, Rosanna Chifari, Maria Paola Canevini, et al. Identification of an nav1.1 sodium channel (scn1a) loss-of-function mutation associated with familial simple febrile seizures. *Proc Natl Acad Sci USA of the United States of America*, 102(50):18177–18182, 2005.

[29] Takahiro M Ishii, Noriyuki Nakashima, and Harunori Ohmori. Tryptophan-scanning mutagenesis in the s1 domain of mammalian hcn channel reveals residues critical for voltage-gated activation. *J Physiol (Lond)*, 579(2):291–301, 2007.

[30] Heinte Lesso and Ronald A Li. Helical secondary structure of the external s3-s4 linker of pacemaker (hcn) channels revealed by site-dependent perturbations of activation phenotype. *J Biol Chem*, 278(25):22290–22297, 2003.

[31] E Bocksteins, N Ottschytsch, J-P Timmermans, AJ Labro, and DJ Snyders. Functional interactions between residues in the S1, S4, and S5 domains of Kv2.1. *Eur Biophys J*, 40(6):783–793, 2011.

[32] Jian-Zhong Sheng, Aalim Weljie, Lusia Sy, Shizhang Ling, Hans J Vogel, and Andrew P Braun. Homology modeling identifies c-terminal residues that contribute to the ca2+ sensitivity of a bkca channel. *Biophys J*, 89(5):3079–3092, 2005.

[33] Gonzalo Budelli, Yanyan Geng, Alice Butler, Karl L Magleby, and Lawrence Salkoff. Properties of slo1 k+ channels with and without the gating ring. *Proc Natl Acad Sci USA*, page 201313433, 2013.

[34] Xiaojin Liu, Yongchan Chang, Peter H Reinhart, and Harald Sontheimer. Cloning and characterization of glioma bk, a novel bk channel isoform highly expressed in human glioma cells. *J Neurosci*, 22(5):1840–1849, 2002.

[35] Malle Soom, Guido Gessner, Heike Heuer, Toshinori Hoshi, and Stefan H Heinemann. A mutually exclusive alternative exon of slo 1 codes for a neuronal bk channel with altered function. *Channels*, 2(4):278–282, 2008.

[36] Lie Chen, Lijun Tian, Stephen H-F MacDonald, Heather McClafferty, Martin SL Hammond, Jean-Marc Huibant, Peter Ruth, Hans-Guenther Knaus, and Michael J Shipston. Functionally diverse complement of large conductance calcium-and voltage-activated potassium channel (bk) α-subunits generated from a single site of splicing. *J Biol Chem*, 280(39):33599–33609, 2005.
